# Genomic architecture and evolutionary relationship of BA.2.75: A Centaurus subvariant of Omicron SARS-CoV-2

**DOI:** 10.1371/journal.pone.0281159

**Published:** 2023-05-24

**Authors:** Atia Basheer, Imran Zahoor, Tahir Yaqub

**Affiliations:** 1 Genetics and Genomics Laboratory, Dept. of Animal Breeding and Genetics, University of Veterinary and Animal Sciences, Lahore, Pakistan; 2 Institute of Microbiology, University of Veterinary and Animal Sciences, Lahore, Pakistan; Alagappa University, INDIA

## Abstract

In this study, we explored the genomic architecture and phylogenomic relationship of BA.2.75, a subvariant of Omicron SARS-CoV-2. A set of 1468 whole-genome sequences of BA.2.75, submitted by 28 countries worldwide were retrieved from GISAID and used for finding genomic mutations. Moreover, the phylogenetic analysis of BA.2.75 was performed by using 2948 whole-genome sequences of all sub-variants of Omicron along with the Delta variant of SAS-CoV-2. We detected 1885 mutations, which were further grouped into 1025 missense mutations, 740 silent mutations, 72 mutations in non-coding regions, 16 in-frame deletions, 02 in-frame insertions, 8 frameshift deletions, 8 frameshift insertions and 14 stop-gained variants. Additionally, we also found 11 characteristic mutations having a prevalence of 81–99% and were not observed in any of the previously reported variant of SARS-CoV-2. Out of these mutations K147E, W152R, F157L, E210V, V213G, G339H were found in the NTD, and G446S & N460K in the RBD region of the Spike protein, whereas S403L and T11A were present in the NSP3, and E protein respectively. The phylogenetic relationship of this variant revealed that BA.2.75 is descended from the Omicron sub-variant BA.5. This evolutionary relationship suggests that the surge of BA.5 infections can reduce the severity of the infections accredited to BA.2.75. These findings would also improve our knowledge and understanding that how genetic similarities in different variants of SARS-CoV-2 can prime the immune system to fight off the infection caused by one subvariant, after defeating the other.

## Introduction

The Omicron variant of SARS-CoV-2 was first identified in Botswana, and South Africa in the early November 2021. On November 24, 2021, it was reported to the World Health Organization (WHO) and eventually declared as a variant of concern (VOC) on November 26, 2021 [1]. Though Omicron replicates about 70-times faster than the delta variant in the bronchi of infected individuals, but there are some evidences that it is less severe than previous strains/variants, especially when compared to the delta variant [2]. However, it continued to spread rapidly into multiple sub-lineages which differ significantly from the previously reported variants of SARS-CoV-2. From a global pandemic perspective, the Omicron variant has shown super transmissibility by rapidly replacing the Delta variant which had been the dominant epidemic variant in many countries until the end of 2021 [3]. And by August 8, 2022, over 2 million Omicron sequences had been submitted to GISAID which were further categorized into different sub-lineages, such as BA.1, BA.1.1, BA.2, BA.2.12.1, BA.2.3, BA.2.9, BA.3, BA.4, BA.5. and BA.2.75 [4]. These sub-lineages also exhibited distinct capabilities of transmission and immune evasion. However, since February 2022, BA.2, which appeared more transmissible has become the most dominant and transmissible strain in many countries, such as South Africa, United Kingdom and India by rapidly replacing BA.1 and BA.1.1 [5]. Likewise, BA.2.12.1 subvariant of Omicron SARS-CoV-2 also showed enhanced transmissibility and became the dominant variant in United States in the early months of 2022 [3].

Moreover, the BA.2.75, first detected in India, is an emerging sub-lineage of the Omicron variant which has also been reported from a large number of countries, worldwide. Its rapid spread and transmission are being monitored by the WHO and CDC, jointly [6]; and it has been unofficially declared as a Centaurus variant [7]. As emergence of this variant is a very recent development, so comprehensive information about it is not available. However, according to the available information, the symptoms of this subvariant are very similar to that of the common cold, and it has very high transmissibility [8].

The SARS-CoV-2 is constantly mutating and leading to the emergence of new variants which are usually more contagious than Wuhan-Hu-1 [9, 10]. However, the likelihood of high or low severity of different variant varies which make it hard to predict their ultimate consequences. According to the latest statistics, the newly emerged sub-lineage Omicron BA.2.75 has become the dominant variant globally [11]. It has multiple mutations in the Spike protein which perhaps enhance its transmissibility and infectivity by enabling it to bind more efficiently to the host cell receptors. It is further speculated that the mutations of BA.2.75 may reduce the abilities of the antibodies to bind or neutralize the virus. Hence, there are growing concerns that this virus can spread very quickly by evading the immunity produced through vaccination or prior infections [3]. Hence, there is an urgent need to explore and understand the genomic architecture of BA.2.75 not only to find out its characteristic mutations which differentiate it from other sub-variants but also to figure out some pathways to initiate the host immune response and develop some protective immunity against it.

## Methods

For this study, whole-genome sequences of 1675 global samples of BA.2.75 subvariant of Omicron SARS-CoV-2 were retrieved from GISAID (Global Initiative on Sharing Avian Influenza Data) [12], on 8^th^ August 2022. The first genome of BA.2.75 sub-variant of Omicron SARS-CoV-2 was submitted to GISAID in May 2022, and up till 8^th^ August 2022, 1675 cases of this variant had been sequenced and submitted to GISAID. These sequences were submitted to GISAID by the 28 countries including India (1127), USA (78), Singapore (42), Japan (37), United Kingdom (32), Canada (31), Australia (26), Nepal (16), Denmark (13), Israel (11), Austria (9), Indonesia (8), New Zeeland (6), Germany (6), Luxembourg (4), South Korea (4), France (4), Thailand (2). Italy (2), Peru (2), Chile (1), Martinique (1), Turkey (1), Slovakia (1), Cambodia (1), China (1) and Slovenia (1). The retrieved genomic sequences were aligned through MAFFT (v7.480) by using L-INS-I alignment and setting data type as nucleic acids with a gap extended penalty of 0.123 and opening penalties of 1.53 [13]. The Wuhan-Hu-1 sequence (NC_045512.2) was used as a reference genome while aligning the sequence data. The alignment of the genomic sequences led to the removal of 207 low quality sequences and the resulting file was left with a set of 1468, high coverage, genomic sequences GISAID considers genomes with length of >29,000 nucleotides as complete and assigns them the high coverage label when there are less than 1% of undefined bases. For the remaining 1468 good quality genome sequences, all the relevant information including their unique identifiers, collection & submission date, and submitting lab information are shown in supplementary materials (S1 Table). The aligned and filtered sequence file was used as an input to the Coronapp web application to obtain the nucleotide variations [14]. All the mutations found in the structural, non-structural, and accessory proteins were mapped on the SARS-CoV-2 genome by using the Corona Antiviral Research Database (CoV-RDB) [15]. Additionally, the CoV-RDB [15] was also used for the comparison of BA.2.75 mutations with other sub-variants of Omicron SARS-CoV-2. The amino acid sequence of Spike protein was downloaded from the GenBank [16]. However, the three-dimensional (3D) structures of Spike protein used in this work was extracted from the Protein Data Bank (https://www.rcsb.org/), denoted as 6VYB, and its 3D-structured graph highlighting mutations was developed by using PyMOL [17].

For the construction of the phylogenetic tree, 2948 whole-genome sequences of different variants of SARS-CoV-2 retrieved from the GISAID database were used. And the phylogenetic tree was constructed by using the NextStrain’s Augur pipeline [18]. Sequences were again aligned to the SARS-CoV-2 reference genome (NC_045512.2) by using MAFFT [19] and a time-resolved phylogenetic tree was constructed with IQ-Tree [20] and TreeTime [21] under the generalized time reversible (GTR) substitution model [22] and was visualized with auspice [18].

## Results

**Fig 1:**
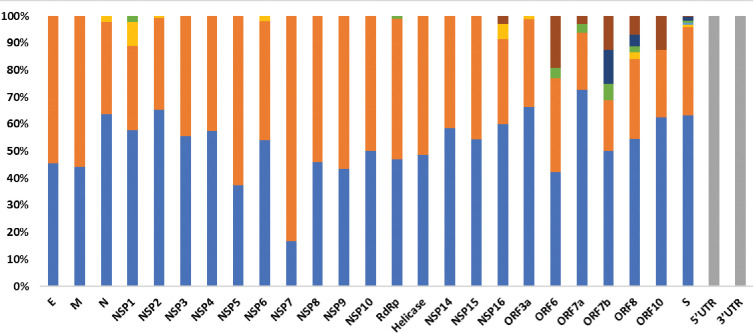
Stacked bar-chart of mutations percentages observed in different proteins of the BA.2.75 subvariant of Omicron SARS-CoV-2.

**Fig 2:**
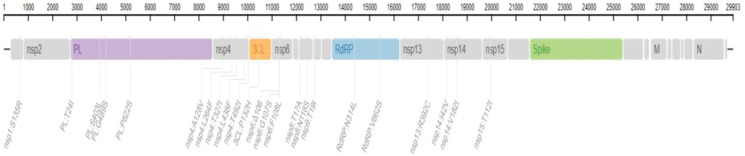
Pictorial view of 23 mutations present in the Non-structural proteins of BA.2.75 subvariant of Omicron SARS-CoV-2.

In total, we detected 1885 mutations, which were categorized as 1025 amino acids changing (missense) mutations, 740 silent mutations, 72 mutations in the non-coding regions, 16 in-frame deletions, 44 frameshift deletions, 2 in-frame insertions, 8 frameshift deletion, 8 frameshift insertions, and 10 stop-gained variants (Table 1, Fig 1). Out of these mutations, 605 were present in the ORF1ab which transcribes into 16 non-structural proteins (NSPs). However, among theORF1ab proteins, NSP3 had the largest number of missense (207) and silent mutations (165) (Table 1, Fig 2) which is also common with the mutation pattern of earlier reported variants of SARS-CoV-2 [23]. The second largest number of mutations, among the NSPs, were found in the NSP2 protein which had 79 missense and 41 silent mutations followed by RdRp protein (57 missense, 60 silent and 1 frameshift deletion) (Table 1, Fig 2). In case of NSP3 G489S, T241I, S403L, and T183I had 99% frequency while the prevalence of P822S mutation was 98% (Table 2). However, P314L, T492I and P132H mutations present in the RdRp, NSP4 and NSP5 protein respectively were the most common (100% prevalence) missense mutations which were present in all the global samples. Likewise, T1121I mutation in NSP15, R392C in Helicase, G662S in RdRp and L264F & L438F in NSP4 had 99% incidence globally. However, T327I in NSP4, N118S in NSP8, had 97% incidence while T19I in NSP9 had 95% incidence globally, whereas S106del in NSP6 had 72% prevalence (Table 2).

**Table 1:** Details of mutations found in different proteins of BA.2.75 variant of Omicron SARS-CoV-2.

Protein	Missense	Silent SNP	Non-coding region Mutation	In-frame		Frameshift		Stop gained	Total
				deletion	Insertion	Deletion	Insertion		
E	5	6	-	-	-	-	-	-	11
M	23	29	-	-	-	-	-	-	52
N	89	48	-	3	-		-	-	140
NSP1	26	14	-	4	-	1	-	-	45
NSP2	79	41	-	1	-	-	-	-	121
NSP3	207	165	-	-	-	-	-	-	372
NSP4	46	34	-	-	-	-	-	-	80
NSP5	19	32	-	-	-	-	-	-	51
NSP6	26	21	-	1	-	-	-	-	48
NSP7	2	10	-	-	-	-	-	-	12
NSP8	11	13	-	-	-	-	-	-	24
NSP9	10	13	-	-	-	-	-	-	23
NSP10	7	7	-	-	-	-	-	-	14
RdRp	54	60	-	-	-	1	-	-	115
Helicase	37	39	-	-	-	-	-	-	76
NSP14	41	29	-	-	-	-	-	-	70
NSP15	19	16	-	-	-	-	-	-	35
NSP16	21	11	-	2	-	-	-	1	35
ORF3a	51	25	-	1	-	-	-	-	77
ORF6	11	9	-	-	-	1	-	5	26
ORF7a	24	7	-	-	-	1	-	1	33
ORF7b	8	3	-	-	-	1	2	2	16
ORF8	24	13	-	1	-	1	2	3	44
ORF10	5	2	-	-	-	-	-	1	8
S	180	93	-	3	2	2	4	1	285
5’UTR	0	0	33	-	-	-	-	-	33
3’UTR	0	0	39	-	-	-	-	-	39
**Total**	**1025**	**740**	**72**	**16**	**2**	**8**	**8**	**14**	**1885**

**Table 2:** Major mutations found in the genome of BA.2.75 variant of Omicron SARS-CoV-2.

Sr. No	Genomic Change	Protein	Amino acid change	Type of mutation	Global %
1.	23403A>G	S	D614G	Missense	100
2.	23525C>T	S	H655Y	Missense	100
3.	23599T>G	S	N679K	Missense	100
4.	23604C>A	S	P681H	Missense	99
5.	25000C>T	S	D1146D	Silent	99
6.	22577GG>CA	S	G339H	Missense	99
7.	22775G>A	S	D405N	Missense	97
8.	22331G>A	S	G257S	Missense	97
9.	24469T>A	S	N969K	Missense	97
10.	24424A>T	S	Q954H	Missense	96
11.	23948G>T	S	D796Y	Missense	96
12.	12741C>T	S	T19I	Missense	95
13.	23854C>A	S	N764K	Missense	95
14.	22942T>G	S	N460K	Missense	94
15.	22786A>C	S	R408S	Missense	93
16.	22898G>A	S	G446S	Missense	93
17.	23013A>C	S	E484A	Missense	92
18.	22992G>A	S	S477N	Missense	92
19.	22882T>G	S	N440K	Missense	92
20.	22995C>A	S	T478K	Missense	92
21.	22190A>G	S	I210V	Missense	92
22.	22200T>G	S	V213G	Missense	92
23.	22674C>T	S	S371F	Missense	91
24	23075T>C	S	Y505H	Missense	91
25.	23063A>T	S	N501Y	Missense	91
26	23055A>G	S	Q498R	Missense	89
27.	22679T>C	S	S373P	Missense	89
28.	22813G>T	S	K417N	Missense	88
29.	22686C>T	S	S375F	Missense	88
30.	22033C>A	S	F157L	Missense	88
31.	22016T>C	S	W152R	Missense	86
32.	22688A>G	S	T376A	Missense	86
33.	22001A>G	S	K147E	Missense	81
34.	21633TACCCCCTG	S	L24	Deletion	78
35.	21987G>A	S	G142D	Missense	56
36.	23283A>T	S	D574V	Missense	17
37.	22720A>G	S	K386K	Silent	6
38.	21641G>T	S	A27S	Missense	6
39.	27807C>T	ORF7b	L17L	Silent	100
40.	27527C>T	ORF7a	P45L	Missense	5
41.	27259A>C	ORF6	M19M	Silent	99
42.	27382GAT>CTC	ORF6	D61L	Missense	99
43.	25416C>T	ORF3a	F8F	Silent	100
44.	26060C>T	ORF3a	T223I	Missense	100
45.	2911T>C	ORF3a	T64T	Silent	99
46.	12880C>T	NSP9	I65I	Silent	99
47.	12741C>T	NSP9	T19I	Missense	95
48.	12444A>G	NSP8	N118S	Missense	97
49.	12140A>G	NSP8	T17A	Missense	5
50.	11288TCTGGTTTT	NSP6	S106	Deletion	72
51.	11296T>G	NSP6	F108L	Missense	14
52.	11224C>T	NSP6	V84V	Silent	9
53.	11288T>A	NSP6	S106T	Missense	6
54.	11291G>A	NSP6	G107S	Missense	6
55.	10449C>A	NSP5	P132H	Missense	100
56.	10447G>A	NSP5	R131R	Silent	100
57.	10198C>T	NSP5	D48D	Silent	95
58.	10029C>T	NSP4	T492I	Missense	100
59.	9344C>T	NSP4	L264F	Missense	99
60.	9866C>T	NSP4	L438F	Missense	99
61.	9424A>G	NSP4	V290V	Silent	98
62.	9534C>T	NSP4	T327I	Missense	97
63.	8937C>T	NSP4	A128V	Missense	2
64.	3037C>T	NSP3	F106F	Silent	99
65.	4184G>A	NSP3	G489S	Missense	99
66.	28344C>T	NSP3	T24I	Missense	99
67.	2911T>C	NSP3	T64T	Silent	99
68.	3927C>T	NSP3	S403L	Missense	99
69.	3796C>T	NSP3	V359V	Silent	99
70.	4321C>T	NSP3	A534A	Silent	98
71.	5183C>T	NSP3	P822S	Missense	98
72.	4586C>T	NSP3	D622D	Silent	76
73.	2710C>T	NSP2	L635L	Silent	3
74.	20055A>G	NSP15	E145E	Silent	99
75.	19955C>T	NSP15	T112I	Missense	99
76.	18163A>G	NSP14	I42V	Missense	99
77.	18583G>A	NSP14	V182I	Missense	24
78.	16887C>T	NSP13	R392C	Missense	99
79.	16887C>T	NSP13	Y217Y	Silent	3
80.	15714C>T	NSP12b	L749L	Silent	100
81.	14408C>T	NSP12b	P314L	Missense	100
82.	15451G>A	NSP12b	G662S	Missense	99
83.	670T>G	NSP1	S135R	Missense	98
84.	28311C>T	N	P13L	Missense	100
85.	28881GGG>AAC	N	RG203KR	Missense	99
86.	28344C>T	N	T24I	Missense	99
87.	29510A>C	N	S413R	Missense	99
88.	28362GAGAACGCA	N	E31	Deletion	72
89.	28363A>T	N	G30G	Silent	10
90.	28370A>G	N	S33G	Missense	6
91.	28367C>T	N	R32C	Missense	2
92.	26709G>A	M	A63T	Missense	99
93.	26858C>T	M	F112F	Silent	99
94.	26577C>G	M	Q19E	Missense	94
95.	26612A>T	M	T30T	Silent	4
96.	26270C>T	E	T9I	Missense	100
97.	26275A>G	E	T11A	Missense	99
98.	241C>T	5’UTR	241	extragenic	77
99.	44C>T	5’UTR	44	extragenic	6
100.	204G>A	5’UTR	204	extragenic	3
101.	28271A>T	3’UTR	28271	extragenic	100
102.	29734GAGGCCACGCGGAGTACGATCGAGTG	3’UTR	29734	extragenic	59
103.	29759G>C	3’UTR	29759	extragenic	6

(Frequency of mutation ≥ 0.02).

**Fig 3:**
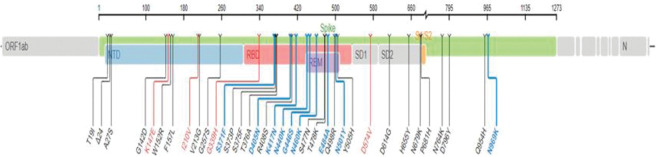
Pictorial view of 36 mutations presents in the different regions of the Spike protein of BA.2.75 Omicron SARS-CoV-2.

**Fig 4:**
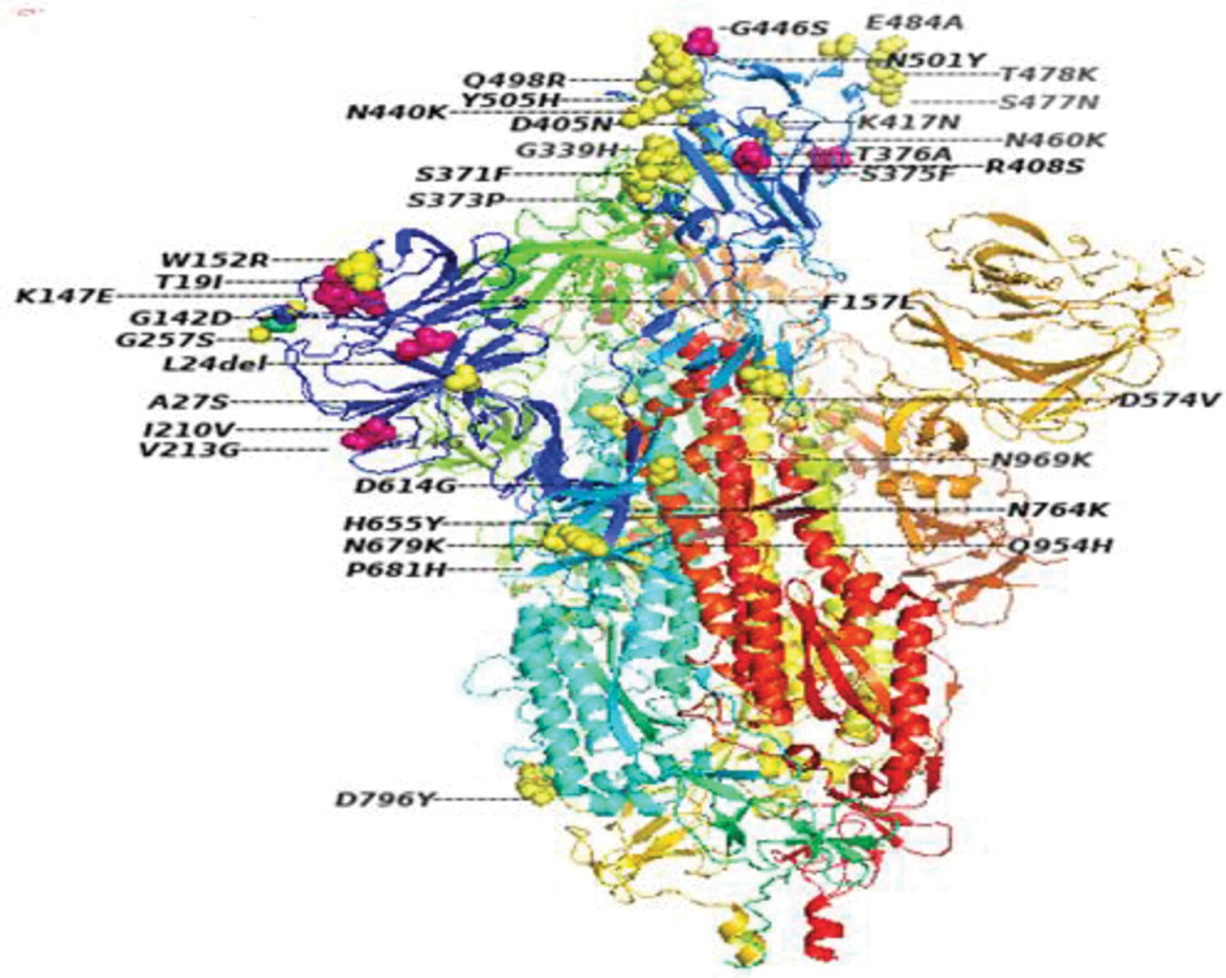
Amino acid residues changed in response to mutations in the Spike protein of BA.2.75 Omicron SARS-CoV-2.

However, among all the proteins of BA.2.75 Omicron SARS-CoV-2, Spike protein had the second highest number of mutations (180 missense, 93 silent SNPs, 3 in-frame deletions, 2 in-frame insertions, 2 frameshift deletions, 4 frameshift insertions and 1 stop-gained variant) after NSP3 (Figs 3 & 4). The occurrence of second largest number of mutations in Spike protein, after NSP3, is in agreement with the mutations pattern of the previously reported variants of SARS-CoV-2 [23, 24]. In the Spike protein, 33 mutations had a prevalence of >80% (Table 2). Out of which three missense mutations including D614G, H655Y, N679K had 100% prevalence while P681H and G339H had 99% and D405N, G257S and N969K had 97% global frequency. Likewise, the frequency of Q954H and D796Y mutations was 96% and that of T19I and N764K was 95% (Table 2).

Additionally, 12 mutations in the Spike proteins including N460K, R408S, G446S, E484A, S477N, N440K, T478K, I210V, V213G, S371F, Y505H and N501Y had 91–94% prevalence (Table 2). And the following 8 mutations Q498R, S373P, K417N, S375F, F157L, W152R, T376A, K147E had an incidence of 81–89%, whereas L24del (78%), G142D (56%), D574V (17%) and L24S, & A27S (6%) had low incidence, comparatively (Table 2). Moreover, all these mutations in the Spike protein were mapped on the SARS-CoV-2 genome (Fig 3).

The comparison of all of the mutations in the Spike protein of BA.2.75 with other sub-variants including BA.1, BA.1.1, BA.2, BA2.12.1, BA.3, BA4 and BA.5 of Omicron SARS-CoV-2 is given in Table 3. Our data revealed that 5 mutations including K147E(81%), W152R(86%), F157L(88%), I210V(92%), and G339H(99%) in the NTD region and G446S(93%) and N460K(94%) in the RBD region of S protein were present with high prevalence in BA2.75 subvariants, whereas these mutations were not reported earlier for any other variant of SAR-CoV-2 (Table 3). Among the other structural proteins T9I(E), P13L(N) and T2231I, T11A(ORF3a) had 100% incidence while T11A(E), A63T (M) and S413R, T24I, RG203KR(N) and D61L(ORF6) had 99% incidence, whereas Q19E(M) and an in-frame deletion E31del(N) had 94% and 72% incidence respectively.

**Table 3:** Comparison of mutations present in the Spike protein of BA.2.75 with other variants of Omicron SARS-CoV-2.

Region	Amino acids in reference genome	Position	BA.2.75	BA.1	BA.1.1	BA.2	BA.2.12.1	BA.3	BA.4	BA.5
**NTD**	T	19	I	-	-	I	-	-	I	I
	L	24	del	-	-	-	-	-	-	-
	A	27	S	-	-	-	-	-	-	-
	G	142	D	D	D	D	D	D	D	D
	K	147	E	-	-	-	-	-	-	-
	W	152	R	-	-	-	-	-	-	-
	F	157	L	-	-	-	-	-	-	-
	I	210	V	-	-	-	-	-	-	-
	V	213	G	-	-	-	G	-	-	-
	G	257	S	-	-	-	-	-	-	-
	G	339	H	D	D	D	D	D	D	D
**RBD**	S	371	F	L	L	F	F	L	F	F
	S	373	P	p	p	p	p	p	p	p
	S	375	F	F	F	F	F	F	F	F
	T	376	A	-	-	A	A	-	A	A
	D	405	N	-	-	N	N	N	N	N
	R	408	S	-	-	S	S	-	S	S
	K	417	N	N	N	N	N	-	N	N
	N	440	K	K	K	K	K	K	K	K
	G	446	S	-	-	-	-	-	-	-
	N	460	K	-	-	-	-	-	-	-
	S	477	N	N	N	N	N	N	N	N
	T	478	K	K	K	K	K	K	K	K
	E	484	A	A	A	A	A	A	A	A
	Q	498	R	R	R	R	R	R	R	R
	N	501	Y	Y	Y	Y	Y	Y	Y	Y
	Y	505	H	H	H	H	H	H	H	H
	D	**574**	V	-	-	-	-	-	-	-
	D	614	G	G	G	G	G	G	G	G
**SD2**	H	655	Y	Y	Y	Y	Y	Y	Y	Y
	N	679	K	K	K	K	K	K	K	K
**S1/S2**	P	**681**	H	H	H	H	H	H	H	H
**ACE2**	**N**	764	K	K	K	K	K	K	K	K
	D	796	Y	Y	Y	Y	Y	Y	Y	Y
**HR1**	Q	954	H	H	H	H	H	H	H	H
	N	969	K	K	K	K	K	K	K	K

### Evolutionary analysis

**Fig 5:**
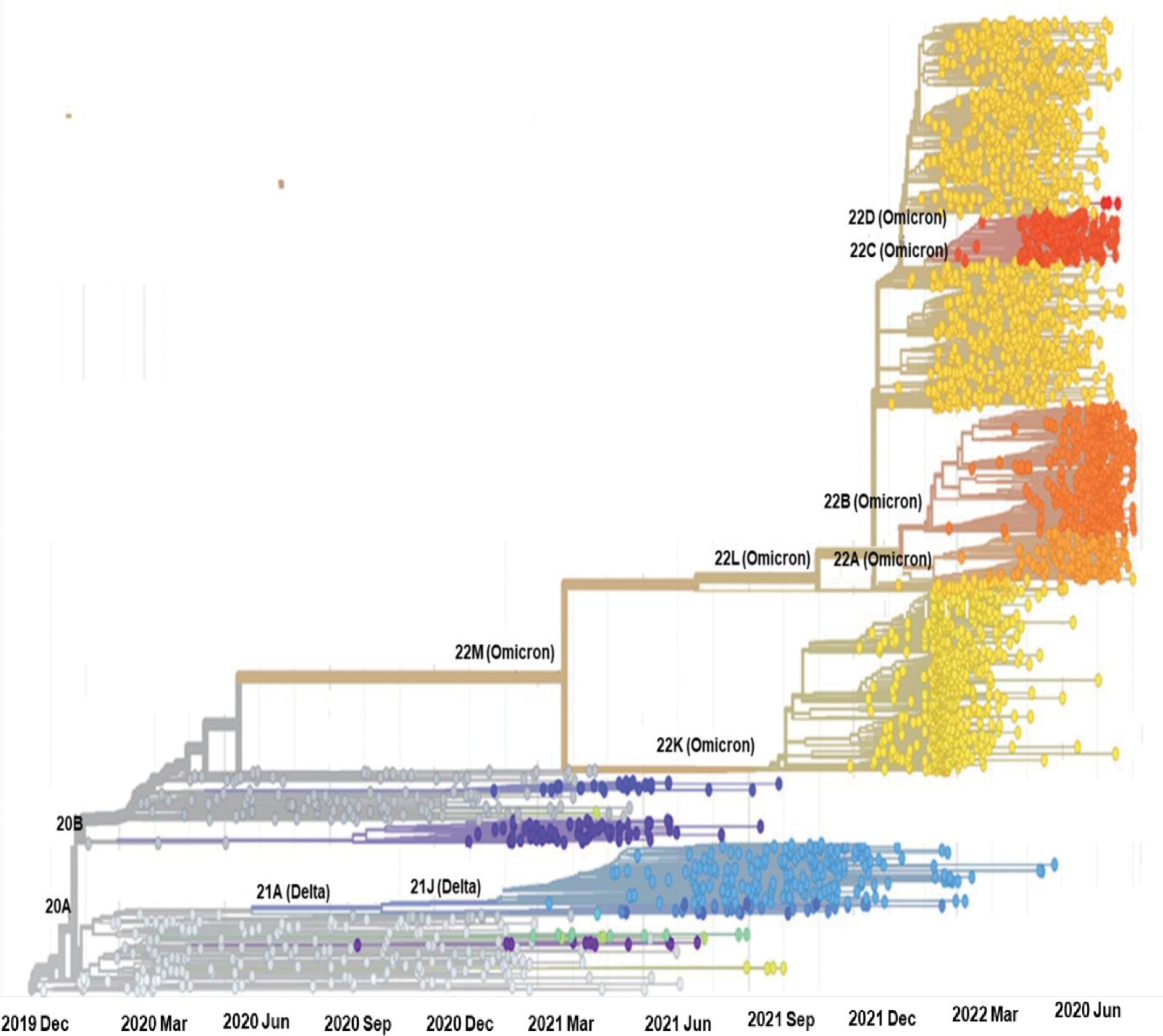
The global phylogeny of SARS-CoV-2 shows the evolutionary relationships of BA.2.75 (22D Omicron) Omicron with other variants of the virus. This phylogenetic tree consisted of 2948 genomes of different variants of SARS-CoV-2 genome collected between December 2019 to August 2022.

For the construction of phylogenomic tree, a set of 2948 genome sequences of different variants of SARS-CoV-2, collected between December 2019 and August 2022, was retrieved from GISAID. Phylogenomic tree was constructed in the Nextstrain (https://nextstrain.org/), which assign clade 22D to the lineage BA2.75, a sub-variant of Omicron SARS-CoV-2. Moreover, it is evident from the phylogeny analysis that BA.2.75 is very close to the lineage BA.2.12.1 (22C clade), a sub-lineage of BA.2 (Fig 5). These two lineages are further clustered with lineages BA.5 and BA.4 which represent the Nextstrain clade 22B, and 22A, respectively (Fig 5).

## Discussion

Here we present the mutations present in the genes/proteins and in the non-coding regions of BA.2.75; a subvariant of Omicron SARS-CoV2. And relate them with the in-vitro activity of authorized MAbs against the variants reported in this study based upon the published literature. In N-terminal domain of the Spike protein, 11 mutations including T19I, L24del, A27S, G142D, K147E, W152R, F157L, I210V, V213G, G257S, and G339H were observed. All of these mutations except T19I, L24del, and G142D and A27S have very high (>80%) prevalence due to which they are most likely to be the characteristic mutations of BA.2.75 and, additionally, they were only observed in BA.2.75 variant of Omicron SARS-CoV-2 (Table 3). However, the T19I (95%) mutations is also present in BA.2, BA.4 and BA.5 while G142D (56%) mutation is present in all other subvariants of Omicron SARS-CoV-2 (Table 3) [15]. Likewise, in the RBD region of the Spike protein, following 17 mutations S371S, S373P, S375F, D405N, K417N, N440K, S477N, T478K, E484A, Q498R, N501Y, Y505H and D574V were detected. Out of which following two missense mutations, G446S and N460K are only present in BA.2.75 subvariant with a frequency of 93% and 94% respectively, and, therefore, are most likely to be the characteristic mutations of this variant. However, determination of the extent to which the mutations in different variants of SARS-CoV-2 can reduce the monoclonal antibodies (MAbs) susceptibility is critical in the prevention and treatment of COVID-19 [25]. The G446S mutation in B.2.75 is reported to be associated in creating the high-level of resistance to imdevimab but not to cilgavimab [26].

In addition, D405N mutation (97% incidence) was also observed in the following Omicron variant BA.2, BA.4, and BA.5 (Table 3), and reported to reduce the susceptibility of these variants to etesevimab (16 to 26-fold) and casirivimab (11 to 14-fold) [3]. Four other missense mutations including N440K, E448A, S477N, and T478K which have 92% prevalence in BA.2.75 Omicron and are also known as the common RBD mutations. It is noteworthy that these mutations have a gradual increase in their prevalence since the start of the pandemic and are still present in all the Omicron SARS-CoV-2 variants (Table 3). Additionally, S371F, N501Y, and Y505H had 91% prevalence in BA.2.75, and are present in the RBD region of Spike protein. The S371F is an RBD core mutation which is also present in the Omicron BA.2, BA.4, and BA.5 variants. It had been reported to drastically reduced the susceptibility of these variants to etesevimab (143 to 630-fold), casirivimab (14 to 28-fold), imdevimab (11 to 126-fold), sotrovimab (5.5 to 21-fold), and tixagevimab (6.3 to 31-fold) [3]. However, the N501Y is present in alpha, beta, gamma, and all variant of Omicron SARS-CoV-2 and reported to mediate the viral entry by enhancing the binding ability of spike protein with ACE-2 receptors [27]. Likewise, S373P, T376A, K417N, and Q498R are present in the RBD region and had 88–89% prevalence in BA.2.75 and are present in all variant of omicron SARS-CoV-2 except K417N which is in RBM mutation and bind with ACE-2 and is also present in Beta, Gamma, and some other Omicron variants.

In addition to RBD mutation, D614G, H655Y and N679K in SD2 region had 100% prevalence while P681H mutation which was located proximal to the S1/S2 furin cleavage site had 99% incidence. The H655Y is involved in increasing the Spike protein cleavage and replication and it is present in Gamma and Omicron variants and many other lineages of SARS-CoV-2. And the D614G, and P681H are the among the preliminary mutations which are present in Alpha, Beta, and each of the Omicron variant [24, 28]. The increase in positive charge associated with this mutation is appeared to influence virus tropism by increasing S1/S2 cleavage in human airway epithelial cells [29]. Moreover, N764K, and D796Y in ACE2 region and Q954H, & N969K in HR1 region have 95–96% and 96–97% prevalence respectively and are known as the most frequent mutations in all the variants of Omicron SARS-CoV-2 [30, 31].

Among the non-structural proteins of BA.2.75 Omicron SARS-CoV-2, T24I, S403L, and G489S, in NSP3; T492I, L264F, L438F in NSP4; P132H in NSP5; P314L, G662S in RdRp protein; R392C in NSP13; I42V in NSP14; and T112I in NSP15 had 99–100% prevalence. However, we suggest G662S in RdRp and S403L in the NSP3 as the characteristic mutations of BA.2.75 Omicron SARS-CoV-2. It is also evident from the fact that their incidence in the global samples of BA.2.75 is 99% and secondly these mutations had not been observed in any other variant of SARS-CoV-2. Moreover, P314L of RdRp had 100% incidence in BA.2.75 and is located very close to the hydrophobic cleft of RdRp which is the target of some antiviral drugs like remdesivir and favipiravir [24, 32]. Hence, the occurrence of highly prevalent mutations in this region of RdRp suggest that this variant is likely to have resistance to various antiviral therapeutic agents. In addition to changing its sensitivity to antiviral drugs, this mutation might be involved in affecting the replication speed of the virus which is the basic function of the RdRp protein [33]. Further to the antiviral treatments, it has been reported that the vaccinated individuals who had suffered with some breakthrough infection from B.1, or B.5 had got protective level of (hybrid) immunity against BA.2.75 compared with those who were only vaccinated (3-doses), which shows high level of cross-immunity between B.1, B.5, and B.2.75 variants [34].

**Fig 6:**
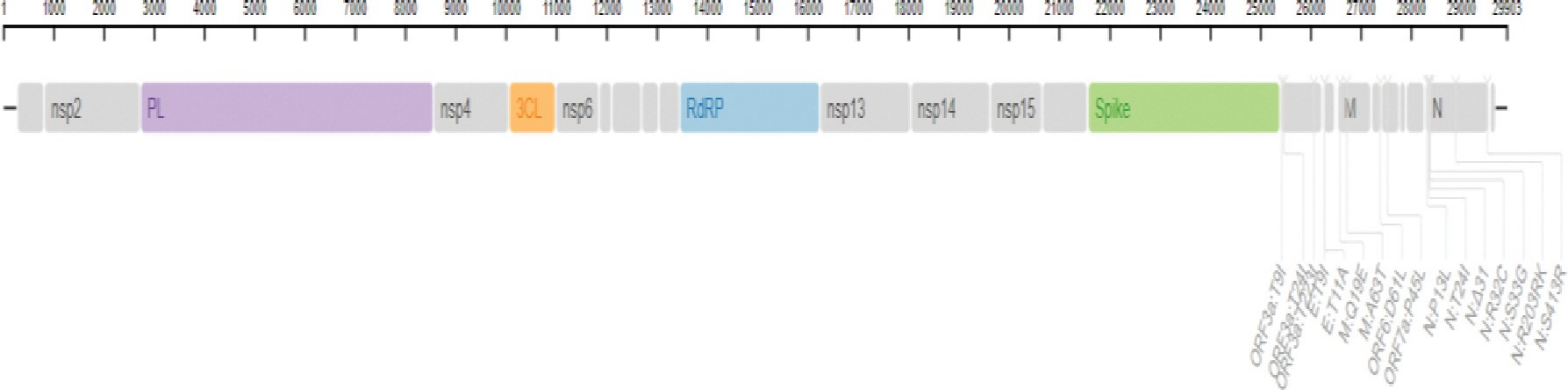
Pictorial view of 15 mutations presents in the structural and accessory proteins, except Spike, of BA.2.75 subvariant of Omicron SARS-CoV-2.

In case of accessory and other structural proteins (Fig 6), the T223I(ORF3a), P13L(N) and T9I(E) are the highly prevalent (100%) mutations in BA.2.75, and are also present in all variants of Omicron SARS-CoV2. Moreover, D614L(ORF6); RG203KR, T24I, S413R (N); and T11A(E) had 99% incidence in all the samples of BA.2.75 Omicron SARS-CoV-2. We also suggest T11A as the characteristic mutation of BA.2.75 of Omicron SARS-CoV2 as it is not observed in any other variant of SARS-CoV-2.

However, phylogeny analysis of the BA.2.75 showed its close relationship with BA.2.12.1, a sub-lineage of BA.2 and our results further revealed that BA.2.75 is descended from the earlier Omicron subvariant, BA.5. This evolutionary relationship of BA.2.75 with BA.5 is key finding in establishing that the surge of BA.5 infections or prior infection with BA.5 may reduce the severity of cases accredited to BA.2.75. This may lead to enhance our knowledge and understanding of the fact that these genetic similarities can prime the immune system to successfully fight-off with one of these subvariant, after defeating an infection from the other.

## Conclusion

We discovered a total of 11 characteristics mutations for BA.2.75, including 9 mutations in the Spike protein, 2 in non-structural proteins, and 1 in the Envelop protein of BA.2.75. Out of these characteristic mutations K147E, W152R, F157L, I210V, V213G, G339H are present in the NTD; G446S and N460K in RBD; S403L in NSP3; G662S in RdRp and T11A in E protein. However, the phylogenetic analysis revealed that BA.2.75 is descended from BA.5, an Omicron sub-variant. This evolutionary link between BA.2.75 and BA.5 is critical in determining whether the surge of BA.5 infection will decrease the severity of cases caused by BA.2.75, suggesting that that the development of immunity and cross-protection between these two variants is also possible. Hence, it is also very likely that a single vaccine may be used to develop the protective level of immunity against both, BA.5 and BA.2.75.

## Supporting information

S1 Table (DOCX)
