# Supplementary material for: Genomic architecture and evolutionary relationship of BA.2.75: A Centaurus subvariant of Omicron SARS-CoV-2
Source: PLoS One. 2023 May 24;18(5):e0281159. doi: 10.1371/journal.pone.0281159 (PMC10208454; doi:10.1371/journal.pone.0281159)
Supplement: S1 Table — (DOCX) [file pone.0281159.s001.docx]

| **Sr. No** | **gisaid_identifier** | **date_collection** | **date_submitted** | **Submitting_lab** |
| --- | --- | --- | --- | --- |
|  | EPI_ISL_13909637 | 7/5/2022 | 7/19/2022 | Helix |
|  | EPI_ISL_13926943 | 6/27/2022 | 7/19/2022 | MVZ Labor Dr. Limbach & Kollegen GbR |
|  | EPI_ISL_13926895 | 6/24/2022 | 7/19/2022 | Labor MÃ¶nchengladbach MVZ Dr. Stein + Kollegen GbR |
|  | EPI_ISL_13929745 | 7/1/2022 | 7/19/2022 | A G Diagnostics Pvt. Ltd, Pune |
|  | EPI_ISL_13929738 | 7/1/2022 | 7/19/2022 | A G Diagnostics Pvt. Ltd, Pune |
|  | EPI_ISL_13929739 | 7/1/2022 | 7/19/2022 | A G Diagnostics Pvt. Ltd, Pune |
|  | EPI_ISL_13929741 | 7/1/2022 | 7/19/2022 | A G Diagnostics Pvt. Ltd, Pune |
|  | EPI_ISL_13931123 | 7/6/2022 | 7/19/2022 | Public Health Ontario Laboratory |
|  | EPI_ISL_13930400 | 7/9/2022 | 7/19/2022 | Public Health Ontario Laboratory |
|  | EPI_ISL_13931316 | 6/29/2022 | 7/19/2022 | Public Health Ontario Laboratory |
|  | EPI_ISL_13931403 | 7/9/2022 | 7/19/2022 | Public Health Ontario Laboratory |
|  | EPI_ISL_13931623 | 7/5/2022 | 7/19/2022 | Public Health Ontario Laboratory |
|  | EPI_ISL_13933623 | 6/26/2022 | 7/19/2022 | amedes MVZ DIAMEDIS Sennestadt |
|  | EPI_ISL_13931760 | 7/5/2022 | 7/19/2022 | Public Health Ontario Laboratory |
|  | EPI_ISL_13932384 | 7/4/2022 | 7/19/2022 | Public Health Ontario Laboratory |
|  | EPI_ISL_13931378 | 7/6/2022 | 7/19/2022 | Public Health Ontario Laboratory |
|  | EPI_ISL_13931757 | 7/5/2022 | 7/19/2022 | Public Health Ontario Laboratory |
|  | EPI_ISL_13930337 | 7/9/2022 | 7/19/2022 | Public Health Ontario Laboratory |
|  | EPI_ISL_13941902 | 6/27/2022 | 7/19/2022 | MVZ Labor Dr. Limbach & Kollegen GbR |
|  | EPI_ISL_13502559 | 6/20/2022 | 6/28/2022 | VRDL GMCH AURANGABAD |
|  | EPI_ISL_13502545 | 6/20/2022 | 6/28/2022 | VRDL GMCH AURANGABAD |
|  | EPI_ISL_13502544 | 6/21/2022 | 6/28/2022 | VRDL GMCH AURANGABAD |
|  | EPI_ISL_13502554 | 6/21/2022 | 6/28/2022 | VRDL GMCH AURANGABAD |
|  | EPI_ISL_13502546 | 6/23/2022 | 6/28/2022 | VRDL GMCH AURANGABAD |
|  | EPI_ISL_13502548 | 6/20/2022 | 6/28/2022 | VRDL GMCH AURANGABAD |
|  | EPI_ISL_13502534 | 6/18/2022 | 6/28/2022 | VRDL GMCH AURANGABAD |
|  | EPI_ISL_13502537 | 6/20/2022 | 6/28/2022 | VRDL GMCH AURANGABAD |
|  | EPI_ISL_13502552 | 6/22/2022 | 6/28/2022 | VRDL GMCH AURANGABAD |
|  | EPI_ISL_13502536 | 6/21/2022 | 6/28/2022 | VRDL GMCH AURANGABAD |
|  | EPI_ISL_13502538 | 6/18/2022 | 6/28/2022 | VRDL GMCH AURANGABAD |
|  | EPI_ISL_13502567 | 6/17/2022 | 6/28/2022 | VRDL GMCH AURANGABAD |
|  | EPI_ISL_13502568 | 6/15/2022 | 6/28/2022 | VRDL GMCH AURANGABAD |
|  | EPI_ISL_13502576 | 6/15/2022 | 6/28/2022 | VRDL GMCH AURANGABAD |
|  | EPI_ISL_13502571 | 6/17/2022 | 6/28/2022 | VRDL GMCH AURANGABAD |
|  | EPI_ISL_13521476 | 6/19/2022 | 6/29/2022 | MAMC Agroha |
|  | EPI_ISL_13949285 | 7/10/2022 | 7/20/2022 | Dhulikhel Hospital, Kathmandu University Hospital |
|  | EPI_ISL_13949288 | 7/10/2022 | 7/20/2022 | Dhulikhel Hospital, Kathmandu University Hospital |
|  | EPI_ISL_13949287 | 7/11/2022 | 7/20/2022 | Dhulikhel Hospital, Kathmandu University Hospital |
|  | EPI_ISL_13949279 | 7/6/2022 | 7/20/2022 | Dhulikhel Hospital, Kathmandu University Hospital |
|  | EPI_ISL_13949278 | 7/6/2022 | 7/20/2022 | Dhulikhel Hospital, Kathmandu University Hospital |
|  | EPI_ISL_13949281 | 7/6/2022 | 7/20/2022 | Dhulikhel Hospital, Kathmandu University Hospital |
|  | EPI_ISL_13949280 | 7/6/2022 | 7/20/2022 | Dhulikhel Hospital, Kathmandu University Hospital |
|  | EPI_ISL_13521477 | 6/20/2022 | 6/29/2022 | MAMC Agroha |
|  | EPI_ISL_13521516 | 6/15/2022 | 6/29/2022 | CRI Kasauli |
|  | EPI_ISL_13521521 | 6/19/2022 | 6/29/2022 | National Institute of Immunology, New Delhi |
|  | EPI_ISL_13521499 | 6/18/2022 | 6/29/2022 | RTPCR Lab Panchkula |
|  | EPI_ISL_13521502 | 6/18/2022 | 6/29/2022 | RTPCR Lab Panchkula |
|  | EPI_ISL_13521486 | 6/7/2022 | 6/29/2022 | NA |
|  | EPI_ISL_13950041 | 6/23/2022 | 7/20/2022 | SARS-CoV-2 testing team, National Institute of Infectious Diseases |
|  | EPI_ISL_13950119 | 6/29/2022 | 7/20/2022 | SARS-CoV-2 testing team, National Institute of Infectious Diseases |
|  | EPI_ISL_13951167 | 6/21/2022 | 7/20/2022 | Regional VRDL ICMR-RMRC, Bhubaneswar |
|  | EPI_ISL_13951149 | 6/21/2022 | 7/20/2022 | Regional VRDL ICMR-RMRC, Bhubaneswar |
|  | EPI_ISL_13502569 | 6/16/2022 | 6/28/2022 | VRDL GMCH AURANGABAD |
|  | EPI_ISL_13502555 | 6/22/2022 | 6/28/2022 | VRDL GMCH AURANGABAD |
|  | EPI_ISL_13502529 | 6/20/2022 | 6/28/2022 | VRDL GMCH AURANGABAD |
|  | EPI_ISL_13502550 | 6/22/2022 | 6/28/2022 | VRDL GMCH AURANGABAD |
|  | EPI_ISL_13952190 | 6/28/2022 | 7/20/2022 | Elling group, Institute of Molecular Biotechnology (IMBA) |
|  | EPI_ISL_13536898 | 6/14/2022 | 6/29/2022 | INSACOG-WB |
|  | EPI_ISL_13536814 | 6/13/2022 | 6/29/2022 | INSACOG-WB |
|  | EPI_ISL_13536822 | 6/13/2022 | 6/29/2022 | INSACOG-WB |
|  | EPI_ISL_13537084 | 6/16/2022 | 6/29/2022 | INSACOG-WB |
|  | EPI_ISL_13537560 | 6/16/2022 | 6/29/2022 | Kasturba Hospital Molecular Lab |
|  | EPI_ISL_13875622 | 7/11/2022 | 7/18/2022 | B34 - Villeneuve |
|  | EPI_ISL_13953664 | 7/7/2022 | 7/20/2022 | Regional VRDL ICMR-RMRC, Bhubaneswar |
|  | EPI_ISL_13953663 | 7/9/2022 | 7/20/2022 | Regional VRDL ICMR-RMRC, Bhubaneswar |
|  | EPI_ISL_13953688 | 7/5/2022 | 7/20/2022 | Regional VRDL ICMR-RMRC, Bhubaneswar |
|  | EPI_ISL_13953686 | 7/6/2022 | 7/20/2022 | Regional VRDL ICMR-RMRC, Bhubaneswar |
|  | EPI_ISL_13953677 | 7/10/2022 | 7/20/2022 | Regional VRDL ICMR-RMRC, Bhubaneswar |
|  | EPI_ISL_13953679 | 7/6/2022 | 7/20/2022 | Regional VRDL ICMR-RMRC, Bhubaneswar |
|  | EPI_ISL_13953670 | 7/9/2022 | 7/20/2022 | Regional VRDL ICMR-RMRC, Bhubaneswar |
|  | EPI_ISL_13953685 | 7/7/2022 | 7/20/2022 | Regional VRDL ICMR-RMRC, Bhubaneswar |
|  | EPI_ISL_13953666 | 7/10/2022 | 7/20/2022 | Regional VRDL ICMR-RMRC, Bhubaneswar |
|  | EPI_ISL_13953689 | 7/10/2022 | 7/20/2022 | Regional VRDL ICMR-RMRC, Bhubaneswar |
|  | EPI_ISL_13953678 | 7/10/2022 | 7/20/2022 | Regional VRDL ICMR-RMRC, Bhubaneswar |
|  | EPI_ISL_13953675 | 7/7/2022 | 7/20/2022 | Regional VRDL ICMR-RMRC, Bhubaneswar |
|  | EPI_ISL_13953673 | 7/7/2022 | 7/20/2022 | Regional VRDL ICMR-RMRC, Bhubaneswar |
|  | EPI_ISL_13953672 | 7/5/2022 | 7/20/2022 | Regional VRDL ICMR-RMRC, Bhubaneswar |
|  | EPI_ISL_13953667 | 7/10/2022 | 7/20/2022 | Regional VRDL ICMR-RMRC, Bhubaneswar |
|  | EPI_ISL_13953668 | 7/10/2022 | 7/20/2022 | Regional VRDL ICMR-RMRC, Bhubaneswar |
|  | EPI_ISL_13954077 | 7/5/2022 | 7/20/2022 | Directorate of Public Health and Preventive Medicine |
|  | EPI_ISL_13954078 | 7/4/2022 | 7/20/2022 | Directorate of Public Health and Preventive Medicine |
|  | EPI_ISL_13954198 | 7/2/2022 | 7/20/2022 | Directorate of Public Health and Preventive Medicine |
|  | EPI_ISL_13954177 | 6/25/2022 | 7/20/2022 | Directorate of Public Health and Preventive Medicine |
|  | EPI_ISL_13954176 | 6/25/2022 | 7/20/2022 | Directorate of Public Health and Preventive Medicine |
|  | EPI_ISL_13954154 | 7/3/2022 | 7/20/2022 | Directorate of Public Health and Preventive Medicine |
|  | EPI_ISL_13954144 | 7/4/2022 | 7/20/2022 | Directorate of Public Health and Preventive Medicine |
|  | EPI_ISL_13954143 | 7/5/2022 | 7/20/2022 | Directorate of Public Health and Preventive Medicine |
|  | EPI_ISL_13955415 | 6/29/2022 | 7/20/2022 | IDSP (KM) |
|  | EPI_ISL_13956776 | 7/7/2022 | 7/20/2022 | Airport Antigen COVIDNet Project |
|  | EPI_ISL_13957683 | 7/5/2022 | 7/20/2022 | Tokyo Metropolitan Institute of Public Health |
|  | EPI_ISL_13957682 | 7/4/2022 | 7/20/2022 | Tokyo Metropolitan Institute of Public Health |
|  | EPI_ISL_13640176 | 6/23/2022 | 7/5/2022 | CSIR-NEERI, Nagpur Covid-19 Testing Lab |
|  | EPI_ISL_13886289 | 7/10/2022 | 7/18/2022 | Clinical Microbiology Laboratory, Tel Aviv Sourasky Medical Center |
|  | EPI_ISL_13962074 | 7/2/2022 | 7/20/2022 | Laboratorio de Referencia Nacional de Virus Inmunoprevenibles. Centro Nacional de Salud Publica. Instituto Nacional de Salud Peru. |
|  | EPI_ISL_13964133 | 7/5/2022 | 7/21/2022 | SARS-CoV-2 testing team, National Institute of Infectious Diseases |
|  | EPI_ISL_13964197 | 7/7/2022 | 7/20/2022 | Tokyo Metropolitan Institute of Public Health |
|  | EPI_ISL_13963101 | 6/23/2022 | 7/21/2022 | British Columbia Centre For Disease Control |
|  | EPI_ISL_13964686 | 6/29/2022 | 7/21/2022 | Pt JLNGMCH Chamba |
|  | EPI_ISL_13964680 | 6/30/2022 | 7/21/2022 | RTPCR Lab Gurugram |
|  | EPI_ISL_13964653 | 7/1/2022 | 7/21/2022 | RTPCR Lab Gurugram |
|  | EPI_ISL_13964690 | 6/17/2022 | 7/21/2022 | RT-PCR Lab, Regional Hospital Una |
|  | EPI_ISL_13964624 | 7/2/2022 | 7/21/2022 | RTPCR Lab Gurugram |
|  | EPI_ISL_13964629 | 7/2/2022 | 7/21/2022 | RTPCR Lab Gurugram |
|  | EPI_ISL_13964667 | 6/28/2022 | 7/21/2022 | RTPCR Lab Gurugram |
|  | EPI_ISL_13964672 | 7/3/2022 | 7/21/2022 | RTPCR Lab Gurugram |
|  | EPI_ISL_13966919 | 7/8/2022 | 7/21/2022 | RTPCR Lab Panchkula |
|  | EPI_ISL_13966934 | 6/26/2022 | 7/21/2022 | IGMC Shimla |
|  | EPI_ISL_13966926 | 7/9/2022 | 7/21/2022 | RTPCR Lab Panchkula |
|  | EPI_ISL_13966927 | 7/9/2022 | 7/21/2022 | RTPCR Lab Panchkula |
|  | EPI_ISL_13966928 | 7/9/2022 | 7/21/2022 | RTPCR Lab Panchkula |
|  | EPI_ISL_13966917 | 7/8/2022 | 7/21/2022 | RTPCR Lab Panchkula |
|  | EPI_ISL_13966920 | 7/8/2022 | 7/21/2022 | RTPCR Lab Panchkula |
|  | EPI_ISL_13966921 | 7/8/2022 | 7/21/2022 | RTPCR Lab Panchkula |
|  | EPI_ISL_13966929 | 7/9/2022 | 7/21/2022 | RTPCR Lab Panchkula |
|  | EPI_ISL_13966946 | 6/29/2022 | 7/21/2022 | IGMC Shimla |
|  | EPI_ISL_13966932 | 6/20/2022 | 7/21/2022 | IGMC Shimla |
|  | EPI_ISL_13966930 | 7/9/2022 | 7/21/2022 | RTPCR Lab Panchkula |
|  | EPI_ISL_13967833 | 6/16/2022 | 7/21/2022 | Supratech Micropath Diagnostics & Research laborat |
|  | EPI_ISL_13967838 | 6/20/2022 | 7/21/2022 | Unipath Speciality Laboaratory, Ahmedabad |
|  | EPI_ISL_13967809 | 6/14/2022 | 7/21/2022 | Pathocare Pathology Laboratory, Vadodara |
|  | EPI_ISL_13967842 | 6/17/2022 | 7/21/2022 | Unipath Speciality Laboaratory, Ahmedabad |
|  | EPI_ISL_13967802 | 7/1/2022 | 7/21/2022 | Pangenomics International Pvt. Ltd, Ahmedabad |
|  | EPI_ISL_13967852 | 6/20/2022 | 7/21/2022 | Zydus Hospital & Healthcare Research Pvt. Ltd. |
|  | EPI_ISL_13967772 | 6/18/2022 | 7/21/2022 | BJ Medical College, Ahmedabad |
|  | EPI_ISL_13968269 | 6/7/2022 | 7/21/2022 | GMERS Medical College, Gandhinagar |
|  | EPI_ISL_13968280 | 7/6/2022 | 7/21/2022 | GMERS Medical College, Gandhinagar |
|  | EPI_ISL_13968267 | 7/7/2022 | 7/21/2022 | GMERS Medical College, Gandhinagar |
|  | EPI_ISL_13968268 | 6/7/2022 | 7/21/2022 | GMERS Medical College, Gandhinagar |
|  | EPI_ISL_13968343 | 6/23/2022 | 7/21/2022 | Supratech Micropath Diagnostics & Research laborat |
|  | EPI_ISL_13968277 | 7/7/2022 | 7/21/2022 | GMERS Medical College, Gandhinagar |
|  | EPI_ISL_13968278 | 7/7/2022 | 7/21/2022 | GMERS Medical College, Gandhinagar |
|  | EPI_ISL_13968276 | 7/7/2022 | 7/21/2022 | GMERS Medical College, Gandhinagar |
|  | EPI_ISL_13968274 | 7/7/2022 | 7/21/2022 | GMERS Medical College, Gandhinagar |
|  | EPI_ISL_13968272 | 7/6/2022 | 7/21/2022 | GMERS Medical College, Gandhinagar |
|  | EPI_ISL_13779649 | 6/23/2022 | 7/12/2022 | INSACOG-WB |
|  | EPI_ISL_13742134 | 6/24/2022 | 7/11/2022 | Respiratory Virus Unit, Microbiology Services Colindale, Public Health England |
|  | EPI_ISL_13969454 | 2022-06 | 7/21/2022 | APOLLO HEALTH AND LIFESTYLE LTD |
|  | EPI_ISL_13969437 | 2022-07 | 7/21/2022 | APOLLO HEALTH AND LIFESTYLE LTD |
|  | EPI_ISL_13969448 | 2022-06 | 7/21/2022 | Neuberg Anand Reference Laboratory, Bengaluru |
|  | EPI_ISL_13969643 | 2022-06 | 7/21/2022 | APOLLO HEALTH AND LIFESTYLE LTD |
|  | EPI_ISL_13969614 | 2022-06 | 7/21/2022 | APOLLO HEALTH AND LIFESTYLE LTD |
|  | EPI_ISL_13969615 | 2022-06 | 7/21/2022 | APOLLO HEALTH AND LIFESTYLE LTD |
|  | EPI_ISL_13969485 | 2022-06 | 7/21/2022 | APOLLO HEALTH AND LIFESTYLE LTD |
|  | EPI_ISL_13969656 | 2022-06 | 7/21/2022 | Neuberg Anand Reference Laboratory, Bengaluru |
|  | EPI_ISL_13969660 | 2022-06 | 7/21/2022 | APOLLO HEALTH AND LIFESTYLE LTD |
|  | EPI_ISL_13969673 | 2022-07 | 7/21/2022 | APOLLO HEALTH AND LIFESTYLE LTD |
|  | EPI_ISL_13969684 | 2022-07 | 7/21/2022 | APOLLO HEALTH AND LIFESTYLE LTD |
|  | EPI_ISL_13969765 | 6/13/2022 | 7/21/2022 | Department of Veterinary Science and Department of Virology I, National Institute of Infectious Diseases |
|  | EPI_ISL_13969835 | 2022-06 | 7/21/2022 | APOLLO HEALTH AND LIFESTYLE LTD |
|  | EPI_ISL_13969859 | 2022-06 | 7/21/2022 | APOLLO HEALTH AND LIFESTYLE LTD |
|  | EPI_ISL_13969778 | 2022-07 | 7/21/2022 | APOLLO HEALTH AND LIFESTYLE LTD |
|  | EPI_ISL_13969802 | 2022-06 | 7/21/2022 | APOLLO HEALTH AND LIFESTYLE LTD |
|  | EPI_ISL_13969868 | 2022-07 | 7/21/2022 | APOLLO HEALTH AND LIFESTYLE LTD |
|  | EPI_ISL_13969874 | 2022-07 | 7/21/2022 | APOLLO HEALTH AND LIFESTYLE LTD |
|  | EPI_ISL_13969947 | 2022-07 | 7/21/2022 | APOLLO HEALTH AND LIFESTYLE LTD |
|  | EPI_ISL_13969953 | 2022-06 | 7/21/2022 | APOLLO HEALTH AND LIFESTYLE LTD |
|  | EPI_ISL_13969945 | 2022-06 | 7/21/2022 | APOLLO HEALTH AND LIFESTYLE LTD |
|  | EPI_ISL_13969956 | 2022-07 | 7/21/2022 | APOLLO HEALTH AND LIFESTYLE LTD |
|  | EPI_ISL_13969841 | 2022-07 | 7/21/2022 | APOLLO HEALTH AND LIFESTYLE LTD |
|  | EPI_ISL_13969843 | 2022-07 | 7/21/2022 | APOLLO HEALTH AND LIFESTYLE LTD |
|  | EPI_ISL_13970110 | 2022-07 | 7/21/2022 | APOLLO HEALTH AND LIFESTYLE LTD |
|  | EPI_ISL_13970137 | 2022-07 | 7/21/2022 | APOLLO HEALTH AND LIFESTYLE LTD |
|  | EPI_ISL_13970042 | 2022-06 | 7/21/2022 | APOLLO HEALTH AND LIFESTYLE LTD |
|  | EPI_ISL_13970050 | 2022-07 | 7/21/2022 | APOLLO HEALTH AND LIFESTYLE LTD |
|  | EPI_ISL_13969993 | 2022-07 | 7/21/2022 | APOLLO HEALTH AND LIFESTYLE LTD |
|  | EPI_ISL_13970080 | 2022-06 | 7/21/2022 | APOLLO HEALTH AND LIFESTYLE LTD |
|  | EPI_ISL_13970093 | 2022-07 | 7/21/2022 | APOLLO HEALTH AND LIFESTYLE LTD |
|  | EPI_ISL_13971518 | 7/14/2022 | 7/21/2022 | Department of Bacteria, Parasites and Fungi, Statens Serum Institut, Copenhagen, Denmark |
|  | EPI_ISL_13971647 | 7/15/2022 | 7/21/2022 | Department of Bacteria, Parasites and Fungi, Statens Serum Institut, Copenhagen, Denmark |
|  | EPI_ISL_13971636 | 7/12/2022 | 7/21/2022 | Department of Bacteria, Parasites and Fungi, Statens Serum Institut, Copenhagen, Denmark |
|  | EPI_ISL_13971879 | 7/8/2022 | 7/21/2022 | Department of Bacteria, Parasites and Fungi, Statens Serum Institut, Copenhagen, Denmark |
|  | EPI_ISL_13977567 | 7/5/2022 | 7/21/2022 | Laboratory Corporation of America |
|  | EPI_ISL_13978609 | 7/9/2022 | 7/21/2022 | Laboratory Corporation of America |
|  | EPI_ISL_13981279 | 7/6/2022 | 7/21/2022 | B J Government Medical College and Sassoon General Hospitals, Pune |
|  | EPI_ISL_13981280 | 7/9/2022 | 7/21/2022 | B J Government Medical College and Sassoon General Hospitals, Pune |
|  | EPI_ISL_13981301 | 7/9/2022 | 7/21/2022 | B J Government Medical College and Sassoon General Hospitals, Pune |
|  | EPI_ISL_13981298 | 7/7/2022 | 7/21/2022 | B J Government Medical College and Sassoon General Hospitals, Pune |
|  | EPI_ISL_13981624 | 7/3/2022 | 7/21/2022 | UW Virology Lab |
|  | EPI_ISL_13985762 | 7/10/2022 | 7/22/2022 | Tokyo Metropolitan Institute of Public Health |
|  | EPI_ISL_13985836 | 7/7/2022 | 7/22/2022 | Division of Emerging Infectious Diseases, Bureau of Infectious Diseases Diagnosis Control, Korea Disease Control and Prevention Agency |
|  | EPI_ISL_13985838 | 7/19/2022 | 7/22/2022 | Division of Emerging Infectious Diseases, Bureau of Infectious Diseases Diagnosis Control, Korea Disease Control and Prevention Agency |
|  | EPI_ISL_13985837 | 7/11/2022 | 7/22/2022 | Division of Emerging Infectious Diseases, Bureau of Infectious Diseases Diagnosis Control, Korea Disease Control and Prevention Agency |
|  | EPI_ISL_13574753 | 6/9/2022 | 7/1/2022 | AIIMS Bhopal VRDL/Translational Medicine - INSACOG |
|  | EPI_ISL_13988951 | 6/28/2022 | 7/22/2022 | Gandhi Medical College and Hospital (GMCH), Secunderabad |
|  | EPI_ISL_13989118 | 6/10/2022 | 7/22/2022 | ICMR-National Institute of Virology - INSACOG |
|  | EPI_ISL_13989970 | 7/4/2022 | 7/22/2022 | Unipeth Pathology Laboratory -Vadodara |
|  | EPI_ISL_13989997 | 7/7/2022 | 7/22/2022 | Pangenomics International Pvt. Ltd, Ahmedabad |
|  | EPI_ISL_13989991 | 7/1/2022 | 7/22/2022 | Supratech Micropath Diagnostics & Research laborat |
|  | EPI_ISL_13989998 | 7/15/2022 | 7/22/2022 | Pangenomics International Pvt. Ltd, Ahmedabad |
|  | EPI_ISL_13989994 | 6/30/2022 | 7/22/2022 | Speciality Micro tech lab |
|  | EPI_ISL_13990006 | 7/14/2022 | 7/22/2022 | GMC, Gandhinagar |
|  | EPI_ISL_13990009 | 7/3/2022 | 7/22/2022 | General Hospital, Mehsana |
|  | EPI_ISL_13795649 | 7/8/2022 | 7/13/2022 | Department of Bacteria, Parasites and Fungi, Statens Serum Institut, Copenhagen, Denmark |
|  | EPI_ISL_13999079 | 6/16/2022 | 7/22/2022 | Edmonton Provincial Lab |
|  | EPI_ISL_13999627 | 6/30/2022 | 7/22/2022 | Edmonton Provincial Lab |
|  | EPI_ISL_13999281 | 6/24/2022 | 7/22/2022 | Edmonton Provincial Lab |
|  | EPI_ISL_13999625 | 6/30/2022 | 7/22/2022 | Edmonton Provincial Lab |
|  | EPI_ISL_13803882 | 6/23/2022 | 7/13/2022 | Directorate of Public Health and Preventive Medicine |
|  | EPI_ISL_13803881 | 6/23/2022 | 7/13/2022 | Directorate of Public Health and Preventive Medicine |
|  | EPI_ISL_13803883 | 6/23/2022 | 7/13/2022 | Directorate of Public Health and Preventive Medicine |
|  | EPI_ISL_13803967 | 6/20/2022 | 7/13/2022 | Directorate of Public Health and Preventive Medicine |
|  | EPI_ISL_13804325 | 1/7/2022 | 7/13/2022 | Christian Medical College |
|  | EPI_ISL_13804386 | 6/16/2022 | 7/13/2022 | CRL, KIMS |
|  | EPI_ISL_14002547 | 7/1/2022 | 7/22/2022 | Quest Diagnostics Incorporated |
|  | EPI_ISL_14004434 | 7/13/2022 | 7/23/2022 | RS Tzu Chi Pantai Indah Kapuk |
|  | EPI_ISL_14004943 | 7/1/2022 | 7/23/2022 | Directorate of Public Health and Preventive Medicine |
|  | EPI_ISL_14004944 | 7/1/2022 | 7/23/2022 | Directorate of Public Health and Preventive Medicine |
|  | EPI_ISL_14005000 | 7/2/2022 | 7/23/2022 | Directorate of Public Health and Preventive Medicine |
|  | EPI_ISL_14005128 | 7/12/2022 | 7/23/2022 | RSAB Harapan Kita |
|  | EPI_ISL_14005271 | 7/2/2022 | 7/23/2022 | Directorate of Public Health and Preventive Medicine |
|  | EPI_ISL_14005273 | 7/2/2022 | 7/23/2022 | Directorate of Public Health and Preventive Medicine |
|  | EPI_ISL_14009553 | 7/10/2022 | 7/23/2022 | Laboratory Corporation of America |
|  | EPI_ISL_14010188 | 7/13/2022 | 7/22/2022 | Arizona State University |
|  | EPI_ISL_14011208 | 7/15/2022 | 7/23/2022 | Carolinas HealthCare Systems Laboratory |
|  | EPI_ISL_13610938 | 2022-06 | 7/3/2022 | Neuberg Anand Reference Laboratory, Bengaluru |
|  | EPI_ISL_13611168 | 2022-06 | 7/3/2022 | Neuberg Anand Reference Laboratory, Bengaluru |
|  | EPI_ISL_13610928 | 2022-06 | 7/3/2022 | Neuberg Anand Reference Laboratory, Bengaluru |
|  | EPI_ISL_13610920 | 2022-06 | 7/3/2022 | Neuberg Anand Reference Laboratory, Bengaluru |
|  | EPI_ISL_14011362 | 6/13/2022 | 7/24/2022 | Division of Virology, Institute of Medical Science, The University of Tokyo |
|  | EPI_ISL_14011506 | 7/15/2022 | 7/23/2022 | RS Tzu Chi Pantai Indah Kapuk |
|  | EPI_ISL_13623453 | 6/21/2022 | 7/4/2022 | Public Health Ontario Laboratory |
|  | EPI_ISL_14012010 | 7/13/2022 | 7/24/2022 | Department of Bacteria, Parasites and Fungi, Statens Serum Institut, Copenhagen, Denmark |
|  | EPI_ISL_13634399 | 6/26/2022 | 7/5/2022 | Rosalind Franklin Laboratory |
|  | EPI_ISL_13634479 | 6/25/2022 | 7/5/2022 | Rosalind Franklin Laboratory |
|  | EPI_ISL_13640138 | 6/24/2022 | 7/5/2022 | CSIR-NEERI, Nagpur Covid-19 Testing Lab |
|  | EPI_ISL_13640142 | 6/22/2022 | 7/5/2022 | CSIR-NEERI, Nagpur Covid-19 Testing Lab |
|  | EPI_ISL_13640184 | 6/26/2022 | 7/5/2022 | CSIR-NEERI, Nagpur Covid-19 Testing Lab |
|  | EPI_ISL_13640175 | 6/24/2022 | 7/5/2022 | CSIR-NEERI, Nagpur Covid-19 Testing Lab |
|  | EPI_ISL_13640162 | 6/24/2022 | 7/5/2022 | CSIR-NEERI, Nagpur Covid-19 Testing Lab |
|  | EPI_ISL_13640161 | 6/20/2022 | 7/5/2022 | CSIR-NEERI, Nagpur Covid-19 Testing Lab |
|  | EPI_ISL_13640129 | 6/20/2022 | 7/5/2022 | CSIR-NEERI, Nagpur Covid-19 Testing Lab |
|  | EPI_ISL_13640137 | 6/26/2022 | 7/5/2022 | CSIR-NEERI, Nagpur Covid-19 Testing Lab |
|  | EPI_ISL_13640194 | 6/27/2022 | 7/5/2022 | CSIR-NEERI, Nagpur Covid-19 Testing Lab |
|  | EPI_ISL_13640189 | 6/22/2022 | 7/5/2022 | CSIR-NEERI, Nagpur Covid-19 Testing Lab |
|  | EPI_ISL_14015181 | 7/14/2022 | 7/24/2022 | Microbiological Diagnostic Unit - Public Health Laboratory (MDU-PHL) |
|  | EPI_ISL_13657191 | 6/21/2022 | 7/6/2022 | RSPTN UNUD |
|  | EPI_ISL_13655926 | 6/28/2022 | 7/6/2022 | Rosalind Franklin Laboratory |
|  | EPI_ISL_13822874 | 6/18/2022 | 7/14/2022 | COVID-CBNAAT; Department of Microbiology; AIIMS; New Delhi |
|  | EPI_ISL_14018142 | 7/2/2022 | 7/25/2022 | IDSP, Kamrup Metro (SRL) |
|  | EPI_ISL_14018144 | 7/2/2022 | 7/25/2022 | IDSP, Kamrup Metro (SRL) |
|  | EPI_ISL_14018143 | 7/2/2022 | 7/25/2022 | IDSP, Kamrup Metro (SRL) |
|  | EPI_ISL_14018380 | 7/6/2022 | 7/25/2022 | Directorate of Public Health and Preventive Medicine |
|  | EPI_ISL_13826301 | 6/15/2022 | 7/14/2022 | COVID-CBNAAT; Department of Microbiology; AIIMS; New Delhi |
|  | EPI_ISL_13826307 | 6/16/2022 | 7/14/2022 | COVID-CBNAAT; Department of Microbiology; AIIMS; New Delhi |
|  | EPI_ISL_13826295 | 6/14/2022 | 7/14/2022 | COVID-CBNAAT; Department of Microbiology; AIIMS; New Delhi |
|  | EPI_ISL_14023393 | 7/9/2022 | 7/25/2022 | Respiratory Virus Unit, Microbiology Services Colindale, Public Health England |
|  | EPI_ISL_14023858 | 7/9/2022 | 7/25/2022 | Respiratory Virus Unit, Microbiology Services Colindale, Public Health England |
|  | EPI_ISL_14024273 | 7/12/2022 | 7/25/2022 | Respiratory Virus Unit, Microbiology Services Colindale, Public Health England |
|  | EPI_ISL_13848108 | 6/19/2022 | 7/15/2022 | SMS MEDICAL COLLEGE,JAIPUR |
|  | EPI_ISL_13848126 | 6/28/2022 | 7/15/2022 | SMS MEDICAL COLLEGE,JAIPUR |
|  | EPI_ISL_13841680 | 6/27/2022 | 7/15/2022 | Respiratory Virus Unit, Microbiology Services Colindale, Public Health England |
|  | EPI_ISL_13848162 | 6/26/2022 | 7/15/2022 | SMS MEDICAL COLLEGE,JAIPUR |
|  | EPI_ISL_13848180 | 6/28/2022 | 7/15/2022 | SMS MEDICAL COLLEGE,JAIPUR |
|  | EPI_ISL_13848061 | 6/20/2022 | 7/15/2022 | SMS MEDICAL COLLEGE,JAIPUR |
|  | EPI_ISL_13847294 | 7/4/2022 | 7/15/2022 | Gandhi Medical College and Hospital (GMCH), Secunderabad |
|  | EPI_ISL_13848089 | 6/21/2022 | 7/15/2022 | SMS MEDICAL COLLEGE,JAIPUR |
|  | EPI_ISL_13847979 | 6/21/2022 | 7/15/2022 | SMS MEDICAL COLLEGE,JAIPUR |
|  | EPI_ISL_13848078 | 6/21/2022 | 7/15/2022 | SMS MEDICAL COLLEGE,JAIPUR |
|  | EPI_ISL_14027055 | 7/7/2022 | 7/25/2022 | Directorate of Public Health and Preventive Medicine |
|  | EPI_ISL_14027125 | 7/4/2022 | 7/25/2022 | Scientific Diagnostic Center Pvt.Ltd, Ahmedabad |
|  | EPI_ISL_14027138 | 7/3/2022 | 7/25/2022 | Pangenomics International Pvt. Ltd, Ahmedabad |
|  | EPI_ISL_14027149 | 6/27/2022 | 7/25/2022 | M.P.Shah Government Medical College, Jamnagar |
|  | EPI_ISL_14027146 | 7/1/2022 | 7/25/2022 | Government Medical College, Bhavnagar |
|  | EPI_ISL_14027159 | 6/30/2022 | 7/25/2022 | Toprani Advance lab Systems, Vadodara |
|  | EPI_ISL_14027144 | 7/2/2022 | 7/25/2022 | Sterling Diagnostic Center, Bhavnagar |
|  | EPI_ISL_14027161 | 7/2/2022 | 7/25/2022 | Toprani Advance lab Systems, Vadodara |
|  | EPI_ISL_14027145 | 7/2/2022 | 7/25/2022 | Government Medical College, Bhavnagar |
|  | EPI_ISL_14027154 | 7/5/2022 | 7/25/2022 | General Hospital, Surendranagar |
|  | EPI_ISL_13848889 | 7/2/2022 | 7/15/2022 | Helix |
|  | EPI_ISL_13856159 | 7/3/2022 | 7/15/2022 | UW Virology Lab |
|  | EPI_ISL_13856119 | 7/4/2022 | 7/15/2022 | UW Virology Lab |
|  | EPI_ISL_13864531 | 7/10/2022 | 7/16/2022 | Dr. Yoshitaka Tamura Department of Clinical Laboratory, Osaka Habikino Medical Center |
|  | EPI_ISL_13864544 | 7/12/2022 | 7/16/2022 | Dr. Yoshitaka Tamura Department of Clinical Laboratory, Osaka Habikino Medical Center |
|  | EPI_ISL_13869588 | 7/2/2022 | 7/17/2022 | Douglass Hanly Moir Pathology |
|  | EPI_ISL_13871117 | 7/12/2022 | 7/18/2022 | Microbiological Diagnostic Unit - Public Health Laboratory (MDU-PHL) |
|  | EPI_ISL_13870910 | 7/11/2022 | 7/18/2022 | Microbiological Diagnostic Unit - Public Health Laboratory (MDU-PHL) |
|  | EPI_ISL_13870525 | 7/3/2022 | 7/18/2022 | Microbiological Diagnostic Unit - Public Health Laboratory (MDU-PHL) |
|  | EPI_ISL_14036906 | 7/13/2022 | 7/25/2022 | Public Health Ontario Laboratory |
|  | EPI_ISL_14037014 | 7/13/2022 | 7/25/2022 | Public Health Ontario Laboratory |
|  | EPI_ISL_14037256 | 7/13/2022 | 7/25/2022 | Public Health Ontario Laboratory |
|  | EPI_ISL_14037367 | 7/16/2022 | 7/25/2022 | Public Health Ontario Laboratory |
|  | EPI_ISL_14037150 | 7/13/2022 | 7/25/2022 | Public Health Ontario Laboratory |
|  | EPI_ISL_14037761 | 7/14/2022 | 7/25/2022 | Public Health Ontario Laboratory |
|  | EPI_ISL_13876890 | 6/23/2022 | 7/18/2022 | INSACOG-WB |
|  | EPI_ISL_13876811 | 6/14/2022 | 7/18/2022 | INSACOG-WB |
|  | EPI_ISL_13876812 | 6/14/2022 | 7/18/2022 | INSACOG-WB |
|  | EPI_ISL_13876837 | 6/19/2022 | 7/18/2022 | INSACOG-WB |
|  | EPI_ISL_13876838 | 6/19/2022 | 7/18/2022 | INSACOG-WB |
|  | EPI_ISL_13876835 | 6/23/2022 | 7/18/2022 | INSACOG-WB |
|  | EPI_ISL_13876841 | 6/19/2022 | 7/18/2022 | INSACOG-WB |
|  | EPI_ISL_13876794 | 6/24/2022 | 7/18/2022 | INSACOG-WB |
|  | EPI_ISL_13876830 | 6/18/2022 | 7/18/2022 | INSACOG-WB |
|  | EPI_ISL_13877235 | 6/30/2022 | 7/18/2022 | INSACOG-WB |
|  | EPI_ISL_13877231 | 6/30/2022 | 7/18/2022 | INSACOG-WB |
|  | EPI_ISL_13877254 | 6/23/2022 | 7/18/2022 | INSACOG-WB |
|  | EPI_ISL_13877250 | 6/22/2022 | 7/18/2022 | INSACOG-WB |
|  | EPI_ISL_13877252 | 6/23/2022 | 7/18/2022 | INSACOG-WB |
|  | EPI_ISL_13876922 | 6/24/2022 | 7/18/2022 | INSACOG-WB |
|  | EPI_ISL_13877102 | 6/28/2022 | 7/18/2022 | INSACOG-WB |
|  | EPI_ISL_13876907 | 6/24/2022 | 7/18/2022 | INSACOG-WB |
|  | EPI_ISL_13877144 | 6/29/2022 | 7/18/2022 | INSACOG-WB |
|  | EPI_ISL_13877152 | 6/29/2022 | 7/18/2022 | INSACOG-WB |
|  | EPI_ISL_13876966 | 6/25/2022 | 7/18/2022 | INSACOG-WB |
|  | EPI_ISL_13877143 | 6/29/2022 | 7/18/2022 | INSACOG-WB |
|  | EPI_ISL_13877140 | 6/29/2022 | 7/18/2022 | INSACOG-WB |
|  | EPI_ISL_13876956 | 6/25/2022 | 7/18/2022 | INSACOG-WB |
|  | EPI_ISL_13877141 | 6/29/2022 | 7/18/2022 | INSACOG-WB |
|  | EPI_ISL_13877135 | 6/29/2022 | 7/18/2022 | INSACOG-WB |
|  | EPI_ISL_13877136 | 6/29/2022 | 7/18/2022 | INSACOG-WB |
|  | EPI_ISL_13876948 | 6/25/2022 | 7/18/2022 | INSACOG-WB |
|  | EPI_ISL_13877072 | 6/28/2022 | 7/18/2022 | INSACOG-WB |
|  | EPI_ISL_13876993 | 6/26/2022 | 7/18/2022 | INSACOG-WB |
|  | EPI_ISL_13876996 | 6/27/2022 | 7/18/2022 | INSACOG-WB |
|  | EPI_ISL_13877092 | 6/28/2022 | 7/18/2022 | INSACOG-WB |
|  | EPI_ISL_13877225 | 6/30/2022 | 7/18/2022 | INSACOG-WB |
|  | EPI_ISL_13877181 | 6/30/2022 | 7/18/2022 | INSACOG-WB |
|  | EPI_ISL_13877175 | 6/29/2022 | 7/18/2022 | INSACOG-WB |
|  | EPI_ISL_13877245 | 6/17/2022 | 7/18/2022 | INSACOG-WB |
|  | EPI_ISL_13877208 | 6/30/2022 | 7/18/2022 | INSACOG-WB |
|  | EPI_ISL_13877202 | 6/30/2022 | 7/18/2022 | INSACOG-WB |
|  | EPI_ISL_13877247 | 6/20/2022 | 7/18/2022 | INSACOG-WB |
|  | EPI_ISL_13877248 | 6/21/2022 | 7/18/2022 | INSACOG-WB |
|  | EPI_ISL_13882158 | 7/2/2022 | 7/18/2022 | Respiratory Virus Unit, Microbiology Services Colindale, Public Health England |
|  | EPI_ISL_13877323 | 7/13/2022 | 7/18/2022 | Fondazione IRCCS Ca' Granda Ospedale Maggiore Policlinico |
|  | EPI_ISL_13692007 | 6/21/2022 | 7/7/2022 | Laboratorium Pathlab Jakarta Utara |
|  | EPI_ISL_13884499 | 6/28/2022 | 7/18/2022 | Regional Medical Sciences Center 12/1 Trang |
|  | EPI_ISL_13964685 | 6/28/2022 | 7/21/2022 | Pt JLNGMCH Chamba |
|  | EPI_ISL_13969709 | 2022-07 | 7/21/2022 | APOLLO HEALTH AND LIFESTYLE LTD |
|  | EPI_ISL_14049583 | 7/5/2022 | 7/26/2022 | National Public Health Laboratory, National Centre for Infectious Diseases |
|  | EPI_ISL_14049586 | 7/15/2022 | 7/26/2022 | National Public Health Laboratory, National Centre for Infectious Diseases |
|  | EPI_ISL_14049577 | 7/12/2022 | 7/26/2022 | National Public Health Laboratory, National Centre for Infectious Diseases |
|  | EPI_ISL_13966941 | 7/2/2022 | 7/21/2022 | IGMC Shimla |
|  | EPI_ISL_13969913 | 2022-06 | 7/21/2022 | APOLLO HEALTH AND LIFESTYLE LTD |
|  | EPI_ISL_13969811 | 2022-07 | 7/21/2022 | APOLLO HEALTH AND LIFESTYLE LTD |
|  | EPI_ISL_13964689 | 6/29/2022 | 7/21/2022 | Pt JLNGMCH Chamba |
|  | EPI_ISL_13953683 | 7/6/2022 | 7/20/2022 | Regional VRDL ICMR-RMRC, Bhubaneswar |
|  | EPI_ISL_13949277 | 7/6/2022 | 7/20/2022 | Dhulikhel Hospital, Kathmandu University Hospital |
|  | EPI_ISL_14052488 | 7/5/2022 | 7/26/2022 | Laboratory Corporation of America |
|  | EPI_ISL_13302209 | 6/7/2022 | 6/15/2022 | CSIR-NEERI, Nagpur Covid-19 Testing Lab |
|  | EPI_ISL_14055817 | 7/13/2022 | 7/26/2022 | Laboratory Corporation of America |
|  | EPI_ISL_14054623 | 7/11/2022 | 7/26/2022 | Laboratory Corporation of America |
|  | EPI_ISL_14054684 | 7/11/2022 | 7/26/2022 | Laboratory Corporation of America |
|  | EPI_ISL_14053908 | 7/9/2022 | 7/26/2022 | Laboratory Corporation of America |
|  | EPI_ISL_14052542 | 7/5/2022 | 7/26/2022 | Laboratory Corporation of America |
|  | EPI_ISL_14052405 | 7/5/2022 | 7/26/2022 | Laboratory Corporation of America |
|  | EPI_ISL_14049580 | 7/12/2022 | 7/26/2022 | National Public Health Laboratory, National Centre for Infectious Diseases |
|  | EPI_ISL_14049587 | 7/14/2022 | 7/26/2022 | National Public Health Laboratory, National Centre for Infectious Diseases |
|  | EPI_ISL_14049585 | 7/15/2022 | 7/26/2022 | National Public Health Laboratory, National Centre for Infectious Diseases |
|  | EPI_ISL_14049584 | 7/14/2022 | 7/26/2022 | National Public Health Laboratory, National Centre for Infectious Diseases |
|  | EPI_ISL_14049581 | 7/11/2022 | 7/26/2022 | National Public Health Laboratory, National Centre for Infectious Diseases |
|  | EPI_ISL_14049582 | 7/4/2022 | 7/26/2022 | National Public Health Laboratory, National Centre for Infectious Diseases |
|  | EPI_ISL_14049578 | 7/12/2022 | 7/26/2022 | National Public Health Laboratory, National Centre for Infectious Diseases |
|  | EPI_ISL_14049579 | 7/12/2022 | 7/26/2022 | National Public Health Laboratory, National Centre for Infectious Diseases |
|  | EPI_ISL_14049576 | 7/12/2022 | 7/26/2022 | National Public Health Laboratory, National Centre for Infectious Diseases |
|  | EPI_ISL_14047124 | 7/18/2022 | 7/26/2022 | Department of Bacteria, Parasites and Fungi, Statens Serum Institut, Copenhagen, Denmark |
|  | EPI_ISL_14047346 | 7/5/2022 | 7/26/2022 | Institute of Microbiology and Immunology, Faculty of Medicine, University of Ljubljana |
|  | EPI_ISL_14046399 | 7/17/2022 | 7/26/2022 | Department of Bacteria, Parasites and Fungi, Statens Serum Institut, Copenhagen, Denmark |
|  | EPI_ISL_14043552 | 7/5/2022 | 7/26/2022 | Rosalind Franklin Laboratory |
|  | EPI_ISL_14041543 | 7/7/2022 | 7/26/2022 | Rosalind Franklin Laboratory |
|  | EPI_ISL_13520025 | 2022-06 | 6/29/2022 | APOLLO HEALTH AND LIFESTYLE LTD |
|  | EPI_ISL_14059442 | 7/5/2022 | 7/26/2022 | Kaiser Permanente Southern California |
|  | EPI_ISL_13521515 | 6/15/2022 | 6/29/2022 | CRI Kasauli |
|  | EPI_ISL_13521496 | 6/17/2022 | 6/29/2022 | RTPCR Lab Panchkula |
|  | EPI_ISL_14060732 | 7/6/2022 | 7/26/2022 | Quest Diagnostics Incorporated |
|  | EPI_ISL_14061038 | 7/6/2022 | 7/26/2022 | Quest Diagnostics Incorporated |
|  | EPI_ISL_13536654 | 6/8/2022 | 6/29/2022 | INSACOG-WB |
|  | EPI_ISL_13536794 | 6/12/2022 | 6/29/2022 | INSACOG-WB |
|  | EPI_ISL_13536799 | 6/13/2022 | 6/29/2022 | INSACOG-WB |
|  | EPI_ISL_14069001 | 7/19/2022 | 7/27/2022 | Department of Bacteria, Parasites and Fungi, Statens Serum Institut, Copenhagen, Denmark |
|  | EPI_ISL_14068866 | 7/20/2022 | 7/27/2022 | Department of Bacteria, Parasites and Fungi, Statens Serum Institut, Copenhagen, Denmark |
|  | EPI_ISL_14069548 | 7/20/2022 | 7/27/2022 | Department of Bacteria, Parasites and Fungi, Statens Serum Institut, Copenhagen, Denmark |
|  | EPI_ISL_13887552 | 7/9/2022 | 7/18/2022 | Pandemic Response Lab - NYC |
|  | EPI_ISL_13892269 | 2022 | 7/18/2022 | MRU LAB, IMS BHU |
|  | EPI_ISL_13892311 | 2022 | 7/18/2022 | MRU LAB, IMS BHU |
|  | EPI_ISL_13892350 | 2022 | 7/18/2022 | MRU LAB, IMS BHU |
|  | EPI_ISL_13892302 | 2022 | 7/18/2022 | MRU LAB, IMS BHU |
|  | EPI_ISL_13892272 | 2022 | 7/18/2022 | MRU LAB, IMS BHU |
|  | EPI_ISL_13892316 | 2022 | 7/18/2022 | MRU LAB, IMS BHU |
|  | EPI_ISL_13892281 | 2022 | 7/18/2022 | MRU LAB, IMS BHU |
|  | EPI_ISL_13892289 | 2022 | 7/18/2022 | MRU LAB, IMS BHU |
|  | EPI_ISL_13892338 | 2022 | 7/18/2022 | MRU LAB, IMS BHU |
|  | EPI_ISL_13892290 | 2022 | 7/18/2022 | MRU LAB, IMS BHU |
|  | EPI_ISL_13892331 | 2022 | 7/18/2022 | MRU LAB, IMS BHU |
|  | EPI_ISL_13892344 | 2022 | 7/18/2022 | MRU LAB, IMS BHU |
|  | EPI_ISL_13892342 | 2022 | 7/18/2022 | MRU LAB, IMS BHU |
|  | EPI_ISL_13892339 | 2022 | 7/18/2022 | MRU LAB, IMS BHU |
|  | EPI_ISL_13887193 | 7/12/2022 | 7/18/2022 | Clinical Microbiology Laboratory, Tel Aviv Sourasky Medical Center |
|  | EPI_ISL_13887189 | 7/12/2022 | 7/18/2022 | Clinical Microbiology Laboratory, Tel Aviv Sourasky Medical Center |
|  | EPI_ISL_13893745 | 6/27/2022 | 7/18/2022 | BIO67-BIOSPHERE |
|  | EPI_ISL_13897917 | 6/25/2022 | 7/18/2022 | Quest Diagnostics Incorporated |
|  | EPI_ISL_13640173 | 6/27/2022 | 7/5/2022 | CSIR-NEERI, Nagpur Covid-19 Testing Lab |
|  | EPI_ISL_13640186 | 6/26/2022 | 7/5/2022 | CSIR-NEERI, Nagpur Covid-19 Testing Lab |
|  | EPI_ISL_13640145 | 6/23/2022 | 7/5/2022 | CSIR-NEERI, Nagpur Covid-19 Testing Lab |
|  | EPI_ISL_13640156 | 6/26/2022 | 7/5/2022 | CSIR-NEERI, Nagpur Covid-19 Testing Lab |
|  | EPI_ISL_13640192 | 6/26/2022 | 7/5/2022 | CSIR-NEERI, Nagpur Covid-19 Testing Lab |
|  | EPI_ISL_13640195 | 6/26/2022 | 7/5/2022 | CSIR-NEERI, Nagpur Covid-19 Testing Lab |
|  | EPI_ISL_13902032 | 7/7/2022 | 7/19/2022 | Department of Acute Infectious Diseases Control and Prevention, Yunnan Provincial Center for Disease Control and Prevention |
|  | EPI_ISL_13905405 | 7/6/2022 | 7/19/2022 | SA Pathology |
|  | EPI_ISL_13905312 | 7/1/2022 | 7/19/2022 | SA Pathology |
|  | EPI_ISL_13905571 | 7/4/2022 | 7/19/2022 | RSCM |
|  | EPI_ISL_14083615 | 6/18/2022 | 7/27/2022 | TPMG Regional Laboratory |
|  | EPI_ISL_14088107 | 7/18/2022 | 7/28/2022 | Lighthouse Lab in Glasgow |
|  | EPI_ISL_14090959 | 7/19/2022 | 7/28/2022 | Department of Bacteria, Parasites and Fungi, Statens Serum Institut, Copenhagen, Denmark |
|  | EPI_ISL_14099305 | 6/28/2022 | 7/28/2022 | CSIR-NEERI, Nagpur Covid-19 Testing Lab |
|  | EPI_ISL_14099271 | 6/27/2022 | 7/28/2022 | CSIR-NEERI, Nagpur Covid-19 Testing Lab |
|  | EPI_ISL_14099279 | 7/1/2022 | 7/28/2022 | CSIR-NEERI, Nagpur Covid-19 Testing Lab |
|  | EPI_ISL_14099314 | 6/30/2022 | 7/28/2022 | CSIR-NEERI, Nagpur Covid-19 Testing Lab |
|  | EPI_ISL_14099351 | 7/1/2022 | 7/28/2022 | CSIR-NEERI, Nagpur Covid-19 Testing Lab |
|  | EPI_ISL_14099284 | 7/2/2022 | 7/28/2022 | CSIR-NEERI, Nagpur Covid-19 Testing Lab |
|  | EPI_ISL_14099288 | 6/29/2022 | 7/28/2022 | CSIR-NEERI, Nagpur Covid-19 Testing Lab |
|  | EPI_ISL_14099327 | 6/27/2022 | 7/28/2022 | CSIR-NEERI, Nagpur Covid-19 Testing Lab |
|  | EPI_ISL_14099311 | 6/29/2022 | 7/28/2022 | CSIR-NEERI, Nagpur Covid-19 Testing Lab |
|  | EPI_ISL_14099282 | 6/27/2022 | 7/28/2022 | CSIR-NEERI, Nagpur Covid-19 Testing Lab |
|  | EPI_ISL_14099285 | 6/27/2022 | 7/28/2022 | CSIR-NEERI, Nagpur Covid-19 Testing Lab |
|  | EPI_ISL_14089687 | 7/7/2022 | 7/28/2022 | Virology Department, Royal Infirmary of Edinburgh, NHS Lothian / School of Biological Sciences, University of Edinburgh |
|  | EPI_ISL_14089699 | 7/6/2022 | 7/28/2022 | Virology Department, Royal Infirmary of Edinburgh, NHS Lothian / School of Biological Sciences, University of Edinburgh |
|  | EPI_ISL_14097847 | 7/11/2022 | 7/28/2022 | RT-PCR Lab, Regional Hospital Una |
|  | EPI_ISL_14097835 | 7/1/2022 | 7/28/2022 | RTPCR Lab Panchkula |
|  | EPI_ISL_14097870 | 7/4/2022 | 7/28/2022 | Dr RPGMC Tanda |
|  | EPI_ISL_14097834 | 7/14/2022 | 7/28/2022 | SLBSGMCH |
|  | EPI_ISL_14097845 | 7/9/2022 | 7/28/2022 | RT-PCR Lab, Regional Hospital Una |
|  | EPI_ISL_14097837 | 7/2/2022 | 7/28/2022 | RTPCR Lab Panchkula |
|  | EPI_ISL_14097836 | 7/2/2022 | 7/28/2022 | RTPCR Lab Panchkula |
|  | EPI_ISL_14097846 | 7/11/2022 | 7/28/2022 | RT-PCR Lab, Regional Hospital Una |
|  | EPI_ISL_14097893 | 7/13/2022 | 7/28/2022 | Dr RPGMC Tanda |
|  | EPI_ISL_14097842 | 7/9/2022 | 7/28/2022 | RTPCR Lab Panchkula |
|  | EPI_ISL_14097818 | 7/4/2022 | 7/28/2022 | Pt JLNGMCH Chamba |
|  | EPI_ISL_14097894 | 7/14/2022 | 7/28/2022 | Dr RPGMC Tanda |
|  | EPI_ISL_14097871 | 7/4/2022 | 7/28/2022 | Dr RPGMC Tanda |
|  | EPI_ISL_14097872 | 7/5/2022 | 7/28/2022 | Dr RPGMC Tanda |
|  | EPI_ISL_14097829 | 7/11/2022 | 7/28/2022 | Pt JLNGMCH Chamba |
|  | EPI_ISL_14097859 | 6/28/2022 | 7/28/2022 | Dr RPGMC Tanda |
|  | EPI_ISL_14097880 | 7/9/2022 | 7/28/2022 | Dr RPGMC Tanda |
|  | EPI_ISL_14097887 | 7/12/2022 | 7/28/2022 | Dr RPGMC Tanda |
|  | EPI_ISL_14097888 | 7/12/2022 | 7/28/2022 | Dr RPGMC Tanda |
|  | EPI_ISL_14097886 | 7/12/2022 | 7/28/2022 | Dr RPGMC Tanda |
|  | EPI_ISL_14097895 | 7/14/2022 | 7/28/2022 | Dr RPGMC Tanda |
|  | EPI_ISL_14097867 | 7/3/2022 | 7/28/2022 | Dr RPGMC Tanda |
|  | EPI_ISL_14097839 | 7/5/2022 | 7/28/2022 | RTPCR Lab Panchkula |
|  | EPI_ISL_14097812 | 7/10/2022 | 7/28/2022 | COVID-19 Laboratory Chuchot SNM Hospital |
|  | EPI_ISL_14097821 | 7/7/2022 | 7/28/2022 | Pt JLNGMCH Chamba |
|  | EPI_ISL_14097825 | 7/9/2022 | 7/28/2022 | Pt JLNGMCH Chamba |
|  | EPI_ISL_14097889 | 7/12/2022 | 7/28/2022 | Dr RPGMC Tanda |
|  | EPI_ISL_14097885 | 7/12/2022 | 7/28/2022 | Dr RPGMC Tanda |
|  | EPI_ISL_14097869 | 7/4/2022 | 7/28/2022 | Dr RPGMC Tanda |
|  | EPI_ISL_14097881 | 7/9/2022 | 7/28/2022 | Dr RPGMC Tanda |
|  | EPI_ISL_14097884 | 7/11/2022 | 7/28/2022 | Dr RPGMC Tanda |
|  | EPI_ISL_14097853 | 7/13/2022 | 7/28/2022 | RT-PCR Lab, Regional Hospital Una |
|  | EPI_ISL_14098929 | 6/26/2022 | 7/28/2022 | SIR GANGA RAM HOSPITAL |
|  | EPI_ISL_14099005 | 7/6/2022 | 7/28/2022 | P BHASIN |
|  | EPI_ISL_14099004 | 7/5/2022 | 7/28/2022 | P BHASIN |
|  | EPI_ISL_14099003 | 7/5/2022 | 7/28/2022 | P BHASIN |
|  | EPI_ISL_14099002 | 7/4/2022 | 7/28/2022 | P BHASIN |
|  | EPI_ISL_14099006 | 7/7/2022 | 7/28/2022 | P BHASIN |
|  | EPI_ISL_14099316 | 7/2/2022 | 7/28/2022 | CSIR-NEERI, Nagpur Covid-19 Testing Lab |
|  | EPI_ISL_14099300 | 6/30/2022 | 7/28/2022 | CSIR-NEERI, Nagpur Covid-19 Testing Lab |
|  | EPI_ISL_14099343 | 7/2/2022 | 7/28/2022 | CSIR-NEERI, Nagpur Covid-19 Testing Lab |
|  | EPI_ISL_14099304 | 6/27/2022 | 7/28/2022 | CSIR-NEERI, Nagpur Covid-19 Testing Lab |
|  | EPI_ISL_14099297 | 6/29/2022 | 7/28/2022 | CSIR-NEERI, Nagpur Covid-19 Testing Lab |
|  | EPI_ISL_14099312 | 7/1/2022 | 7/28/2022 | CSIR-NEERI, Nagpur Covid-19 Testing Lab |
|  | EPI_ISL_14099268 | 7/1/2022 | 7/28/2022 | CSIR-NEERI, Nagpur Covid-19 Testing Lab |
|  | EPI_ISL_14099349 | 6/29/2022 | 7/28/2022 | CSIR-NEERI, Nagpur Covid-19 Testing Lab |
|  | EPI_ISL_14099310 | 6/30/2022 | 7/28/2022 | CSIR-NEERI, Nagpur Covid-19 Testing Lab |
|  | EPI_ISL_14099350 | 6/27/2022 | 7/28/2022 | CSIR-NEERI, Nagpur Covid-19 Testing Lab |
|  | EPI_ISL_14099335 | 6/27/2022 | 7/28/2022 | CSIR-NEERI, Nagpur Covid-19 Testing Lab |
|  | EPI_ISL_14099307 | 7/2/2022 | 7/28/2022 | CSIR-NEERI, Nagpur Covid-19 Testing Lab |
|  | EPI_ISL_14099309 | 7/3/2022 | 7/28/2022 | CSIR-NEERI, Nagpur Covid-19 Testing Lab |
|  | EPI_ISL_14099306 | 7/2/2022 | 7/28/2022 | CSIR-NEERI, Nagpur Covid-19 Testing Lab |
|  | EPI_ISL_14099346 | 7/2/2022 | 7/28/2022 | CSIR-NEERI, Nagpur Covid-19 Testing Lab |
|  | EPI_ISL_14099264 | 6/29/2022 | 7/28/2022 | CSIR-NEERI, Nagpur Covid-19 Testing Lab |
|  | EPI_ISL_14099266 | 6/30/2022 | 7/28/2022 | CSIR-NEERI, Nagpur Covid-19 Testing Lab |
|  | EPI_ISL_14099287 | 6/29/2022 | 7/28/2022 | CSIR-NEERI, Nagpur Covid-19 Testing Lab |
|  | EPI_ISL_14099960 | 7/11/2022 | 7/28/2022 | PathWest Laboratory Medicine WA |
|  | EPI_ISL_14101260 | 7/18/2022 | 7/28/2022 | National Public Health Laboratory, National Centre for Infectious Diseases |
|  | EPI_ISL_14101261 | 7/19/2022 | 7/28/2022 | National Public Health Laboratory, National Centre for Infectious Diseases |
|  | EPI_ISL_14101256 | 7/20/2022 | 7/28/2022 | National Public Health Laboratory, National Centre for Infectious Diseases |
|  | EPI_ISL_14101259 | 7/18/2022 | 7/28/2022 | National Public Health Laboratory, National Centre for Infectious Diseases |
|  | EPI_ISL_14101257 | 7/18/2022 | 7/28/2022 | National Public Health Laboratory, National Centre for Infectious Diseases |
|  | EPI_ISL_14101258 | 7/19/2022 | 7/28/2022 | National Public Health Laboratory, National Centre for Infectious Diseases |
|  | EPI_ISL_14101312 | 7/20/2022 | 7/28/2022 | National Public Health Laboratory, National Centre for Infectious Diseases |
|  | EPI_ISL_14101311 | 7/20/2022 | 7/28/2022 | National Public Health Laboratory, National Centre for Infectious Diseases |
|  | EPI_ISL_14106455 | 7/15/2022 | 7/28/2022 | Laboratory Corporation of America |
|  | EPI_ISL_14113063 | 7/21/2022 | 7/29/2022 | National Institute of Public Health |
|  | EPI_ISL_14124218 | 7/14/2022 | 7/29/2022 | 4CYTE PATHOLOGY |
|  | EPI_ISL_14124173 | 7/15/2022 | 7/29/2022 | HISTOPATH PATHOLOGY |
|  | EPI_ISL_14124209 | 7/14/2022 | 7/29/2022 | 4CYTE PATHOLOGY |
|  | EPI_ISL_14125109 | 6/24/2022 | 7/29/2022 | Department of Infectious Diseases, Kobe Institute of Health |
|  | EPI_ISL_13764474 | 6/21/2022 | 7/11/2022 | LabTests |
|  | EPI_ISL_14134402 | 7/23/2022 | 7/28/2022 | Nebraska Public Health Laboratory |
|  | EPI_ISL_14140734 | 7/6/2022 | 7/29/2022 | Fukushima Prefectural Institute of Public Health |
|  | EPI_ISL_14148579 | 7/4/2022 | 7/29/2022 | SMS MEDICAL COLLEGE,JAIPUR |
|  | EPI_ISL_14148498 | 7/7/2022 | 7/29/2022 | SMS MEDICAL COLLEGE,JAIPUR |
|  | EPI_ISL_14148451 | 7/10/2022 | 7/29/2022 | SMS MEDICAL COLLEGE,JAIPUR |
|  | EPI_ISL_14148452 | 7/10/2022 | 7/29/2022 | SMS MEDICAL COLLEGE,JAIPUR |
|  | EPI_ISL_14148447 | 7/10/2022 | 7/29/2022 | SMS MEDICAL COLLEGE,JAIPUR |
|  | EPI_ISL_14148485 | 7/7/2022 | 7/29/2022 | SMS MEDICAL COLLEGE,JAIPUR |
|  | EPI_ISL_14148433 | 7/10/2022 | 7/29/2022 | SMS MEDICAL COLLEGE,JAIPUR |
|  | EPI_ISL_14148487 | 7/7/2022 | 7/29/2022 | SMS MEDICAL COLLEGE,JAIPUR |
|  | EPI_ISL_14148443 | 7/10/2022 | 7/29/2022 | SMS MEDICAL COLLEGE,JAIPUR |
|  | EPI_ISL_14148521 | 7/9/2022 | 7/29/2022 | SMS MEDICAL COLLEGE,JAIPUR |
|  | EPI_ISL_14148509 | 7/6/2022 | 7/29/2022 | SMS MEDICAL COLLEGE,JAIPUR |
|  | EPI_ISL_14148537 | 7/7/2022 | 7/29/2022 | SMS MEDICAL COLLEGE,JAIPUR |
|  | EPI_ISL_14148532 | 7/9/2022 | 7/29/2022 | SMS MEDICAL COLLEGE,JAIPUR |
|  | EPI_ISL_14148523 | 7/9/2022 | 7/29/2022 | SMS MEDICAL COLLEGE,JAIPUR |
|  | EPI_ISL_14148531 | 7/9/2022 | 7/29/2022 | SMS MEDICAL COLLEGE,JAIPUR |
|  | EPI_ISL_14148524 | 7/9/2022 | 7/29/2022 | SMS MEDICAL COLLEGE,JAIPUR |
|  | EPI_ISL_14148528 | 7/9/2022 | 7/29/2022 | SMS MEDICAL COLLEGE,JAIPUR |
|  | EPI_ISL_14148526 | 7/9/2022 | 7/29/2022 | SMS MEDICAL COLLEGE,JAIPUR |
|  | EPI_ISL_14148520 | 7/9/2022 | 7/29/2022 | SMS MEDICAL COLLEGE,JAIPUR |
|  | EPI_ISL_14148512 | 7/9/2022 | 7/29/2022 | SMS MEDICAL COLLEGE,JAIPUR |
|  | EPI_ISL_14148515 | 7/9/2022 | 7/29/2022 | SMS MEDICAL COLLEGE,JAIPUR |
|  | EPI_ISL_14148597 | 7/6/2022 | 7/29/2022 | SMS MEDICAL COLLEGE,JAIPUR |
|  | EPI_ISL_14148614 | 7/6/2022 | 7/29/2022 | SMS MEDICAL COLLEGE,JAIPUR |
|  | EPI_ISL_14147783 | 6/24/2022 | 7/29/2022 | BioneXt Lab |
|  | EPI_ISL_14148611 | 7/6/2022 | 7/29/2022 | SMS MEDICAL COLLEGE,JAIPUR |
|  | EPI_ISL_14147657 | 6/25/2022 | 7/29/2022 | BioneXt Lab |
|  | EPI_ISL_14147576 | 6/24/2022 | 7/29/2022 | BioneXt Lab |
|  | EPI_ISL_14148592 | 7/4/2022 | 7/29/2022 | SMS MEDICAL COLLEGE,JAIPUR |
|  | EPI_ISL_14148591 | 7/4/2022 | 7/29/2022 | SMS MEDICAL COLLEGE,JAIPUR |
|  | EPI_ISL_14148590 | 7/4/2022 | 7/29/2022 | SMS MEDICAL COLLEGE,JAIPUR |
|  | EPI_ISL_14148589 | 7/4/2022 | 7/29/2022 | SMS MEDICAL COLLEGE,JAIPUR |
|  | EPI_ISL_14148280 | 6/17/2022 | 7/29/2022 | State Virus Research and Diagnostic Laboratory (VRDL), Department of Microbiology, AIIMS Raipur |
|  | EPI_ISL_14148281 | 6/17/2022 | 7/29/2022 | State Virus Research and Diagnostic Laboratory (VRDL), Department of Microbiology, AIIMS Raipur |
|  | EPI_ISL_14148276 | 6/15/2022 | 7/29/2022 | State Virus Research and Diagnostic Laboratory (VRDL), Department of Microbiology, AIIMS Raipur |
|  | EPI_ISL_14148585 | 7/4/2022 | 7/29/2022 | SMS MEDICAL COLLEGE,JAIPUR |
|  | EPI_ISL_14148394 | 7/1/2022 | 7/29/2022 | Regional VRDL ICMR-RMRC, Bhubaneswar |
|  | EPI_ISL_14148367 | 6/27/2022 | 7/29/2022 | Regional VRDL ICMR-RMRC, Bhubaneswar |
|  | EPI_ISL_14148407 | 7/1/2022 | 7/29/2022 | Regional VRDL ICMR-RMRC, Bhubaneswar |
|  | EPI_ISL_14148419 | 6/8/2022 | 7/29/2022 | AIIMS Bhopal VRDL/Translational Medicine - INSACOG |
|  | EPI_ISL_14148459 | 7/13/2022 | 7/29/2022 | SMS MEDICAL COLLEGE,JAIPUR |
|  | EPI_ISL_14148586 | 7/4/2022 | 7/29/2022 | SMS MEDICAL COLLEGE,JAIPUR |
|  | EPI_ISL_14148500 | 7/7/2022 | 7/29/2022 | SMS MEDICAL COLLEGE,JAIPUR |
|  | EPI_ISL_14148475 | 7/7/2022 | 7/29/2022 | SMS MEDICAL COLLEGE,JAIPUR |
|  | EPI_ISL_14148476 | 7/7/2022 | 7/29/2022 | SMS MEDICAL COLLEGE,JAIPUR |
|  | EPI_ISL_14148465 | 7/7/2022 | 7/29/2022 | SMS MEDICAL COLLEGE,JAIPUR |
|  | EPI_ISL_14148467 | 7/7/2022 | 7/29/2022 | SMS MEDICAL COLLEGE,JAIPUR |
|  | EPI_ISL_14148469 | 7/7/2022 | 7/29/2022 | SMS MEDICAL COLLEGE,JAIPUR |
|  | EPI_ISL_14148436 | 7/10/2022 | 7/29/2022 | SMS MEDICAL COLLEGE,JAIPUR |
|  | EPI_ISL_14148505 | 7/7/2022 | 7/29/2022 | SMS MEDICAL COLLEGE,JAIPUR |
|  | EPI_ISL_14148463 | 7/8/2022 | 7/29/2022 | SMS MEDICAL COLLEGE,JAIPUR |
|  | EPI_ISL_14148499 | 7/7/2022 | 7/29/2022 | SMS MEDICAL COLLEGE,JAIPUR |
|  | EPI_ISL_14148456 | 7/13/2022 | 7/29/2022 | SMS MEDICAL COLLEGE,JAIPUR |
|  | EPI_ISL_14148481 | 7/7/2022 | 7/29/2022 | SMS MEDICAL COLLEGE,JAIPUR |
|  | EPI_ISL_14148659 | 7/4/2022 | 7/29/2022 | SMS MEDICAL COLLEGE,JAIPUR |
|  | EPI_ISL_14148671 | 7/4/2022 | 7/29/2022 | SMS MEDICAL COLLEGE,JAIPUR |
|  | EPI_ISL_14148656 | 7/4/2022 | 7/29/2022 | SMS MEDICAL COLLEGE,JAIPUR |
|  | EPI_ISL_14148670 | 7/4/2022 | 7/29/2022 | SMS MEDICAL COLLEGE,JAIPUR |
|  | EPI_ISL_14148657 | 7/4/2022 | 7/29/2022 | SMS MEDICAL COLLEGE,JAIPUR |
|  | EPI_ISL_14148666 | 7/4/2022 | 7/29/2022 | SMS MEDICAL COLLEGE,JAIPUR |
|  | EPI_ISL_14148651 | 7/4/2022 | 7/29/2022 | SMS MEDICAL COLLEGE,JAIPUR |
|  | EPI_ISL_14148650 | 7/4/2022 | 7/29/2022 | SMS MEDICAL COLLEGE,JAIPUR |
|  | EPI_ISL_14148685 | 7/3/2022 | 7/29/2022 | SMS MEDICAL COLLEGE,JAIPUR |
|  | EPI_ISL_14148638 | 7/4/2022 | 7/29/2022 | SMS MEDICAL COLLEGE,JAIPUR |
|  | EPI_ISL_14149604 | 6/23/2022 | 7/29/2022 | ICMR-National Institute of Virology - INSACOG |
|  | EPI_ISL_14149671 | 7/13/2022 | 7/29/2022 | ICMR-National Institute of Virology - INSACOG |
|  | EPI_ISL_14149710 | 6/27/2022 | 7/29/2022 | ICMR-National Institute of Virology - INSACOG |
|  | EPI_ISL_14149823 | 6/30/2022 | 7/29/2022 | ICMR-National Institute of Virology - INSACOG |
|  | EPI_ISL_14149601 | 6/27/2022 | 7/29/2022 | ICMR-National Institute of Virology - INSACOG |
|  | EPI_ISL_14149845 | 7/4/2022 | 7/29/2022 | ICMR-National Institute of Virology - INSACOG |
|  | EPI_ISL_14149642 | 6/23/2022 | 7/29/2022 | ICMR-National Institute of Virology - INSACOG |
|  | EPI_ISL_14149645 | 6/24/2022 | 7/29/2022 | ICMR-National Institute of Virology - INSACOG |
|  | EPI_ISL_14149659 | 6/5/2022 | 7/29/2022 | ICMR-National Institute of Virology - INSACOG |
|  | EPI_ISL_14152639 | 7/25/2022 | 7/29/2022 | Nebraska Public Health Laboratory |
|  | EPI_ISL_14153266 | 7/22/2022 | 7/29/2022 | Royal Hobart Hospital |
|  | EPI_ISL_14153192 | 7/16/2022 | 7/29/2022 | Royal Hobart Hospital |
|  | EPI_ISL_14154197 | 7/8/2022 | 7/29/2022 | Edmonton Provincial Lab |
|  | EPI_ISL_14155321 | 7/15/2022 | 7/30/2022 | Helix |
|  | EPI_ISL_14158509 | 7/7/2022 | 7/31/2022 | Phrae hospital |
|  | EPI_ISL_14160192 | 7/16/2022 | 7/31/2022 | Shamir Medical Center (Asaf Harofe) |
|  | EPI_ISL_14153741 | 7/14/2022 | 7/30/2022 | Mercer University School of Medicine |
|  | EPI_ISL_14161942 | 2022-07 | 8/1/2022 | APOLLO HEALTH AND LIFESTYLE LTD |
|  | EPI_ISL_14162090 | 2022-07 | 8/1/2022 | APOLLO HEALTH AND LIFESTYLE LTD |
|  | EPI_ISL_14162103 | 2022-07 | 8/1/2022 | APOLLO HEALTH AND LIFESTYLE LTD |
|  | EPI_ISL_14161962 | 2022-07 | 8/1/2022 | APOLLO HEALTH AND LIFESTYLE LTD |
|  | EPI_ISL_14161924 | 2022-07 | 8/1/2022 | APOLLO HEALTH AND LIFESTYLE LTD |
|  | EPI_ISL_14161947 | 2022-07 | 8/1/2022 | APOLLO HEALTH AND LIFESTYLE LTD |
|  | EPI_ISL_14161943 | 2022-07 | 8/1/2022 | APOLLO HEALTH AND LIFESTYLE LTD |
|  | EPI_ISL_14162169 | 2022-07 | 8/1/2022 | APOLLO HEALTH AND LIFESTYLE LTD |
|  | EPI_ISL_14162165 | 2022-07 | 8/1/2022 | APOLLO HEALTH AND LIFESTYLE LTD |
|  | EPI_ISL_14161925 | 2022-07 | 8/1/2022 | APOLLO HEALTH AND LIFESTYLE LTD |
|  | EPI_ISL_14161899 | 2022-07 | 8/1/2022 | APOLLO HEALTH AND LIFESTYLE LTD |
|  | EPI_ISL_14161898 | 2022-07 | 8/1/2022 | APOLLO HEALTH AND LIFESTYLE LTD |
|  | EPI_ISL_14161910 | 2022-07 | 8/1/2022 | APOLLO HEALTH AND LIFESTYLE LTD |
|  | EPI_ISL_14162137 | 2022-07 | 8/1/2022 | APOLLO HEALTH AND LIFESTYLE LTD |
|  | EPI_ISL_14162016 | 2022-07 | 8/1/2022 | APOLLO HEALTH AND LIFESTYLE LTD |
|  | EPI_ISL_14162014 | 2022-07 | 8/1/2022 | APOLLO HEALTH AND LIFESTYLE LTD |
|  | EPI_ISL_14161973 | 2022-07 | 8/1/2022 | APOLLO HEALTH AND LIFESTYLE LTD |
|  | EPI_ISL_14162036 | 2022-07 | 8/1/2022 | APOLLO HEALTH AND LIFESTYLE LTD |
|  | EPI_ISL_14162229 | 2022-07 | 8/1/2022 | APOLLO HEALTH AND LIFESTYLE LTD |
|  | EPI_ISL_14162186 | 2022-07 | 8/1/2022 | APOLLO HEALTH AND LIFESTYLE LTD |
|  | EPI_ISL_14162194 | 2022-07 | 8/1/2022 | APOLLO HEALTH AND LIFESTYLE LTD |
|  | EPI_ISL_14162095 | 2022-07 | 8/1/2022 | APOLLO HEALTH AND LIFESTYLE LTD |
|  | EPI_ISL_14162080 | 2022-07 | 8/1/2022 | APOLLO HEALTH AND LIFESTYLE LTD |
|  | EPI_ISL_14162082 | 2022-07 | 8/1/2022 | APOLLO HEALTH AND LIFESTYLE LTD |
|  | EPI_ISL_14162217 | 2022-07 | 8/1/2022 | APOLLO HEALTH AND LIFESTYLE LTD |
|  | EPI_ISL_14162206 | 2022-07 | 8/1/2022 | APOLLO HEALTH AND LIFESTYLE LTD |
|  | EPI_ISL_14162212 | 2022-07 | 8/1/2022 | APOLLO HEALTH AND LIFESTYLE LTD |
|  | EPI_ISL_14162207 | 2022-07 | 8/1/2022 | APOLLO HEALTH AND LIFESTYLE LTD |
|  | EPI_ISL_14162204 | 2022-07 | 8/1/2022 | APOLLO HEALTH AND LIFESTYLE LTD |
|  | EPI_ISL_14162134 | 2022-07 | 8/1/2022 | APOLLO HEALTH AND LIFESTYLE LTD |
|  | EPI_ISL_14161970 | 2022-07 | 8/1/2022 | APOLLO HEALTH AND LIFESTYLE LTD |
|  | EPI_ISL_14162125 | 2022-07 | 8/1/2022 | APOLLO HEALTH AND LIFESTYLE LTD |
|  | EPI_ISL_14162116 | 2022-07 | 8/1/2022 | APOLLO HEALTH AND LIFESTYLE LTD |
|  | EPI_ISL_14161979 | 2022-07 | 8/1/2022 | APOLLO HEALTH AND LIFESTYLE LTD |
|  | EPI_ISL_14161952 | 2022-07 | 8/1/2022 | APOLLO HEALTH AND LIFESTYLE LTD |
|  | EPI_ISL_14162121 | 2022-07 | 8/1/2022 | APOLLO HEALTH AND LIFESTYLE LTD |
|  | EPI_ISL_14162122 | 2022-07 | 8/1/2022 | APOLLO HEALTH AND LIFESTYLE LTD |
|  | EPI_ISL_14162071 | 2022-07 | 8/1/2022 | APOLLO HEALTH AND LIFESTYLE LTD |
|  | EPI_ISL_14162157 | 2022-07 | 8/1/2022 | APOLLO HEALTH AND LIFESTYLE LTD |
|  | EPI_ISL_14161998 | 2022-07 | 8/1/2022 | APOLLO HEALTH AND LIFESTYLE LTD |
|  | EPI_ISL_14162046 | 2022-07 | 8/1/2022 | APOLLO HEALTH AND LIFESTYLE LTD |
|  | EPI_ISL_14161996 | 2022-07 | 8/1/2022 | APOLLO HEALTH AND LIFESTYLE LTD |
|  | EPI_ISL_14162870 | 7/8/2022 | 8/1/2022 | State Virus Research and Diagnostic Laboratory (VRDL), AIIMS Raipur |
|  | EPI_ISL_14162861 | 6/25/2022 | 8/1/2022 | State Virus Research and Diagnostic Laboratory (VRDL), AIIMS Raipur |
|  | EPI_ISL_14162852 | 7/13/2022 | 8/1/2022 | State Virus Research and Diagnostic Laboratory (VRDL), AIIMS Raipur |
|  | EPI_ISL_14163046 | 7/4/2022 | 8/1/2022 | Genomics for Life |
|  | EPI_ISL_14163267 | 7/8/2022 | 8/1/2022 | SARS-CoV-2 testing team, National Institute of Infectious Diseases |
|  | EPI_ISL_14163309 | 7/12/2022 | 8/1/2022 | SARS-CoV-2 testing team, National Institute of Infectious Diseases |
|  | EPI_ISL_14163266 | 7/8/2022 | 8/1/2022 | SARS-CoV-2 testing team, National Institute of Infectious Diseases |
|  | EPI_ISL_14163270 | 7/8/2022 | 8/1/2022 | SARS-CoV-2 testing team, National Institute of Infectious Diseases |
|  | EPI_ISL_14166774 | 7/1/2022 | 8/1/2022 | INSACOG-WB |
|  | EPI_ISL_14166770 | 7/1/2022 | 8/1/2022 | INSACOG-WB |
|  | EPI_ISL_14166786 | 7/1/2022 | 8/1/2022 | INSACOG-WB |
|  | EPI_ISL_14166779 | 7/1/2022 | 8/1/2022 | INSACOG-WB |
|  | EPI_ISL_14166781 | 7/1/2022 | 8/1/2022 | INSACOG-WB |
|  | EPI_ISL_14166811 | 7/2/2022 | 8/1/2022 | INSACOG-WB |
|  | EPI_ISL_14166744 | 6/29/2022 | 8/1/2022 | INSACOG-WB |
|  | EPI_ISL_14166749 | 6/29/2022 | 8/1/2022 | INSACOG-WB |
|  | EPI_ISL_14166766 | 7/1/2022 | 8/1/2022 | INSACOG-WB |
|  | EPI_ISL_14166833 | 7/4/2022 | 8/1/2022 | INSACOG-WB |
|  | EPI_ISL_14166834 | 7/4/2022 | 8/1/2022 | INSACOG-WB |
|  | EPI_ISL_14166957 | 7/5/2022 | 8/1/2022 | INSACOG-WB |
|  | EPI_ISL_14166825 | 7/4/2022 | 8/1/2022 | INSACOG-WB |
|  | EPI_ISL_14166809 | 7/2/2022 | 8/1/2022 | INSACOG-WB |
|  | EPI_ISL_14166832 | 7/4/2022 | 8/1/2022 | INSACOG-WB |
|  | EPI_ISL_14166853 | 6/22/2022 | 8/1/2022 | INSACOG-WB |
|  | EPI_ISL_14166778 | 7/1/2022 | 8/1/2022 | INSACOG-WB |
|  | EPI_ISL_14166845 | 7/4/2022 | 8/1/2022 | INSACOG-WB |
|  | EPI_ISL_14166771 | 7/1/2022 | 8/1/2022 | INSACOG-WB |
|  | EPI_ISL_14166780 | 7/1/2022 | 8/1/2022 | INSACOG-WB |
|  | EPI_ISL_14166889 | 6/30/2022 | 8/1/2022 | INSACOG-WB |
|  | EPI_ISL_14167187 | 7/7/2022 | 8/1/2022 | INSACOG-WB |
|  | EPI_ISL_14166979 | 7/3/2022 | 8/1/2022 | INSACOG-WB |
|  | EPI_ISL_14166975 | 7/6/2022 | 8/1/2022 | INSACOG-WB |
|  | EPI_ISL_14167005 | 7/1/2022 | 8/1/2022 | INSACOG-WB |
|  | EPI_ISL_14167019 | 7/2/2022 | 8/1/2022 | INSACOG-WB |
|  | EPI_ISL_14167022 | 7/2/2022 | 8/1/2022 | INSACOG-WB |
|  | EPI_ISL_14167100 | 7/5/2022 | 8/1/2022 | INSACOG-WB |
|  | EPI_ISL_14167165 | 7/6/2022 | 8/1/2022 | INSACOG-WB |
|  | EPI_ISL_14167074 | 7/4/2022 | 8/1/2022 | INSACOG-WB |
|  | EPI_ISL_14166977 | 7/1/2022 | 8/1/2022 | INSACOG-WB |
|  | EPI_ISL_14166943 | 7/2/2022 | 8/1/2022 | INSACOG-WB |
|  | EPI_ISL_14166910 | 7/3/2022 | 8/1/2022 | INSACOG-WB |
|  | EPI_ISL_14166922 | 7/2/2022 | 8/1/2022 | INSACOG-WB |
|  | EPI_ISL_14166860 | 6/23/2022 | 8/1/2022 | INSACOG-WB |
|  | EPI_ISL_14167002 | 7/8/2022 | 8/1/2022 | INSACOG-WB |
|  | EPI_ISL_14166999 | 7/5/2022 | 8/1/2022 | INSACOG-WB |
|  | EPI_ISL_14166981 | 7/5/2022 | 8/1/2022 | INSACOG-WB |
|  | EPI_ISL_14167091 | 7/4/2022 | 8/1/2022 | INSACOG-WB |
|  | EPI_ISL_14167107 | 7/5/2022 | 8/1/2022 | INSACOG-WB |
|  | EPI_ISL_14167103 | 7/5/2022 | 8/1/2022 | INSACOG-WB |
|  | EPI_ISL_14167116 | 7/5/2022 | 8/1/2022 | INSACOG-WB |
|  | EPI_ISL_14167205 | 7/7/2022 | 8/1/2022 | INSACOG-WB |
|  | EPI_ISL_14167210 | 7/8/2022 | 8/1/2022 | INSACOG-WB |
|  | EPI_ISL_14167211 | 7/7/2022 | 8/1/2022 | INSACOG-WB |
|  | EPI_ISL_14167159 | 7/6/2022 | 8/1/2022 | INSACOG-WB |
|  | EPI_ISL_14167037 | 7/2/2022 | 8/1/2022 | INSACOG-WB |
|  | EPI_ISL_14167040 | 7/2/2022 | 8/1/2022 | INSACOG-WB |
|  | EPI_ISL_14167141 | 7/6/2022 | 8/1/2022 | INSACOG-WB |
|  | EPI_ISL_14167188 | 7/7/2022 | 8/1/2022 | INSACOG-WB |
|  | EPI_ISL_14167197 | 7/7/2022 | 8/1/2022 | INSACOG-WB |
|  | EPI_ISL_14167075 | 7/4/2022 | 8/1/2022 | INSACOG-WB |
|  | EPI_ISL_14167189 | 7/6/2022 | 8/1/2022 | INSACOG-WB |
|  | EPI_ISL_14167199 | 7/7/2022 | 8/1/2022 | INSACOG-WB |
|  | EPI_ISL_14167080 | 7/4/2022 | 8/1/2022 | INSACOG-WB |
|  | EPI_ISL_14167049 | 7/2/2022 | 8/1/2022 | INSACOG-WB |
|  | EPI_ISL_14167177 | 7/7/2022 | 8/1/2022 | INSACOG-WB |
|  | EPI_ISL_14167057 | 7/4/2022 | 8/1/2022 | INSACOG-WB |
|  | EPI_ISL_14167172 | 7/7/2022 | 8/1/2022 | INSACOG-WB |
|  | EPI_ISL_14168620 | 7/4/2022 | 8/1/2022 | Laboratorium Fullerton Health Clinic Bali |
|  | EPI_ISL_14169813 | 7/22/2022 | 7/31/2022 | Shamir Medical Center (Asaf Harofe) |
|  | EPI_ISL_14172770 | 7/15/2022 | 8/1/2022 | Respiratory Virus Unit, Microbiology Services Colindale, Public Health England |
|  | EPI_ISL_14173842 | 7/18/2022 | 8/1/2022 | Respiratory Virus Unit, Microbiology Services Colindale, Public Health England |
|  | EPI_ISL_14175122 | 7/19/2022 | 8/1/2022 | National Public Health Laboratory |
|  | EPI_ISL_14175121 | 7/19/2022 | 8/1/2022 | National Public Health Laboratory |
|  | EPI_ISL_14175136 | 7/11/2022 | 8/1/2022 | National Public Health Laboratory |
|  | EPI_ISL_14175133 | 7/11/2022 | 8/1/2022 | National Public Health Laboratory |
|  | EPI_ISL_14175141 | 7/12/2022 | 8/1/2022 | National Public Health Laboratory |
|  | EPI_ISL_14175156 | 7/18/2022 | 8/1/2022 | National Public Health Laboratory |
|  | EPI_ISL_14175220 | 6/29/2022 | 8/1/2022 | INSACOG-WB |
|  | EPI_ISL_14175224 | 7/6/2022 | 8/1/2022 | INSACOG-WB |
|  | EPI_ISL_14175239 | 7/6/2022 | 8/1/2022 | INSACOG-WB |
|  | EPI_ISL_14175250 | 7/9/2022 | 8/1/2022 | INSACOG-WB |
|  | EPI_ISL_14175312 | 7/8/2022 | 8/1/2022 | INSACOG-WB |
|  | EPI_ISL_14175301 | 7/8/2022 | 8/1/2022 | INSACOG-WB |
|  | EPI_ISL_14175306 | 7/8/2022 | 8/1/2022 | INSACOG-WB |
|  | EPI_ISL_14175299 | 6/29/2022 | 8/1/2022 | INSACOG-WB |
|  | EPI_ISL_14175431 | 7/4/2022 | 8/1/2022 | INSACOG-WB |
|  | EPI_ISL_14175360 | 6/29/2022 | 8/1/2022 | INSACOG-WB |
|  | EPI_ISL_14175254 | 7/9/2022 | 8/1/2022 | INSACOG-WB |
|  | EPI_ISL_14175233 | 7/4/2022 | 8/1/2022 | INSACOG-WB |
|  | EPI_ISL_14175396 | 6/30/2022 | 8/1/2022 | INSACOG-WB |
|  | EPI_ISL_14175394 | 6/30/2022 | 8/1/2022 | INSACOG-WB |
|  | EPI_ISL_14175392 | 6/29/2022 | 8/1/2022 | INSACOG-WB |
|  | EPI_ISL_14175408 | 7/2/2022 | 8/1/2022 | INSACOG-WB |
|  | EPI_ISL_14175427 | 7/4/2022 | 8/1/2022 | INSACOG-WB |
|  | EPI_ISL_14175478 | 7/5/2022 | 8/1/2022 | INSACOG-WB |
|  | EPI_ISL_14175472 | 7/5/2022 | 8/1/2022 | INSACOG-WB |
|  | EPI_ISL_14175471 | 7/5/2022 | 8/1/2022 | INSACOG-WB |
|  | EPI_ISL_14175482 | 7/6/2022 | 8/1/2022 | INSACOG-WB |
|  | EPI_ISL_14175508 | 6/28/2022 | 8/1/2022 | INSACOG-WB |
|  | EPI_ISL_14175302 | 7/8/2022 | 8/1/2022 | INSACOG-WB |
|  | EPI_ISL_14175310 | 7/8/2022 | 8/1/2022 | INSACOG-WB |
|  | EPI_ISL_14175537 | 6/30/2022 | 8/1/2022 | INSACOG-WB |
|  | EPI_ISL_14175578 | 6/29/2022 | 8/1/2022 | INSACOG-WB |
|  | EPI_ISL_14175475 | 7/5/2022 | 8/1/2022 | INSACOG-WB |
|  | EPI_ISL_14175527 | 7/5/2022 | 8/1/2022 | INSACOG-WB |
|  | EPI_ISL_14175499 | 7/5/2022 | 8/1/2022 | INSACOG-WB |
|  | EPI_ISL_14175535 | 6/30/2022 | 8/1/2022 | INSACOG-WB |
|  | EPI_ISL_14175504 | 7/7/2022 | 8/1/2022 | INSACOG-WB |
|  | EPI_ISL_14175513 | 6/28/2022 | 8/1/2022 | INSACOG-WB |
|  | EPI_ISL_14175675 | 7/11/2022 | 8/1/2022 | INSACOG-WB |
|  | EPI_ISL_14175669 | 7/11/2022 | 8/1/2022 | INSACOG-WB |
|  | EPI_ISL_14175758 | 7/15/2022 | 8/1/2022 | INSACOG-WB |
|  | EPI_ISL_14175729 | 7/14/2022 | 8/1/2022 | INSACOG-WB |
|  | EPI_ISL_14175744 | 7/15/2022 | 8/1/2022 | INSACOG-WB |
|  | EPI_ISL_14175753 | 7/15/2022 | 8/1/2022 | INSACOG-WB |
|  | EPI_ISL_14175619 | 6/30/2022 | 8/1/2022 | INSACOG-WB |
|  | EPI_ISL_14175645 | 7/5/2022 | 8/1/2022 | INSACOG-WB |
|  | EPI_ISL_14175658 | 7/9/2022 | 8/1/2022 | INSACOG-WB |
|  | EPI_ISL_14175657 | 7/9/2022 | 8/1/2022 | INSACOG-WB |
|  | EPI_ISL_14175654 | 7/9/2022 | 8/1/2022 | INSACOG-WB |
|  | EPI_ISL_14175653 | 7/9/2022 | 8/1/2022 | INSACOG-WB |
|  | EPI_ISL_14175622 | 6/30/2022 | 8/1/2022 | INSACOG-WB |
|  | EPI_ISL_14175634 | 7/2/2022 | 8/1/2022 | INSACOG-WB |
|  | EPI_ISL_14175632 | 7/2/2022 | 8/1/2022 | INSACOG-WB |
|  | EPI_ISL_14175575 | 6/28/2022 | 8/1/2022 | INSACOG-WB |
|  | EPI_ISL_14175671 | 7/11/2022 | 8/1/2022 | INSACOG-WB |
|  | EPI_ISL_14175597 | 6/27/2022 | 8/1/2022 | INSACOG-WB |
|  | EPI_ISL_14175886 | 7/10/2022 | 8/1/2022 | INSACOG-WB |
|  | EPI_ISL_14175880 | 6/28/2022 | 8/1/2022 | INSACOG-WB |
|  | EPI_ISL_14175885 | 7/5/2022 | 8/1/2022 | INSACOG-WB |
|  | EPI_ISL_14175896 | 7/6/2022 | 8/1/2022 | INSACOG-WB |
|  | EPI_ISL_14175853 | 7/14/2022 | 8/1/2022 | INSACOG-WB |
|  | EPI_ISL_14175735 | 7/14/2022 | 8/1/2022 | INSACOG-WB |
|  | EPI_ISL_14175725 | 7/13/2022 | 8/1/2022 | INSACOG-WB |
|  | EPI_ISL_14175874 | 7/4/2022 | 8/1/2022 | INSACOG-WB |
|  | EPI_ISL_14175780 | 7/5/2022 | 8/1/2022 | INSACOG-WB |
|  | EPI_ISL_14175738 | 7/14/2022 | 8/1/2022 | INSACOG-WB |
|  | EPI_ISL_14175858 | 7/15/2022 | 8/1/2022 | INSACOG-WB |
|  | EPI_ISL_14175730 | 7/14/2022 | 8/1/2022 | INSACOG-WB |
|  | EPI_ISL_14175707 | 7/12/2022 | 8/1/2022 | INSACOG-WB |
|  | EPI_ISL_14175700 | 7/12/2022 | 8/1/2022 | INSACOG-WB |
|  | EPI_ISL_14175705 | 7/12/2022 | 8/1/2022 | INSACOG-WB |
|  | EPI_ISL_14175824 | 7/13/2022 | 8/1/2022 | INSACOG-WB |
|  | EPI_ISL_14175839 | 7/14/2022 | 8/1/2022 | INSACOG-WB |
|  | EPI_ISL_14175767 | 7/15/2022 | 8/1/2022 | INSACOG-WB |
|  | EPI_ISL_14175897 | 7/6/2022 | 8/1/2022 | INSACOG-WB |
|  | EPI_ISL_14175883 | 7/6/2022 | 8/1/2022 | INSACOG-WB |
|  | EPI_ISL_14175763 | 7/12/2022 | 8/1/2022 | INSACOG-WB |
|  | EPI_ISL_14175749 | 7/15/2022 | 8/1/2022 | INSACOG-WB |
|  | EPI_ISL_14175865 | 7/11/2022 | 8/1/2022 | INSACOG-WB |
|  | EPI_ISL_14175894 | 7/6/2022 | 8/1/2022 | INSACOG-WB |
|  | EPI_ISL_14175741 | 7/14/2022 | 8/1/2022 | INSACOG-WB |
|  | EPI_ISL_14175869 | 7/8/2022 | 8/1/2022 | INSACOG-WB |
|  | EPI_ISL_14175866 | 7/11/2022 | 8/1/2022 | INSACOG-WB |
|  | EPI_ISL_14175755 | 7/15/2022 | 8/1/2022 | INSACOG-WB |
|  | EPI_ISL_14175747 | 7/15/2022 | 8/1/2022 | INSACOG-WB |
|  | EPI_ISL_14175742 | 7/14/2022 | 8/1/2022 | INSACOG-WB |
|  | EPI_ISL_14175793 | 7/9/2022 | 8/1/2022 | INSACOG-WB |
|  | EPI_ISL_14175786 | 7/9/2022 | 8/1/2022 | INSACOG-WB |
|  | EPI_ISL_14175798 | 7/11/2022 | 8/1/2022 | INSACOG-WB |
|  | EPI_ISL_14175792 | 7/9/2022 | 8/1/2022 | INSACOG-WB |
|  | EPI_ISL_14175926 | 7/11/2022 | 8/1/2022 | INSACOG-WB |
|  | EPI_ISL_14175920 | 7/11/2022 | 8/1/2022 | INSACOG-WB |
|  | EPI_ISL_14175932 | 7/1/2022 | 8/1/2022 | INSACOG-WB |
|  | EPI_ISL_14175808 | 7/11/2022 | 8/1/2022 | INSACOG-WB |
|  | EPI_ISL_14175921 | 7/11/2022 | 8/1/2022 | INSACOG-WB |
|  | EPI_ISL_14175924 | 7/11/2022 | 8/1/2022 | INSACOG-WB |
|  | EPI_ISL_14175931 | 7/12/2022 | 8/1/2022 | INSACOG-WB |
|  | EPI_ISL_14175918 | 7/11/2022 | 8/1/2022 | INSACOG-WB |
|  | EPI_ISL_14177099 | 7/19/2022 | 8/1/2022 | Pandemic Response Lab - NYC |
|  | EPI_ISL_14177126 | 7/18/2022 | 8/1/2022 | Pandemic Response Lab - NYC |
|  | EPI_ISL_14178102 | 7/22/2022 | 8/1/2022 | Pandemic Response Lab - NYC |
|  | EPI_ISL_14176867 | 7/18/2022 | 8/1/2022 | Pandemic Response Lab - NYC |
|  | EPI_ISL_14176866 | 7/18/2022 | 8/1/2022 | Pandemic Response Lab - NYC |
|  | EPI_ISL_14177791 | 7/20/2022 | 8/1/2022 | Pandemic Response Lab - NYC |
|  | EPI_ISL_14177760 | 7/20/2022 | 8/1/2022 | Pandemic Response Lab - NYC |
|  | EPI_ISL_14183330 | 7/15/2022 | 8/1/2022 | Laboratory Corporation of America |
|  | EPI_ISL_14183774 | 7/17/2022 | 8/1/2022 | Laboratory Corporation of America |
|  | EPI_ISL_14183773 | 7/17/2022 | 8/1/2022 | Laboratory Corporation of America |
|  | EPI_ISL_14184360 | 7/19/2022 | 8/1/2022 | Laboratory Corporation of America |
|  | EPI_ISL_14186759 | 7/22/2022 | 8/1/2022 | HUTCHINSON HEALTH HOSPITAL |
|  | EPI_ISL_14188895 | 7/26/2022 | 8/1/2022 | National Public Health Laboratory, National Centre for Infectious Diseases |
|  | EPI_ISL_14188886 | 7/25/2022 | 8/1/2022 | National Public Health Laboratory, National Centre for Infectious Diseases |
|  | EPI_ISL_14188847 | 7/25/2022 | 8/1/2022 | National Public Health Laboratory, National Centre for Infectious Diseases |
|  | EPI_ISL_14188880 | 7/25/2022 | 8/1/2022 | National Public Health Laboratory, National Centre for Infectious Diseases |
|  | EPI_ISL_14188994 | 7/26/2022 | 8/1/2022 | National Public Health Laboratory, National Centre for Infectious Diseases |
|  | EPI_ISL_14188913 | 7/26/2022 | 8/1/2022 | National Public Health Laboratory, National Centre for Infectious Diseases |
|  | EPI_ISL_14188972 | 7/26/2022 | 8/1/2022 | National Public Health Laboratory, National Centre for Infectious Diseases |
|  | EPI_ISL_14188944 | 7/27/2022 | 8/1/2022 | National Public Health Laboratory, National Centre for Infectious Diseases |
|  | EPI_ISL_14188933 | 7/27/2022 | 8/1/2022 | National Public Health Laboratory, National Centre for Infectious Diseases |
|  | EPI_ISL_14188930 | 7/26/2022 | 8/1/2022 | National Public Health Laboratory, National Centre for Infectious Diseases |
|  | EPI_ISL_14191789 | 7/9/2022 | 8/2/2022 | Quest Diagnostics Incorporated |
|  | EPI_ISL_14192114 | 7/10/2022 | 8/2/2022 | Quest Diagnostics Incorporated |
|  | EPI_ISL_14192543 | 7/24/2022 | 8/2/2022 | National Public Health Laboratory, National Centre for Infectious Diseases |
|  | EPI_ISL_14192547 | 7/21/2022 | 8/2/2022 | National Public Health Laboratory, National Centre for Infectious Diseases |
|  | EPI_ISL_14192539 | 7/23/2022 | 8/2/2022 | National Public Health Laboratory, National Centre for Infectious Diseases |
|  | EPI_ISL_14192548 | 7/21/2022 | 8/2/2022 | National Public Health Laboratory, National Centre for Infectious Diseases |
|  | EPI_ISL_14192545 | 7/23/2022 | 8/2/2022 | National Public Health Laboratory, National Centre for Infectious Diseases |
|  | EPI_ISL_14192546 | 7/22/2022 | 8/2/2022 | National Public Health Laboratory, National Centre for Infectious Diseases |
|  | EPI_ISL_14192542 | 7/22/2022 | 8/2/2022 | National Public Health Laboratory, National Centre for Infectious Diseases |
|  | EPI_ISL_14192544 | 7/23/2022 | 8/2/2022 | National Public Health Laboratory, National Centre for Infectious Diseases |
|  | EPI_ISL_14192540 | 7/22/2022 | 8/2/2022 | National Public Health Laboratory, National Centre for Infectious Diseases |
|  | EPI_ISL_14192541 | 7/22/2022 | 8/2/2022 | National Public Health Laboratory, National Centre for Infectious Diseases |
|  | EPI_ISL_14192681 | 7/24/2022 | 8/2/2022 | National Public Health Laboratory, National Centre for Infectious Diseases |
|  | EPI_ISL_14192682 | 7/21/2022 | 8/2/2022 | National Public Health Laboratory, National Centre for Infectious Diseases |
|  | EPI_ISL_14196427 | 7/3/2022 | 8/2/2022 | Gandhi Medical College |
|  | EPI_ISL_14196419 | 7/2/2022 | 8/2/2022 | Gandhi Medical College |
|  | EPI_ISL_14196428 | 7/4/2022 | 8/2/2022 | Gandhi Medical College |
|  | EPI_ISL_14196569 | 7/20/2022 | 8/2/2022 | Gandhi Medical College |
|  | EPI_ISL_14196546 | 7/11/2022 | 8/2/2022 | Gandhi Medical College |
|  | EPI_ISL_14196545 | 7/12/2022 | 8/2/2022 | Gandhi Medical College |
|  | EPI_ISL_14196540 | 7/8/2022 | 8/2/2022 | Gandhi Medical College |
|  | EPI_ISL_14196533 | 7/10/2022 | 8/2/2022 | Gandhi Medical College |
|  | EPI_ISL_14196537 | 7/11/2022 | 8/2/2022 | Gandhi Medical College |
|  | EPI_ISL_14196496 | 7/5/2022 | 8/2/2022 | Gandhi Medical College |
|  | EPI_ISL_14196526 | 7/8/2022 | 8/2/2022 | Gandhi Medical College |
|  | EPI_ISL_14196524 | 7/8/2022 | 8/2/2022 | Gandhi Medical College |
|  | EPI_ISL_14196528 | 7/8/2022 | 8/2/2022 | Gandhi Medical College |
|  | EPI_ISL_14196515 | 7/8/2022 | 8/2/2022 | Gandhi Medical College |
|  | EPI_ISL_14196562 | 7/19/2022 | 8/2/2022 | Gandhi Medical College |
|  | EPI_ISL_14196506 | 7/7/2022 | 8/2/2022 | Gandhi Medical College |
|  | EPI_ISL_14196565 | 7/20/2022 | 8/2/2022 | Gandhi Medical College |
|  | EPI_ISL_14196564 | 6/7/2022 | 8/2/2022 | Gandhi Medical College |
|  | EPI_ISL_14199084 | 7/1/2022 | 8/2/2022 | Biomedical Centre Martin, Jessenius Faculty of Medicine in Martin, Comenius University |
|  | EPI_ISL_14203551 | 7/20/2022 | 8/2/2022 | JCMC, Jorhat |
|  | EPI_ISL_14203545 | 7/17/2022 | 8/2/2022 | JCMC, Jorhat |
|  | EPI_ISL_14203473 | 7/11/2022 | 8/2/2022 | AMCH, Dibrugarh |
|  | EPI_ISL_14203508 | 7/13/2022 | 8/2/2022 | IDSP, Kamrup Metro (ULTRACARE DIAGNOSTIC CENTRE) |
|  | EPI_ISL_14203474 | 7/11/2022 | 8/2/2022 | AMCH, Dibrugarh |
|  | EPI_ISL_14203546 | 7/17/2022 | 8/2/2022 | JCMC, Jorhat |
|  | EPI_ISL_14203542 | 7/14/2022 | 8/2/2022 | Barua Clinical Laboratory, Sanjivani Hospital |
|  | EPI_ISL_14203540 | 7/14/2022 | 8/2/2022 | Barua Clinical Laboratory, Sanjivani Hospital |
|  | EPI_ISL_14209618 | 7/20/2022 | 8/2/2022 | Public Health Ontario Laboratory |
|  | EPI_ISL_14210416 | 7/20/2022 | 8/2/2022 | Public Health Ontario Laboratory |
|  | EPI_ISL_14210413 | 7/25/2022 | 8/2/2022 | Public Health Ontario Laboratory |
|  | EPI_ISL_14211901 | 7/16/2022 | 8/3/2022 | SARS-CoV-2 testing team, National Institute of Infectious Diseases |
|  | EPI_ISL_14211895 | 7/16/2022 | 8/3/2022 | SARS-CoV-2 testing team, National Institute of Infectious Diseases |
|  | EPI_ISL_14211891 | 7/15/2022 | 8/3/2022 | SARS-CoV-2 testing team, National Institute of Infectious Diseases |
|  | EPI_ISL_14211902 | 7/16/2022 | 8/3/2022 | SARS-CoV-2 testing team, National Institute of Infectious Diseases |
|  | EPI_ISL_14211925 | 7/17/2022 | 8/3/2022 | SARS-CoV-2 testing team, National Institute of Infectious Diseases |
|  | EPI_ISL_14211968 | 7/19/2022 | 8/3/2022 | SARS-CoV-2 testing team, National Institute of Infectious Diseases |
|  | EPI_ISL_14211984 | 7/20/2022 | 8/3/2022 | SARS-CoV-2 testing team, National Institute of Infectious Diseases |
|  | EPI_ISL_14214069 | 7/29/2022 | 8/3/2022 | Department of Bacteria, Parasites and Fungi, Statens Serum Institut, Copenhagen, Denmark |
|  | EPI_ISL_14215427 | 7/12/2022 | 8/3/2022 | Gandhi Medical College |
|  | EPI_ISL_14215476 | 7/18/2022 | 8/3/2022 | Gandhi Medical College |
|  | EPI_ISL_14215527 | 7/20/2022 | 8/3/2022 | Gandhi Medical College |
|  | EPI_ISL_14215552 | 7/25/2022 | 8/3/2022 | Gandhi Medical College |
|  | EPI_ISL_14215463 | 7/16/2022 | 8/3/2022 | Gandhi Medical College |
|  | EPI_ISL_14215432 | 7/12/2022 | 8/3/2022 | Gandhi Medical College |
|  | EPI_ISL_14215462 | 7/16/2022 | 8/3/2022 | Gandhi Medical College |
|  | EPI_ISL_14215444 | 7/14/2022 | 8/3/2022 | Gandhi Medical College |
|  | EPI_ISL_14215437 | 7/13/2022 | 8/3/2022 | Gandhi Medical College |
|  | EPI_ISL_14215481 | 7/18/2022 | 8/3/2022 | Gandhi Medical College |
|  | EPI_ISL_14215512 | 7/22/2022 | 8/3/2022 | Gandhi Medical College |
|  | EPI_ISL_14215537 | 7/19/2022 | 8/3/2022 | Gandhi Medical College |
|  | EPI_ISL_14215577 | 7/22/2022 | 8/3/2022 | Gandhi Medical College |
|  | EPI_ISL_14215509 | 7/22/2022 | 8/3/2022 | Gandhi Medical College |
|  | EPI_ISL_14215542 | 7/24/2022 | 8/3/2022 | Gandhi Medical College |
|  | EPI_ISL_14215554 | 7/25/2022 | 8/3/2022 | Gandhi Medical College |
|  | EPI_ISL_14215573 | 7/21/2022 | 8/3/2022 | Gandhi Medical College |
|  | EPI_ISL_14215505 | 7/21/2022 | 8/3/2022 | Gandhi Medical College |
|  | EPI_ISL_14215497 | 7/20/2022 | 8/3/2022 | Gandhi Medical College |
|  | EPI_ISL_14215572 | 7/22/2022 | 8/3/2022 | Gandhi Medical College |
|  | EPI_ISL_14215472 | 7/18/2022 | 8/3/2022 | Gandhi Medical College |
|  | EPI_ISL_14215507 | 7/17/2022 | 8/3/2022 | Gandhi Medical College |
|  | EPI_ISL_14215510 | 7/16/2022 | 8/3/2022 | Gandhi Medical College |
|  | EPI_ISL_14215498 | 7/20/2022 | 8/3/2022 | Gandhi Medical College |
|  | EPI_ISL_14215508 | 7/22/2022 | 8/3/2022 | Gandhi Medical College |
|  | EPI_ISL_14215584 | 7/21/2022 | 8/3/2022 | Gandhi Medical College |
|  | EPI_ISL_14215501 | 7/20/2022 | 8/3/2022 | Gandhi Medical College |
|  | EPI_ISL_14215499 | 7/20/2022 | 8/3/2022 | Gandhi Medical College |
|  | EPI_ISL_14215513 | 7/22/2022 | 8/3/2022 | Gandhi Medical College |
|  | EPI_ISL_14215528 | 7/21/2022 | 8/3/2022 | Gandhi Medical College |
|  | EPI_ISL_14215482 | 7/18/2022 | 8/3/2022 | Gandhi Medical College |
|  | EPI_ISL_14215524 | 7/18/2022 | 8/3/2022 | Gandhi Medical College |
|  | EPI_ISL_14215514 | 7/22/2022 | 8/3/2022 | Gandhi Medical College |
|  | EPI_ISL_14215480 | 7/18/2022 | 8/3/2022 | Gandhi Medical College |
|  | EPI_ISL_14215515 | 7/22/2022 | 8/3/2022 | Gandhi Medical College |
|  | EPI_ISL_14215516 | 7/16/2022 | 8/3/2022 | Gandhi Medical College |
|  | EPI_ISL_14215511 | 7/22/2022 | 8/3/2022 | Gandhi Medical College |
|  | EPI_ISL_14217529 | 7/26/2022 | 8/3/2022 | Lifebrain Covid Labor GmbH |
|  | EPI_ISL_14215664 | 7/28/2022 | 8/3/2022 | Axi - Darmon |
|  | EPI_ISL_14215649 | 7/27/2022 | 8/3/2022 | Genbio - Pt / Clermont Gravanches |
|  | EPI_ISL_13830162 | 6/28/2022 | 7/14/2022 | Rush University Medical Center |
|  | EPI_ISL_13906117 | 7/5/2022 | 7/19/2022 | Respiratory Virus Unit, Microbiology Services Colindale, Public Health England |
|  | EPI_ISL_13660061 | 6/15/2022 | 7/6/2022 | STERLING ACCURIS DIAGNOSTICS |
|  | EPI_ISL_13660043 | 6/15/2022 | 7/6/2022 | DR. P BHASIN PATH LAB |
|  | EPI_ISL_13610927 | 2022-06 | 7/3/2022 | Neuberg Anand Reference Laboratory, Bengaluru |
|  | EPI_ISL_13671597 | 6/22/2022 | 7/6/2022 | RTPCR Lab Sirsa |
|  | EPI_ISL_13583747 | 6/15/2022 | 7/1/2022 | Laboratory Corporation of America |
|  | EPI_ISL_13583301 | 6/14/2022 | 7/1/2022 | Laboratory Corporation of America |
|  | EPI_ISL_13373059 | 2022-06 | 6/20/2022 | APOLLO HEALTH AND LIFESTYLE LTD |
|  | EPI_ISL_13373170 | 2022-06 | 6/20/2022 | APOLLO HEALTH AND LIFESTYLE LTD |
|  | EPI_ISL_13574681 | 6/15/2022 | 7/1/2022 | AIIMS Bhopal VRDL/Translational Medicine - INSACOG |
|  | EPI_ISL_13574678 | 6/11/2022 | 7/1/2022 | AIIMS Bhopal VRDL/Translational Medicine - INSACOG |
|  | EPI_ISL_13378378 | 6/6/2022 | 6/21/2022 | Asklepios SÃ¼dpfalzkliniken GmbH: Standort Kandel ("Asklepios SÃ¼dpfalzklinik Kandel - Asklepios Kliniken") |
|  | EPI_ISL_13378924 | 6/3/2022 | 6/21/2022 | Limbach - MVZ Labor Dr. Volkmann & Kollegen |
|  | EPI_ISL_13567021 | 6/24/2022 | 7/1/2022 | Rosalind Franklin Laboratory |
|  | EPI_ISL_13565946 | 6/13/2022 | 7/1/2022 | SARS-CoV-2 testing team, National Institute of Infectious Diseases |
|  | EPI_ISL_13392500 | 6/9/2022 | 6/21/2022 | Public Health Ontario Laboratory |
|  | EPI_ISL_13671556 | 6/23/2022 | 7/6/2022 | RTPCR Lab Gurugram |
|  | EPI_ISL_13671557 | 6/23/2022 | 7/6/2022 | RTPCR Lab Gurugram |
|  | EPI_ISL_13671590 | 6/13/2022 | 7/6/2022 | RTPCR Lab Sirsa |
|  | EPI_ISL_13671592 | 6/15/2022 | 7/6/2022 | RTPCR Lab Sirsa |
|  | EPI_ISL_13671593 | 6/15/2022 | 7/6/2022 | RTPCR Lab Sirsa |
|  | EPI_ISL_13671612 | 6/23/2022 | 7/6/2022 | COVID-19 Laboratory Chuchot SNM Hospital |
|  | EPI_ISL_13667368 | 6/24/2022 | 7/6/2022 | Hopitaux Robert Schuman |
|  | EPI_ISL_13666977 | 6/4/2022 | 7/6/2022 | Directorate of Public Health and Preventive Medicine |
|  | EPI_ISL_13409385 | 5/26/2022 | 6/22/2022 | RT-PCR Lab, Regional Hospital Una |
|  | EPI_ISL_13660098 | 6/16/2022 | 7/6/2022 | DR. P BHASIN PATH LAB |
|  | EPI_ISL_13660044 | 6/17/2022 | 7/6/2022 | DR. P BHASIN PATH LAB |
|  | EPI_ISL_13409465 | 6/14/2022 | 6/22/2022 | CSIR-NEERI, Nagpur Covid-19 Testing Lab |
|  | EPI_ISL_13409444 | 6/13/2022 | 6/22/2022 | CSIR-NEERI, Nagpur Covid-19 Testing Lab |
|  | EPI_ISL_13659828 | 6/14/2022 | 7/6/2022 | DODA LAB |
|  | EPI_ISL_13659934 | 6/18/2022 | 7/6/2022 | MAX HOSPITAL |
|  | EPI_ISL_13659791 | 6/20/2022 | 7/6/2022 | MAX HOSPITAL |
|  | EPI_ISL_13692023 | 6/21/2022 | 7/7/2022 | Laboratorium Pathlab Jakarta Utara |
|  | EPI_ISL_13692495 | 6/27/2022 | 7/7/2022 | National Public Health Laboratory |
|  | EPI_ISL_13692500 | 6/22/2022 | 7/7/2022 | Belahiya Health desk |
|  | EPI_ISL_13692860 | 7/1/2022 | 7/7/2022 | Rosalind Franklin Laboratory |
|  | EPI_ISL_13695942 | 6/8/2022 | 7/7/2022 | AmpliGene laboratory, Ahmedabad |
|  | EPI_ISL_13705485 | 6/18/2022 | 7/8/2022 | Laboratory Corporation of America |
|  | EPI_ISL_13438754 | 6/15/2022 | 6/23/2022 | Southern Community Labs Chch |
|  | EPI_ISL_13438623 | 6/15/2022 | 6/23/2022 | Southern Community Labs Chch |
|  | EPI_ISL_13708103 | 6/25/2022 | 7/8/2022 | Laboratory Corporation of America |
|  | EPI_ISL_13708380 | 6/27/2022 | 7/8/2022 | Helix |
|  | EPI_ISL_13711333 | 6/7/2022 | 7/8/2022 | Supratech Micropath Diagnostics & Research laborat |
|  | EPI_ISL_13711285 | 6/13/2022 | 7/8/2022 | We Path Genexplore Laboratory, Vadodara |
|  | EPI_ISL_13711284 | 6/13/2022 | 7/8/2022 | We Path Genexplore Laboratory, Vadodara |
|  | EPI_ISL_13711410 | 6/16/2022 | 7/8/2022 | Unipath Speciality Laboaratory, Ahmedabad |
|  | EPI_ISL_13711378 | 6/13/2022 | 7/8/2022 | Eugene Molecular Lab, Ahmedabad |
|  | EPI_ISL_13711384 | 6/13/2022 | 7/8/2022 | Unipath Speciality Laboaratory, Ahmedabad |
|  | EPI_ISL_13711387 | 6/14/2022 | 7/8/2022 | Green Cross Pathology Laboratory, Navrangpura, Ahm |
|  | EPI_ISL_13455147 | 6/1/2022 | 6/24/2022 | Edmonton Provincial Lab |
|  | EPI_ISL_13461861 | 2022-06 | 6/25/2022 | Neuberg Anand Reference Laboratory, Bengaluru |
|  | EPI_ISL_13719283 | 6/27/2022 | 7/8/2022 | VRDL GMCH AURANGABAD |
|  | EPI_ISL_13719319 | 6/27/2022 | 7/8/2022 | VRDL GMCH AURANGABAD |
|  | EPI_ISL_13719299 | 6/27/2022 | 7/8/2022 | VRDL GMCH AURANGABAD |
|  | EPI_ISL_13719318 | 6/24/2022 | 7/8/2022 | VRDL GMCH AURANGABAD |
|  | EPI_ISL_13719313 | 6/27/2022 | 7/8/2022 | VRDL GMCH AURANGABAD |
|  | EPI_ISL_13719314 | 6/27/2022 | 7/8/2022 | VRDL GMCH AURANGABAD |
|  | EPI_ISL_13719279 | 6/27/2022 | 7/8/2022 | VRDL GMCH AURANGABAD |
|  | EPI_ISL_13719301 | 6/27/2022 | 7/8/2022 | VRDL GMCH AURANGABAD |
|  | EPI_ISL_13719304 | 6/27/2022 | 7/8/2022 | VRDL GMCH AURANGABAD |
|  | EPI_ISL_13719302 | 6/25/2022 | 7/8/2022 | VRDL GMCH AURANGABAD |
|  | EPI_ISL_13719306 | 6/25/2022 | 7/8/2022 | VRDL GMCH AURANGABAD |
|  | EPI_ISL_13719303 | 6/27/2022 | 7/8/2022 | VRDL GMCH AURANGABAD |
|  | EPI_ISL_13719297 | 6/27/2022 | 7/8/2022 | VRDL GMCH AURANGABAD |
|  | EPI_ISL_13719305 | 6/27/2022 | 7/8/2022 | VRDL GMCH AURANGABAD |
|  | EPI_ISL_13719292 | 6/27/2022 | 7/8/2022 | VRDL GMCH AURANGABAD |
|  | EPI_ISL_13719307 | 6/25/2022 | 7/8/2022 | VRDL GMCH AURANGABAD |
|  | EPI_ISL_13719285 | 6/27/2022 | 7/8/2022 | VRDL GMCH AURANGABAD |
|  | EPI_ISL_13719288 | 6/27/2022 | 7/8/2022 | VRDL GMCH AURANGABAD |
|  | EPI_ISL_13719282 | 6/25/2022 | 7/8/2022 | VRDL GMCH AURANGABAD |
|  | EPI_ISL_13719284 | 6/27/2022 | 7/8/2022 | VRDL GMCH AURANGABAD |
|  | EPI_ISL_13719320 | 6/24/2022 | 7/8/2022 | VRDL GMCH AURANGABAD |
|  | EPI_ISL_13719281 | 6/27/2022 | 7/8/2022 | VRDL GMCH AURANGABAD |
|  | EPI_ISL_13719312 | 6/27/2022 | 7/8/2022 | VRDL GMCH AURANGABAD |
|  | EPI_ISL_13725251 | 6/17/2022 | 7/8/2022 | Edmonton Provincial Lab |
|  | EPI_ISL_13727120 | 6/30/2022 | 7/8/2022 | Ministry of Health Turkey |
|  | EPI_ISL_13728165 | 6/15/2022 | 7/9/2022 | State Virus Research and Diagnostic Laboratory (VRDL), AIIMS Raipur |
|  | EPI_ISL_13728140 | 6/16/2022 | 7/9/2022 | State Virus Research and Diagnostic Laboratory (VRDL), AIIMS Raipur |
|  | EPI_ISL_13741530 | 6/22/2022 | 7/11/2022 | Respiratory Virus Unit, Microbiology Services Colindale, Public Health England |
|  | EPI_ISL_13747216 | 6/26/2022 | 7/11/2022 | Pandemic Response Lab - NYC |
|  | EPI_ISL_13752280 | 6/23/2022 | 7/11/2022 | State Virus Research and Diagnostic Laboratory (VRDL), AIIMS Raipur |
|  | EPI_ISL_13752274 | 6/25/2022 | 7/11/2022 | State Virus Research and Diagnostic Laboratory (VRDL), AIIMS Raipur |
|  | EPI_ISL_13458019 | 6/4/2022 | 6/25/2022 | Microbiological Diagnostic Unit - Public Health Laboratory (MDU-PHL) |
|  | EPI_ISL_13756684 | 6/20/2022 | 7/11/2022 | Outre Mer |
|  | EPI_ISL_13760714 | 6/26/2022 | 7/11/2022 | Dutch COVID-19 response team |
|  | EPI_ISL_13762812 | 7/7/2022 | 7/11/2022 | Genome Analysis Center, Yamanashi Central Hospital |
|  | EPI_ISL_13762799 | 7/1/2022 | 7/11/2022 | Genome Analysis Center, Yamanashi Central Hospital |
|  | EPI_ISL_13768912 | 7/4/2022 | 7/12/2022 | Rosalind Franklin Laboratory |
|  | EPI_ISL_13769147 | 6/26/2022 | 7/12/2022 | DODA |
|  | EPI_ISL_13769161 | 6/30/2022 | 7/12/2022 | DODA |
|  | EPI_ISL_13769162 | 6/30/2022 | 7/12/2022 | DODA |
|  | EPI_ISL_13769354 | 6/24/2022 | 7/12/2022 | STAR IMAGING & PATH LAB |
|  | EPI_ISL_13769352 | 6/24/2022 | 7/12/2022 | STAR IMAGING & PATH LAB |
|  | EPI_ISL_13769295 | 6/30/2022 | 7/12/2022 | P BHASIN LAB |
|  | EPI_ISL_13769323 | 6/22/2022 | 7/12/2022 | STAR IMAGING & PATH LAB |
|  | EPI_ISL_13769220 | 6/26/2022 | 7/12/2022 | P BHASIN LAB |
|  | EPI_ISL_13769376 | 6/26/2022 | 7/12/2022 | STAR IMAGING & PATH LAB |
|  | EPI_ISL_13769336 | 6/23/2022 | 7/12/2022 | STAR IMAGING & PATH LAB |
|  | EPI_ISL_13769228 | 6/26/2022 | 7/12/2022 | P BHASIN LAB |
|  | EPI_ISL_13769405 | 6/30/2022 | 7/12/2022 | RNA PATH LABS |
|  | EPI_ISL_13769291 | 6/30/2022 | 7/12/2022 | P BHASIN LAB |
|  | EPI_ISL_13769174 | 6/27/2022 | 7/12/2022 | DODA |
|  | EPI_ISL_13769384 | 6/26/2022 | 7/12/2022 | STAR IMAGING & PATH LAB |
|  | EPI_ISL_13765192 | 7/6/2022 | 7/12/2022 | Southern Community Labs Dunedin |
|  | EPI_ISL_13765186 | 6/21/2022 | 7/12/2022 | LabTests |
|  | EPI_ISL_13765207 | 7/4/2022 | 7/12/2022 | Canterbury Health Laboratories |
|  | EPI_ISL_13471039 | 6/20/2022 | 6/27/2022 | Rosalind Franklin Laboratory |
|  | EPI_ISL_13471048 | 6/20/2022 | 6/27/2022 | Rosalind Franklin Laboratory |
|  | EPI_ISL_13693330 | 6/29/2022 | 7/7/2022 | Rosalind Franklin Laboratory |
|  | EPI_ISL_13779708 | 6/20/2022 | 7/12/2022 | INSACOG-WB |
|  | EPI_ISL_13779720 | 6/21/2022 | 7/12/2022 | INSACOG-WB |
|  | EPI_ISL_13779383 | 6/20/2022 | 7/12/2022 | INSACOG-WB |
|  | EPI_ISL_13779363 | 6/17/2022 | 7/12/2022 | INSACOG-WB |
|  | EPI_ISL_13779418 | 6/19/2022 | 7/12/2022 | INSACOG-WB |
|  | EPI_ISL_13779398 | 6/14/2022 | 7/12/2022 | INSACOG-WB |
|  | EPI_ISL_13779698 | 6/19/2022 | 7/12/2022 | INSACOG-WB |
|  | EPI_ISL_13779665 | 6/22/2022 | 7/12/2022 | INSACOG-WB |
|  | EPI_ISL_13780265 | 6/23/2022 | 7/12/2022 | Laboratory Corporation of America |
|  | EPI_ISL_13779450 | 6/21/2022 | 7/12/2022 | INSACOG-WB |
|  | EPI_ISL_13779417 | 6/19/2022 | 7/12/2022 | INSACOG-WB |
|  | EPI_ISL_13779590 | 6/21/2022 | 7/12/2022 | INSACOG-WB |
|  | EPI_ISL_13779560 | 6/20/2022 | 7/12/2022 | INSACOG-WB |
|  | EPI_ISL_13779629 | 6/23/2022 | 7/12/2022 | INSACOG-WB |
|  | EPI_ISL_13779529 | 6/19/2022 | 7/12/2022 | INSACOG-WB |
|  | EPI_ISL_13574760 | 6/11/2022 | 7/1/2022 | AIIMS Bhopal VRDL/Translational Medicine - INSACOG |
|  | EPI_ISL_13498432 | 6/21/2022 | 6/28/2022 | Rosalind Franklin Laboratory |
|  | EPI_ISL_13498391 | 6/21/2022 | 6/28/2022 | Rosalind Franklin Laboratory |
|  | EPI_ISL_13498452 | 6/21/2022 | 6/28/2022 | Rosalind Franklin Laboratory |
|  | EPI_ISL_14217530 | 7/26/2022 | 8/3/2022 | Lifebrain Covid Labor GmbH |
|  | EPI_ISL_14217533 | 7/26/2022 | 8/3/2022 | Lifebrain Covid Labor GmbH |
|  | EPI_ISL_14217538 | 7/25/2022 | 8/3/2022 | Lifebrain Covid Labor GmbH |
|  | EPI_ISL_14217539 | 7/25/2022 | 8/3/2022 | Lifebrain Covid Labor GmbH |
|  | EPI_ISL_14217540 | 7/26/2022 | 8/3/2022 | Lifebrain Covid Labor GmbH |
|  | EPI_ISL_14217542 | 7/26/2022 | 8/3/2022 | Lifebrain Covid Labor GmbH |
|  | EPI_ISL_14217668 | 7/27/2022 | 8/3/2022 | Lifebrain Covid Labor GmbH |
|  | EPI_ISL_14221690 | 7/30/2022 | 8/3/2022 | Nebraska Public Health Laboratory |
|  | EPI_ISL_14222488 | 7/19/2022 | 8/3/2022 | Laboratoire de santÃ© publique du QuÃ©bec |
|  | EPI_ISL_14228299 | 7/14/2022 | 8/4/2022 | ILS, Bhubaneswar |
|  | EPI_ISL_14228303 | 7/16/2022 | 8/4/2022 | ILS, Bhubaneswar |
|  | EPI_ISL_14228311 | 7/18/2022 | 8/4/2022 | ILS, Bhubaneswar |
|  | EPI_ISL_14228316 | 7/10/2022 | 8/4/2022 | ILS, Bhubaneswar |
|  | EPI_ISL_14228319 | 7/11/2022 | 8/4/2022 | ILS, Bhubaneswar |
|  | EPI_ISL_14228320 | 7/11/2022 | 8/4/2022 | ILS, Bhubaneswar |
|  | EPI_ISL_14228324 | 7/13/2022 | 8/4/2022 | ILS, Bhubaneswar |
|  | EPI_ISL_14228332 | 7/15/2022 | 8/4/2022 | ILS, Bhubaneswar |
|  | EPI_ISL_14228336 | 7/16/2022 | 8/4/2022 | ILS, Bhubaneswar |
|  | EPI_ISL_14228359 | 7/4/2022 | 8/4/2022 | FMFMCH, Balasore |
|  | EPI_ISL_14228362 | 7/5/2022 | 8/4/2022 | FMFMCH, Balasore |
|  | EPI_ISL_14228364 | 7/6/2022 | 8/4/2022 | FMFMCH, Balasore |
|  | EPI_ISL_14228378 | 7/12/2022 | 8/4/2022 | FMFMCH, Balasore |
|  | EPI_ISL_14228381 | 7/13/2022 | 8/4/2022 | FMFMCH, Balasore |
|  | EPI_ISL_14228384 | 7/13/2022 | 8/4/2022 | FMFMCH, Balasore |
|  | EPI_ISL_14228390 | 7/16/2022 | 8/4/2022 | FMFMCH, Balasore |
|  | EPI_ISL_14228391 | 7/16/2022 | 8/4/2022 | FMFMCH, Balasore |
|  | EPI_ISL_14228393 | 7/9/2022 | 8/4/2022 | Deogarh, DHH |
|  | EPI_ISL_14228403 | 7/13/2022 | 8/4/2022 | Deogarh, DHH |
|  | EPI_ISL_14228406 | 7/14/2022 | 8/4/2022 | Deogarh, DHH |
|  | EPI_ISL_14228407 | 7/15/2022 | 8/4/2022 | Deogarh, DHH |
|  | EPI_ISL_14228409 | 7/17/2022 | 8/4/2022 | Deogarh, DHH |
|  | EPI_ISL_14228414 | 7/17/2022 | 8/4/2022 | Deogarh, DHH |
|  | EPI_ISL_14228417 | 7/19/2022 | 8/4/2022 | Deogarh, DHH |
|  | EPI_ISL_14228418 | 7/19/2022 | 8/4/2022 | Deogarh, DHH |
|  | EPI_ISL_14228419 | 7/18/2022 | 8/4/2022 | Deogarh, DHH |
|  | EPI_ISL_14228422 | 7/19/2022 | 8/4/2022 | Deogarh, DHH |
|  | EPI_ISL_14228425 | 7/20/2022 | 8/4/2022 | Deogarh, DHH |
|  | EPI_ISL_14228426 | 7/20/2022 | 8/4/2022 | Deogarh, DHH |
|  | EPI_ISL_14228437 | 7/22/2022 | 8/4/2022 | ILS, Bhubaneswar |
|  | EPI_ISL_14228438 | 7/23/2022 | 8/4/2022 | ILS, Bhubaneswar |
|  | EPI_ISL_14228440 | 7/16/2022 | 8/4/2022 | ILS, Bhubaneswar |
|  | EPI_ISL_14228444 | 7/11/2022 | 8/4/2022 | ILS, Bhubaneswar |
|  | EPI_ISL_14228450 | 7/12/2022 | 8/4/2022 | ILS, Bhubaneswar |
|  | EPI_ISL_14228453 | 7/13/2022 | 8/4/2022 | ILS, Bhubaneswar |
|  | EPI_ISL_14228455 | 7/14/2022 | 8/4/2022 | ILS, Bhubaneswar |
|  | EPI_ISL_14228458 | 7/15/2022 | 8/4/2022 | ILS, Bhubaneswar |
|  | EPI_ISL_14228459 | 7/15/2022 | 8/4/2022 | ILS, Bhubaneswar |
|  | EPI_ISL_14228465 | 7/16/2022 | 8/4/2022 | ILS, Bhubaneswar |
|  | EPI_ISL_14228468 | 7/17/2022 | 8/4/2022 | ILS, Bhubaneswar |
|  | EPI_ISL_14228470 | 7/17/2022 | 8/4/2022 | ILS, Bhubaneswar |
|  | EPI_ISL_14228471 | 7/18/2022 | 8/4/2022 | ILS, Bhubaneswar |
|  | EPI_ISL_14228473 | 7/18/2022 | 8/4/2022 | ILS, Bhubaneswar |
|  | EPI_ISL_14228479 | 7/19/2022 | 8/4/2022 | ILS, Bhubaneswar |
|  | EPI_ISL_14228482 | 7/19/2022 | 8/4/2022 | ILS, Bhubaneswar |
|  | EPI_ISL_14228486 | 7/20/2022 | 8/4/2022 | ILS, Bhubaneswar |
|  | EPI_ISL_14228487 | 7/20/2022 | 8/4/2022 | ILS, Bhubaneswar |
|  | EPI_ISL_14228495 | 7/7/2022 | 8/4/2022 | ILS, Bhubaneswar |
|  | EPI_ISL_14228498 | 7/12/2022 | 8/4/2022 | ILS, Bhubaneswar |
|  | EPI_ISL_14228501 | 7/14/2022 | 8/4/2022 | ILS, Bhubaneswar |
|  | EPI_ISL_14228514 | 6/29/2022 | 8/4/2022 | Nayagarh, DHH |
|  | EPI_ISL_14228517 | 7/5/2022 | 8/4/2022 | Nayagarh, DHH |
|  | EPI_ISL_14228535 | 7/20/2022 | 8/4/2022 | ILS, Bhubaneswar |
|  | EPI_ISL_14228538 | 7/20/2022 | 8/4/2022 | ILS, Bhubaneswar |
|  | EPI_ISL_14228543 | 7/22/2022 | 8/4/2022 | ILS, Bhubaneswar |
|  | EPI_ISL_14228544 | 7/21/2022 | 8/4/2022 | ILS, Bhubaneswar |
|  | EPI_ISL_14228545 | 7/21/2022 | 8/4/2022 | ILS, Bhubaneswar |
|  | EPI_ISL_14228547 | 7/22/2022 | 8/4/2022 | ILS, Bhubaneswar |
|  | EPI_ISL_14228564 | 7/23/2022 | 8/4/2022 | ILS, Bhubaneswar |
|  | EPI_ISL_14228565 | 7/25/2022 | 8/4/2022 | ILS, Bhubaneswar |
|  | EPI_ISL_14228569 | 7/15/2022 | 8/4/2022 | ILS, Bhubaneswar |
|  | EPI_ISL_14228570 | 7/15/2022 | 8/4/2022 | ILS, Bhubaneswar |
|  | EPI_ISL_14228571 | 7/16/2022 | 8/4/2022 | ILS, Bhubaneswar |
|  | EPI_ISL_14228576 | 7/17/2022 | 8/4/2022 | ILS, Bhubaneswar |
|  | EPI_ISL_14228577 | 7/18/2022 | 8/4/2022 | ILS, Bhubaneswar |
|  | EPI_ISL_14228580 | 7/18/2022 | 8/4/2022 | ILS, Bhubaneswar |
|  | EPI_ISL_14228581 | 7/19/2022 | 8/4/2022 | ILS, Bhubaneswar |
|  | EPI_ISL_14228582 | 7/19/2022 | 8/4/2022 | ILS, Bhubaneswar |
|  | EPI_ISL_14228585 | 7/21/2022 | 8/4/2022 | ILS, Bhubaneswar |
|  | EPI_ISL_14228587 | 7/21/2022 | 8/4/2022 | ILS, Bhubaneswar |
|  | EPI_ISL_14228591 | 7/21/2022 | 8/4/2022 | ILS, Bhubaneswar |
|  | EPI_ISL_14228593 | 7/22/2022 | 8/4/2022 | ILS, Bhubaneswar |
|  | EPI_ISL_14228597 | 7/24/2022 | 8/4/2022 | ILS, Bhubaneswar |
|  | EPI_ISL_14228600 | 7/9/2022 | 8/4/2022 | SLNMCH, Koraput |
|  | EPI_ISL_14228602 | 7/13/2022 | 8/4/2022 | SLNMCH, Koraput |
|  | EPI_ISL_14228604 | 7/15/2022 | 8/4/2022 | SLNMCH, Koraput |
|  | EPI_ISL_14228606 | 7/21/2022 | 8/4/2022 | PRMMCH, Baripada |
|  | EPI_ISL_14228609 | 7/24/2022 | 8/4/2022 | PRMMCH, Baripada |
|  | EPI_ISL_14228614 | 7/24/2022 | 8/4/2022 | ILS, Bhubaneswar |
|  | EPI_ISL_14228619 | 7/25/2022 | 8/4/2022 | ILS, Bhubaneswar |
|  | EPI_ISL_14228620 | 7/25/2022 | 8/4/2022 | ILS, Bhubaneswar |
|  | EPI_ISL_14228621 | 7/25/2022 | 8/4/2022 | ILS, Bhubaneswar |
|  | EPI_ISL_14228624 | 7/26/2022 | 8/4/2022 | ILS, Bhubaneswar |
|  | EPI_ISL_14228626 | 7/27/2022 | 8/4/2022 | ILS, Bhubaneswar |
|  | EPI_ISL_14228630 | 7/27/2022 | 8/4/2022 | ILS, Bhubaneswar |
|  | EPI_ISL_14228631 | 7/27/2022 | 8/4/2022 | ILS, Bhubaneswar |
|  | EPI_ISL_14228632 | 7/27/2022 | 8/4/2022 | ILS, Bhubaneswar |
|  | EPI_ISL_14235009 | 7/16/2022 | 8/4/2022 | Respiratory Virus Unit, Microbiology Services Colindale, Public Health England |
|  | EPI_ISL_14241161 | 7/2/2022 | 8/4/2022 | Laboratorio de Referencia Nacional de Virus Inmunoprevenibles. Centro Nacional de Salud Publica. Instituto Nacional de Salud Peru. |
|  | EPI_ISL_14244219 | 7/30/2022 | 8/4/2022 | Clinical Microbiology Laboratory, Tel Aviv Sourasky Medical Center |
|  | EPI_ISL_14246715 | 7/28/2022 | 8/4/2022 | Clinical Microbiology Laboratory, Tel Aviv Sourasky Medical Center |
|  | EPI_ISL_14247350 | 7/26/2022 | 8/4/2022 | Clinical Microbiology Laboratory, Tel Aviv Sourasky Medical Center |
|  | EPI_ISL_14248054 | 7/25/2022 | 8/4/2022 | Clinical Microbiology Laboratory, Tel Aviv Sourasky Medical Center |
|  | EPI_ISL_14248095 | 7/25/2022 | 8/4/2022 | Clinical Microbiology Laboratory, Tel Aviv Sourasky Medical Center |
|  | EPI_ISL_14248908 | 7/22/2022 | 8/4/2022 | Clinical Microbiology Laboratory, Tel Aviv Sourasky Medical Center |
|  | EPI_ISL_14250609 | 7/15/2022 | 8/4/2022 | Microbiological Diagnostic Unit - Public Health Laboratory (MDU-PHL) |
|  | EPI_ISL_14255482 | 7/21/2022 | 8/5/2022 | Division of Emerging Infectious Diseases, Bureau of Infectious Diseases Diagnosis Control, Korea Disease Control and Prevention Agency |
|  | EPI_ISL_14258171 | 7/25/2022 | 8/5/2022 | State Hygienic Laboratory at the University of Iowa |
|  | EPI_ISL_14259742 | 7/21/2022 | 8/5/2022 | Tokyo Metropolitan Institute of Public Health |
|  | EPI_ISL_14260657 | 7/3/2022 | 8/5/2022 | BJMC |
|  | EPI_ISL_14260665 | 7/1/2022 | 8/5/2022 | BJMC |
|  | EPI_ISL_14260666 | 7/1/2022 | 8/5/2022 | BJMC |
|  | EPI_ISL_14260667 | 7/1/2022 | 8/5/2022 | BJMC |
|  | EPI_ISL_14260669 | 7/1/2022 | 8/5/2022 | BJMC |
|  | EPI_ISL_14260670 | 7/1/2022 | 8/5/2022 | BJMC |
|  | EPI_ISL_14260672 | 7/1/2022 | 8/5/2022 | BJMC |
|  | EPI_ISL_14260673 | 7/1/2022 | 8/5/2022 | BJMC |
|  | EPI_ISL_14260751 | 7/2/2022 | 8/5/2022 | BJMC |
|  | EPI_ISL_14260760 | 7/1/2022 | 8/5/2022 | BJMC |
|  | EPI_ISL_14260763 | 7/1/2022 | 8/5/2022 | BJMC |
|  | EPI_ISL_14260764 | 7/1/2022 | 8/5/2022 | BJMC |
|  | EPI_ISL_14260769 | 7/1/2022 | 8/5/2022 | BJMC |
|  | EPI_ISL_14260771 | 7/1/2022 | 8/5/2022 | BJMC |
|  | EPI_ISL_14260787 | 7/2/2022 | 8/5/2022 | BJMC |
|  | EPI_ISL_14260801 | 7/3/2022 | 8/5/2022 | BJMC |
|  | EPI_ISL_14260805 | 7/3/2022 | 8/5/2022 | BJMC |
|  | EPI_ISL_14260817 | 7/4/2022 | 8/5/2022 | BJMC |
|  | EPI_ISL_14260841 | 6/20/2022 | 8/5/2022 | Genepath |
|  | EPI_ISL_14260849 | 6/21/2022 | 8/5/2022 | Genepath |
|  | EPI_ISL_14260865 | 6/28/2022 | 8/5/2022 | Genepath |
|  | EPI_ISL_14260878 | 7/2/2022 | 8/5/2022 | Genepath |
|  | EPI_ISL_14260883 | 7/4/2022 | 8/5/2022 | Genepath |
|  | EPI_ISL_14260887 | 7/5/2022 | 8/5/2022 | Genepath |
|  | EPI_ISL_14260895 | 7/8/2022 | 8/5/2022 | Genepath |
|  | EPI_ISL_14260899 | 7/8/2022 | 8/5/2022 | Genepath |
|  | EPI_ISL_14260901 | 7/8/2022 | 8/5/2022 | Genepath |
|  | EPI_ISL_14260903 | 7/9/2022 | 8/5/2022 | Genepath |
|  | EPI_ISL_14260905 | 7/9/2022 | 8/5/2022 | Genepath |
|  | EPI_ISL_14260918 | 6/21/2022 | 8/5/2022 | Genepath |
|  | EPI_ISL_14260940 | 6/29/2022 | 8/5/2022 | Genepath |
|  | EPI_ISL_14260955 | 7/2/2022 | 8/5/2022 | Genepath |
|  | EPI_ISL_14260962 | 7/4/2022 | 8/5/2022 | Genepath |
|  | EPI_ISL_14260963 | 7/4/2022 | 8/5/2022 | Genepath |
|  | EPI_ISL_14260969 | 7/5/2022 | 8/5/2022 | Genepath |
|  | EPI_ISL_14260970 | 7/5/2022 | 8/5/2022 | Genepath |
|  | EPI_ISL_14260971 | 7/5/2022 | 8/5/2022 | Genepath |
|  | EPI_ISL_14260976 | 7/6/2022 | 8/5/2022 | Genepath |
|  | EPI_ISL_14260977 | 7/6/2022 | 8/5/2022 | Genepath |
|  | EPI_ISL_14260982 | 7/7/2022 | 8/5/2022 | Genepath |
|  | EPI_ISL_14260985 | 7/9/2022 | 8/5/2022 | Genepath |
|  | EPI_ISL_14260990 | 7/11/2022 | 8/5/2022 | Genepath |
|  | EPI_ISL_14260991 | 7/11/2022 | 8/5/2022 | Genepath |
|  | EPI_ISL_14260992 | 7/11/2022 | 8/5/2022 | Genepath |
|  | EPI_ISL_14260997 | 6/23/2022 | 8/5/2022 | Noble Hospital |
|  | EPI_ISL_14261002 | 6/24/2022 | 8/5/2022 | Noble Hospital |
|  | EPI_ISL_14263858 | 7/19/2022 | 8/5/2022 | Laboratory Corporation of America |
|  | EPI_ISL_14264255 | 7/19/2022 | 8/5/2022 | Laboratory Corporation of America |
|  | EPI_ISL_14266563 | 7/15/2022 | 8/5/2022 | Quest Diagnostics Incorporated |
|  | EPI_ISL_14267276 | 7/16/2022 | 8/5/2022 | Quest Diagnostics Incorporated |
|  | EPI_ISL_14269451 | 2022 | 8/5/2022 | Pathology and Laboratory Medicine Institute, Cleveland Clinic, Ohio, USA |
|  | EPI_ISL_14269600 | 7/18/2022 | 8/5/2022 | Quest Diagnostics Incorporated |
|  | EPI_ISL_14274603 | 7/17/2022 | 8/5/2022 | Quest Diagnostics Incorporated |
|  | EPI_ISL_14274604 | 7/17/2022 | 8/5/2022 | Quest Diagnostics Incorporated |
|  | EPI_ISL_14275521 | 7/19/2022 | 8/5/2022 | Quest Diagnostics Incorporated |
|  | EPI_ISL_14275561 | 7/19/2022 | 8/5/2022 | Quest Diagnostics Incorporated |
|  | EPI_ISL_14276124 | 7/26/2022 | 8/5/2022 | CENTRACARE LABORATORY SERVICES |
|  | EPI_ISL_14277619 | 7/6/2022 | 8/5/2022 | Virginia Division of Consolidated Laboratory Services |
|  | EPI_ISL_14277654 | 7/19/2022 | 8/5/2022 | GA Department of Public Health |
|  | EPI_ISL_14277993 | 7/22/2022 | 8/5/2022 | Northeastern LSTC |
|  | EPI_ISL_14277999 | 7/21/2022 | 8/5/2022 | Northeastern LSTC |
|  | EPI_ISL_14281729 | 7/23/2022 | 8/5/2022 | Helix |
|  | EPI_ISL_14282311 | 7/24/2022 | 8/5/2022 | Helix |
|  | EPI_ISL_14282741 | 7/25/2022 | 8/5/2022 | Helix |
|  | EPI_ISL_14283690 | 7/20/2022 | 8/5/2022 | Laboratory Corporation of America |
|  | EPI_ISL_14285381 | 7/28/2022 | 8/5/2022 | Pathology West - NSW Health Pathology |
|  | EPI_ISL_14285457 | 7/24/2022 | 8/5/2022 | Australian Clinical Labs (formerly Healthscope Pathology) |
|  | EPI_ISL_14285512 | 7/23/2022 | 8/5/2022 | Pathology North - Royal North Shore Hospital - NSW Health Pathology |
|  | EPI_ISL_14285531 | 7/25/2022 | 8/5/2022 | Histopath Diagnostic Specialists |
|  | EPI_ISL_14285540 | 7/25/2022 | 8/5/2022 | Histopath Diagnostic Specialists |
|  | EPI_ISL_14285574 | 7/25/2022 | 8/5/2022 | Australian Clinical Labs (formerly Healthscope Pathology) |
|  | EPI_ISL_14285577 | 7/25/2022 | 8/5/2022 | Australian Clinical Labs (formerly Healthscope Pathology) |
|  | EPI_ISL_14285583 | 7/26/2022 | 8/5/2022 | Australian Clinical Labs (formerly Healthscope Pathology) |
|  | EPI_ISL_14285685 | 7/27/2022 | 8/5/2022 | Histopath Diagnostic Specialists |
|  | EPI_ISL_14285686 | 7/27/2022 | 8/5/2022 | Histopath Diagnostic Specialists |
|  | EPI_ISL_14288037 | 8/2/2022 | 8/6/2022 | Presidio Ospedaliero "Madonna delle Grazie" di Matera |
|  | EPI_ISL_14288793 | 7/17/2022 | 8/5/2022 | Provincial Laboratory for Public Health (ProvLab) - North |
|  | EPI_ISL_14289144 | 7/29/2022 | 8/6/2022 | Rosalind Franklin Laboratory |
|  | EPI_ISL_14290261 | 7/6/2022 | 8/6/2022 | P BHASIN |
|  | EPI_ISL_14290266 | 7/26/2022 | 8/6/2022 | ACTION CANCER HOSPITAL |
|  | EPI_ISL_14290271 | 7/5/2022 | 8/6/2022 | MAX HOSPITAL |
|  | EPI_ISL_14290275 | 7/13/2022 | 8/6/2022 | MAX HOSPITAL |
|  | EPI_ISL_14290276 | 7/14/2022 | 8/6/2022 | MAX HOSPITAL |
|  | EPI_ISL_14290277 | 7/14/2022 | 8/6/2022 | MAX HOSPITAL |
|  | EPI_ISL_14290278 | 7/18/2022 | 8/6/2022 | MAX HOSPITAL |
|  | EPI_ISL_14290428 | 7/26/2022 | 8/6/2022 | MATA CHANAN DEVI HOSPITAL |
|  | EPI_ISL_14290431 | 7/18/2022 | 8/6/2022 | ACTION CANCER HOSPITAL |
|  | EPI_ISL_14290432 | 7/17/2022 | 8/6/2022 | MAX HOSPITAL |
|  | EPI_ISL_14290438 | 7/19/2022 | 8/6/2022 | UNIPATH DIAGNOSTICS |
|  | EPI_ISL_14290445 | 7/4/2022 | 8/6/2022 | MAX HOSPITAL |
|  | EPI_ISL_14290449 | 7/18/2022 | 8/6/2022 | MAX HOSPITAL |
|  | EPI_ISL_14290455 | 7/4/2022 | 8/6/2022 | MAX HOSPITAL |
|  | EPI_ISL_14290460 | 7/18/2022 | 8/6/2022 | MAX HOSPITAL |
|  | EPI_ISL_14290462 | 7/7/2022 | 8/6/2022 | P BHASIN |
|  | EPI_ISL_14290464 | 7/25/2022 | 8/6/2022 | ACTION CANCER HOSPITAL |
|  | EPI_ISL_14290476 | 7/5/2022 | 8/6/2022 | MAHARAJA AGRASEN HOSPITAL |
|  | EPI_ISL_14290478 | 6/29/2022 | 8/6/2022 | MAX HOSPITAL |
|  | EPI_ISL_14290481 | 7/3/2022 | 8/6/2022 | P BHASIN |
|  | EPI_ISL_14290497 | 6/29/2022 | 8/6/2022 | MAX HOSPITAL |
|  | EPI_ISL_14290498 | 7/15/2022 | 8/6/2022 | GENESTRINGS DIAGNOSTIC CENTRE |
|  | EPI_ISL_14290506 | 7/4/2022 | 8/6/2022 | P BHASIN |
|  | EPI_ISL_14290507 | 7/17/2022 | 8/6/2022 | MAX HOSPITAL |
|  | EPI_ISL_14290510 | 7/15/2022 | 8/6/2022 | MAX HOSPITAL |
|  | EPI_ISL_14290517 | 7/4/2022 | 8/6/2022 | P BHASIN |
|  | EPI_ISL_14290522 | 7/10/2022 | 8/6/2022 | MAX HOSPITAL |
|  | EPI_ISL_14290524 | 6/30/2022 | 8/6/2022 | MAX HOSPITAL |
|  | EPI_ISL_14290544 | 7/3/2022 | 8/6/2022 | MAX HOSPITAL |
|  | EPI_ISL_14290603 | 7/25/2022 | 8/6/2022 | Dr. Yoshitaka Tamura Department of Clinical Laboratory, Osaka Habikino Medical Center |
|  | EPI_ISL_14290848 | 7/18/2022 | 8/6/2022 | IDSP, Kamrup Metro (ULTRACARE DIAGNOSTIC CENTRE) |
|  | EPI_ISL_14290849 | 7/18/2022 | 8/6/2022 | IDSP, Kamrup Metro (ULTRACARE DIAGNOSTIC CENTRE) |
|  | EPI_ISL_14290858 | 7/19/2022 | 8/6/2022 | IDSP, Kamrup Metro (ULTRACARE DIAGNOSTIC CENTRE) |
|  | EPI_ISL_14290859 | 7/21/2022 | 8/6/2022 | IDSP, Kamrup Metro (ULTRACARE DIAGNOSTIC CENTRE) |
|  | EPI_ISL_14290867 | 7/18/2022 | 8/6/2022 | IDSP, Kamrup Metro (GMCH) |
|  | EPI_ISL_14290873 | 7/20/2022 | 8/6/2022 | IDSP, Kamrup Metro (GMCH) |
|  | EPI_ISL_14290875 | 7/20/2022 | 8/6/2022 | IDSP, Kamrup Metro (GMCH) |
|  | EPI_ISL_14290876 | 7/19/2022 | 8/6/2022 | IDSP, Kamrup Metro (GMCH) |
|  | EPI_ISL_14291703 | 7/11/2022 | 8/7/2022 | B J Government Medical College and Sassoon General Hospitals, Pune |
|  | EPI_ISL_14291704 | 7/11/2022 | 8/7/2022 | B J Government Medical College and Sassoon General Hospitals, Pune |
|  | EPI_ISL_14291705 | 7/11/2022 | 8/7/2022 | B J Government Medical College and Sassoon General Hospitals, Pune |
|  | EPI_ISL_14291708 | 7/12/2022 | 8/7/2022 | B J Government Medical College and Sassoon General Hospitals, Pune |
|  | EPI_ISL_14291709 | 7/13/2022 | 8/7/2022 | B J Government Medical College and Sassoon General Hospitals, Pune |
|  | EPI_ISL_14291711 | 7/14/2022 | 8/7/2022 | B J Government Medical College and Sassoon General Hospitals, Pune |
|  | EPI_ISL_14291713 | 7/14/2022 | 8/7/2022 | B J Government Medical College and Sassoon General Hospitals, Pune |
|  | EPI_ISL_14291715 | 7/15/2022 | 8/7/2022 | B J Government Medical College and Sassoon General Hospitals, Pune |
|  | EPI_ISL_14291716 | 7/15/2022 | 8/7/2022 | B J Government Medical College and Sassoon General Hospitals, Pune |
|  | EPI_ISL_14291717 | 7/16/2022 | 8/7/2022 | B J Government Medical College and Sassoon General Hospitals, Pune |
|  | EPI_ISL_14291720 | 7/18/2022 | 8/7/2022 | B J Government Medical College and Sassoon General Hospitals, Pune |
|  | EPI_ISL_14291721 | 7/20/2022 | 8/7/2022 | B J Government Medical College and Sassoon General Hospitals, Pune |
|  | EPI_ISL_14291722 | 7/20/2022 | 8/7/2022 | B J Government Medical College and Sassoon General Hospitals, Pune |
|  | EPI_ISL_14291723 | 7/23/2022 | 8/7/2022 | B J Government Medical College and Sassoon General Hospitals, Pune |
|  | EPI_ISL_14291725 | 7/25/2022 | 8/7/2022 | B J Government Medical College and Sassoon General Hospitals, Pune |
|  | EPI_ISL_14294852 | 7/26/2022 | 8/8/2022 | Laboratory Corporation of America |
|  | EPI_ISL_14294933 | 7/26/2022 | 8/8/2022 | Laboratory Corporation of America |
|  | EPI_ISL_14295558 | 7/27/2022 | 8/8/2022 | Helix |
|  | EPI_ISL_14295657 | 7/28/2022 | 8/8/2022 | Helix |
|  | EPI_ISL_14296296 | 7/24/2022 | 8/8/2022 | Lighthouse Lab in Glasgow |
|  | EPI_ISL_14300447 | 7/13/2022 | 8/8/2022 | Tokyo Metropolitan Institute of Public Health |
|  | EPI_ISL_14300449 | 7/15/2022 | 8/8/2022 | Tokyo Metropolitan Institute of Public Health |
|  | EPI_ISL_14300450 | 7/19/2022 | 8/8/2022 | Tokyo Metropolitan Institute of Public Health |
|  | EPI_ISL_14300467 | 7/11/2022 | 8/8/2022 | Tokyo Metropolitan Institute of Public Health |
|  | EPI_ISL_14300468 | 7/12/2022 | 8/8/2022 | Tokyo Metropolitan Institute of Public Health |
|  | EPI_ISL_14300469 | 7/12/2022 | 8/8/2022 | Tokyo Metropolitan Institute of Public Health |
|  | EPI_ISL_14300470 | 7/12/2022 | 8/8/2022 | Tokyo Metropolitan Institute of Public Health |
|  | EPI_ISL_14300471 | 7/9/2022 | 8/8/2022 | Tokyo Metropolitan Institute of Public Health |
|  | EPI_ISL_14301290 | 7/26/2022 | 8/8/2022 | Respiratory Virus Unit, Microbiology Services Colindale, Public Health England |
|  | EPI_ISL_14301573 | 7/26/2022 | 8/8/2022 | Respiratory Virus Unit, Microbiology Services Colindale, Public Health England |
|  | EPI_ISL_14302969 | 7/21/2022 | 8/8/2022 | INSACOG-WB |
|  | EPI_ISL_14302971 | 7/19/2022 | 8/8/2022 | INSACOG-WB |
|  | EPI_ISL_14302972 | 7/5/2022 | 8/8/2022 | INSACOG-WB |
|  | EPI_ISL_14302974 | 7/8/2022 | 8/8/2022 | INSACOG-WB |
|  | EPI_ISL_14302976 | 7/22/2022 | 8/8/2022 | INSACOG-WB |
|  | EPI_ISL_14302977 | 7/15/2022 | 8/8/2022 | INSACOG-WB |
|  | EPI_ISL_14302979 | 7/16/2022 | 8/8/2022 | INSACOG-WB |
|  | EPI_ISL_14302984 | 7/19/2022 | 8/8/2022 | INSACOG-WB |
|  | EPI_ISL_14302992 | 7/11/2022 | 8/8/2022 | INSACOG-WB |
|  | EPI_ISL_14302998 | 7/11/2022 | 8/8/2022 | INSACOG-WB |
|  | EPI_ISL_14303002 | 7/11/2022 | 8/8/2022 | INSACOG-WB |
|  | EPI_ISL_14303011 | 7/11/2022 | 8/8/2022 | INSACOG-WB |
|  | EPI_ISL_14303015 | 7/11/2022 | 8/8/2022 | INSACOG-WB |
|  | EPI_ISL_14303020 | 7/11/2022 | 8/8/2022 | INSACOG-WB |
|  | EPI_ISL_14303022 | 7/5/2022 | 8/8/2022 | INSACOG-WB |
|  | EPI_ISL_14303031 | 7/5/2022 | 8/8/2022 | INSACOG-WB |
|  | EPI_ISL_14303034 | 7/8/2022 | 8/8/2022 | INSACOG-WB |
|  | EPI_ISL_14303035 | 7/13/2022 | 8/8/2022 | INSACOG-WB |
|  | EPI_ISL_14303037 | 7/15/2022 | 8/8/2022 | INSACOG-WB |
|  | EPI_ISL_14303039 | 7/15/2022 | 8/8/2022 | INSACOG-WB |
|  | EPI_ISL_14303042 | 7/15/2022 | 8/8/2022 | INSACOG-WB |
|  | EPI_ISL_14303043 | 7/15/2022 | 8/8/2022 | INSACOG-WB |
|  | EPI_ISL_14303044 | 7/15/2022 | 8/8/2022 | INSACOG-WB |
|  | EPI_ISL_14303045 | 7/15/2022 | 8/8/2022 | INSACOG-WB |
|  | EPI_ISL_14303046 | 7/15/2022 | 8/8/2022 | INSACOG-WB |
|  | EPI_ISL_14303047 | 7/15/2022 | 8/8/2022 | INSACOG-WB |
|  | EPI_ISL_14303051 | 7/15/2022 | 8/8/2022 | INSACOG-WB |
|  | EPI_ISL_14303053 | 7/15/2022 | 8/8/2022 | INSACOG-WB |
|  | EPI_ISL_14303054 | 7/15/2022 | 8/8/2022 | INSACOG-WB |
|  | EPI_ISL_14303055 | 7/16/2022 | 8/8/2022 | INSACOG-WB |
|  | EPI_ISL_14303056 | 7/16/2022 | 8/8/2022 | INSACOG-WB |
|  | EPI_ISL_14303059 | 7/18/2022 | 8/8/2022 | INSACOG-WB |
|  | EPI_ISL_14303061 | 7/18/2022 | 8/8/2022 | INSACOG-WB |
|  | EPI_ISL_14303071 | 7/20/2022 | 8/8/2022 | INSACOG-WB |
|  | EPI_ISL_14303073 | 7/22/2022 | 8/8/2022 | INSACOG-WB |
|  | EPI_ISL_14303074 | 7/22/2022 | 8/8/2022 | INSACOG-WB |
|  | EPI_ISL_14303075 | 7/22/2022 | 8/8/2022 | INSACOG-WB |
|  | EPI_ISL_14303076 | 7/22/2022 | 8/8/2022 | INSACOG-WB |
|  | EPI_ISL_14303077 | 7/22/2022 | 8/8/2022 | INSACOG-WB |
|  | EPI_ISL_14303093 | 7/10/2022 | 8/8/2022 | INSACOG-WB |
|  | EPI_ISL_14303099 | 7/18/2022 | 8/8/2022 | INSACOG-WB |
|  | EPI_ISL_14303100 | 7/18/2022 | 8/8/2022 | INSACOG-WB |
|  | EPI_ISL_14303106 | 7/13/2022 | 8/8/2022 | INSACOG-WB |
|  | EPI_ISL_14303107 | 7/14/2022 | 8/8/2022 | INSACOG-WB |
|  | EPI_ISL_14303112 | 7/18/2022 | 8/8/2022 | INSACOG-WB |
|  | EPI_ISL_14303115 | 7/12/2022 | 8/8/2022 | INSACOG-WB |
|  | EPI_ISL_14303126 | 7/18/2022 | 8/8/2022 | INSACOG-WB |
|  | EPI_ISL_14303128 | 7/18/2022 | 8/8/2022 | INSACOG-WB |
|  | EPI_ISL_14303130 | 6/26/2022 | 8/8/2022 | INSACOG-WB |
|  | EPI_ISL_14303143 | 7/16/2022 | 8/8/2022 | INSACOG-WB |
|  | EPI_ISL_14303148 | 7/16/2022 | 8/8/2022 | INSACOG-WB |
|  | EPI_ISL_14303153 | 7/18/2022 | 8/8/2022 | INSACOG-WB |
|  | EPI_ISL_14303154 | 7/18/2022 | 8/8/2022 | INSACOG-WB |
|  | EPI_ISL_14303156 | 7/18/2022 | 8/8/2022 | INSACOG-WB |
|  | EPI_ISL_14303162 | 7/18/2022 | 8/8/2022 | INSACOG-WB |
|  | EPI_ISL_14303164 | 7/18/2022 | 8/8/2022 | INSACOG-WB |
|  | EPI_ISL_14303172 | 7/19/2022 | 8/8/2022 | INSACOG-WB |
|  | EPI_ISL_14303173 | 7/19/2022 | 8/8/2022 | INSACOG-WB |
|  | EPI_ISL_14303174 | 7/19/2022 | 8/8/2022 | INSACOG-WB |
|  | EPI_ISL_14303185 | 7/20/2022 | 8/8/2022 | INSACOG-WB |
|  | EPI_ISL_14303186 | 7/20/2022 | 8/8/2022 | INSACOG-WB |
|  | EPI_ISL_14303189 | 7/21/2022 | 8/8/2022 | INSACOG-WB |
|  | EPI_ISL_14303190 | 7/21/2022 | 8/8/2022 | INSACOG-WB |
|  | EPI_ISL_14303192 | 7/21/2022 | 8/8/2022 | INSACOG-WB |
|  | EPI_ISL_14303198 | 7/22/2022 | 8/8/2022 | INSACOG-WB |
|  | EPI_ISL_14303199 | 7/22/2022 | 8/8/2022 | INSACOG-WB |
|  | EPI_ISL_14303200 | 7/18/2022 | 8/8/2022 | INSACOG-WB |
|  | EPI_ISL_14303203 | 7/4/2022 | 8/8/2022 | INSACOG-WB |
|  | EPI_ISL_14303210 | 7/2/2022 | 8/8/2022 | INSACOG-WB |
|  | EPI_ISL_14303221 | 7/1/2022 | 8/8/2022 | INSACOG-WB |
|  | EPI_ISL_14303266 | 7/18/2022 | 8/8/2022 | INSACOG-WB |
|  | EPI_ISL_14303269 | 7/18/2022 | 8/8/2022 | INSACOG-WB |
|  | EPI_ISL_14303275 | 7/19/2022 | 8/8/2022 | INSACOG-WB |
|  | EPI_ISL_14303278 | 7/20/2022 | 8/8/2022 | INSACOG-WB |
|  | EPI_ISL_14303286 | 7/20/2022 | 8/8/2022 | INSACOG-WB |
|  | EPI_ISL_14303287 | 7/20/2022 | 8/8/2022 | INSACOG-WB |
|  | EPI_ISL_14303298 | 7/14/2022 | 8/8/2022 | INSACOG-WB |
|  | EPI_ISL_14303308 | 7/15/2022 | 8/8/2022 | INSACOG-WB |
|  | EPI_ISL_14303309 | 7/16/2022 | 8/8/2022 | INSACOG-WB |
|  | EPI_ISL_14303370 | 7/16/2022 | 8/8/2022 | INSACOG-Manipur |
|  | EPI_ISL_14303371 | 7/16/2022 | 8/8/2022 | INSACOG-Manipur |
|  | EPI_ISL_14303372 | 7/17/2022 | 8/8/2022 | INSACOG-Manipur |
|  | EPI_ISL_14303380 | 7/20/2022 | 8/8/2022 | INSACOG-Manipur |
|  | EPI_ISL_14303388 | 7/5/2022 | 8/8/2022 | INSACOG-Manipur |
|  | EPI_ISL_14303394 | 7/10/2022 | 8/8/2022 | INSACOG-Manipur |
|  | EPI_ISL_14303403 | 7/13/2022 | 8/8/2022 | INSACOG-Manipur |
|  | EPI_ISL_14303412 | 7/14/2022 | 8/8/2022 | INSACOG-Manipur |
|  | EPI_ISL_14303414 | 7/15/2022 | 8/8/2022 | INSACOG-Manipur |
|  | EPI_ISL_14303430 | 7/20/2022 | 8/8/2022 | INSACOG-Manipur |
|  | EPI_ISL_14303613 | 7/6/2022 | 8/8/2022 | INSACOG-WB |
|  | EPI_ISL_14303615 | 7/6/2022 | 8/8/2022 | INSACOG-WB |
|  | EPI_ISL_14303617 | 7/6/2022 | 8/8/2022 | INSACOG-WB |
|  | EPI_ISL_14303621 | 7/6/2022 | 8/8/2022 | INSACOG-WB |
|  | EPI_ISL_14303625 | 7/6/2022 | 8/8/2022 | INSACOG-WB |
|  | EPI_ISL_14303628 | 7/6/2022 | 8/8/2022 | INSACOG-WB |
|  | EPI_ISL_14303632 | 7/6/2022 | 8/8/2022 | INSACOG-WB |
|  | EPI_ISL_14303637 | 7/7/2022 | 8/8/2022 | INSACOG-WB |
|  | EPI_ISL_14303642 | 7/7/2022 | 8/8/2022 | INSACOG-WB |
|  | EPI_ISL_14303644 | 7/7/2022 | 8/8/2022 | INSACOG-WB |
|  | EPI_ISL_14303645 | 7/7/2022 | 8/8/2022 | INSACOG-WB |
|  | EPI_ISL_14303647 | 7/7/2022 | 8/8/2022 | INSACOG-WB |
|  | EPI_ISL_14303648 | 7/7/2022 | 8/8/2022 | INSACOG-WB |
|  | EPI_ISL_14303650 | 7/7/2022 | 8/8/2022 | INSACOG-WB |
|  | EPI_ISL_14303654 | 7/7/2022 | 8/8/2022 | INSACOG-WB |
|  | EPI_ISL_14303659 | 7/8/2022 | 8/8/2022 | INSACOG-WB |
|  | EPI_ISL_14303660 | 7/8/2022 | 8/8/2022 | INSACOG-WB |
|  | EPI_ISL_14303662 | 7/8/2022 | 8/8/2022 | INSACOG-WB |
|  | EPI_ISL_14303666 | 7/8/2022 | 8/8/2022 | INSACOG-WB |
|  | EPI_ISL_14303670 | 7/8/2022 | 8/8/2022 | INSACOG-WB |
|  | EPI_ISL_14303674 | 7/8/2022 | 8/8/2022 | INSACOG-WB |
|  | EPI_ISL_14303678 | 7/8/2022 | 8/8/2022 | INSACOG-WB |
|  | EPI_ISL_14303681 | 7/8/2022 | 8/8/2022 | INSACOG-WB |
|  | EPI_ISL_14303683 | 7/8/2022 | 8/8/2022 | INSACOG-WB |
|  | EPI_ISL_14303687 | 7/8/2022 | 8/8/2022 | INSACOG-WB |
|  | EPI_ISL_14303694 | 7/8/2022 | 8/8/2022 | INSACOG-WB |
|  | EPI_ISL_14303695 | 7/8/2022 | 8/8/2022 | INSACOG-WB |
|  | EPI_ISL_14303705 | 7/8/2022 | 8/8/2022 | INSACOG-WB |
|  | EPI_ISL_14303707 | 7/8/2022 | 8/8/2022 | INSACOG-WB |
|  | EPI_ISL_14303708 | 7/8/2022 | 8/8/2022 | INSACOG-WB |
|  | EPI_ISL_14303710 | 7/8/2022 | 8/8/2022 | INSACOG-WB |
|  | EPI_ISL_14303715 | 7/11/2022 | 8/8/2022 | INSACOG-WB |
|  | EPI_ISL_14303719 | 7/11/2022 | 8/8/2022 | INSACOG-WB |
|  | EPI_ISL_14303721 | 7/11/2022 | 8/8/2022 | INSACOG-WB |
|  | EPI_ISL_14303723 | 7/11/2022 | 8/8/2022 | INSACOG-WB |
|  | EPI_ISL_14303736 | 7/12/2022 | 8/8/2022 | INSACOG-WB |
|  | EPI_ISL_14303747 | 7/12/2022 | 8/8/2022 | INSACOG-WB |
|  | EPI_ISL_14303755 | 7/13/2022 | 8/8/2022 | INSACOG-WB |
|  | EPI_ISL_14303765 | 7/6/2022 | 8/8/2022 | INSACOG-WB |
|  | EPI_ISL_14303767 | 7/6/2022 | 8/8/2022 | INSACOG-WB |
|  | EPI_ISL_14303769 | 7/6/2022 | 8/8/2022 | INSACOG-WB |
|  | EPI_ISL_14303781 | 7/6/2022 | 8/8/2022 | INSACOG-WB |
|  | EPI_ISL_14303784 | 7/6/2022 | 8/8/2022 | INSACOG-WB |
|  | EPI_ISL_14303788 | 7/6/2022 | 8/8/2022 | INSACOG-WB |
|  | EPI_ISL_14303812 | 6/28/2022 | 8/8/2022 | INSACOG-WB |
|  | EPI_ISL_14303813 | 6/28/2022 | 8/8/2022 | INSACOG-WB |
|  | EPI_ISL_14303815 | 6/29/2022 | 8/8/2022 | INSACOG-WB |
|  | EPI_ISL_14303820 | 6/29/2022 | 8/8/2022 | INSACOG-WB |
|  | EPI_ISL_14303833 | 7/1/2022 | 8/8/2022 | INSACOG-WB |
|  | EPI_ISL_14303836 | 7/1/2022 | 8/8/2022 | INSACOG-WB |
|  | EPI_ISL_14303840 | 7/2/2022 | 8/8/2022 | INSACOG-WB |
|  | EPI_ISL_14303842 | 7/2/2022 | 8/8/2022 | INSACOG-WB |
|  | EPI_ISL_14303843 | 7/2/2022 | 8/8/2022 | INSACOG-WB |
|  | EPI_ISL_14303845 | 7/2/2022 | 8/8/2022 | INSACOG-WB |
|  | EPI_ISL_14303846 | 7/2/2022 | 8/8/2022 | INSACOG-WB |
|  | EPI_ISL_14303856 | 7/4/2022 | 8/8/2022 | INSACOG-WB |
|  | EPI_ISL_14303858 | 7/4/2022 | 8/8/2022 | INSACOG-WB |
|  | EPI_ISL_14303860 | 7/4/2022 | 8/8/2022 | INSACOG-WB |
|  | EPI_ISL_14303869 | 7/5/2022 | 8/8/2022 | INSACOG-WB |
|  | EPI_ISL_14303871 | 7/5/2022 | 8/8/2022 | INSACOG-WB |
|  | EPI_ISL_14303886 | 7/4/2022 | 8/8/2022 | INSACOG-WB |
|  | EPI_ISL_14303910 | 7/6/2022 | 8/8/2022 | INSACOG-Sikkim |
|  | EPI_ISL_14303928 | 7/12/2022 | 8/8/2022 | INSACOG-Sikkim |
|  | EPI_ISL_14303939 | 7/13/2022 | 8/8/2022 | INSACOG-Sikkim |
|  | EPI_ISL_14303944 | 7/13/2022 | 8/8/2022 | INSACOG-Sikkim |
|  | EPI_ISL_14303962 | 7/7/2022 | 8/8/2022 | INSACOG-Sikkim |
|  | EPI_ISL_14303969 | 7/14/2022 | 8/8/2022 | INSACOG-Sikkim |
|  | EPI_ISL_14303975 | 7/14/2022 | 8/8/2022 | INSACOG-Sikkim |
|  | EPI_ISL_14303976 | 7/14/2022 | 8/8/2022 | INSACOG-Sikkim |
|  | EPI_ISL_14303977 | 7/14/2022 | 8/8/2022 | INSACOG-Sikkim |
|  | EPI_ISL_14303981 | 7/14/2022 | 8/8/2022 | INSACOG-Sikkim |
|  | EPI_ISL_14303987 | 7/14/2022 | 8/8/2022 | INSACOG-Sikkim |
|  | EPI_ISL_14303990 | 7/9/2022 | 8/8/2022 | INSACOG-Sikkim |
|  | EPI_ISL_14303995 | 7/9/2022 | 8/8/2022 | INSACOG-Sikkim |
|  | EPI_ISL_14304002 | 7/13/2022 | 8/8/2022 | INSACOG-WB |
|  | EPI_ISL_14304006 | 7/15/2022 | 8/8/2022 | INSACOG-WB |
|  | EPI_ISL_14304009 | 7/9/2022 | 8/8/2022 | INSACOG-WB |
|  | EPI_ISL_14304010 | 7/9/2022 | 8/8/2022 | INSACOG-WB |
|  | EPI_ISL_14304016 | 7/9/2022 | 8/8/2022 | INSACOG-WB |
|  | EPI_ISL_14304024 | 7/7/2022 | 8/8/2022 | INSACOG-WB |
|  | EPI_ISL_14304028 | 7/11/2022 | 8/8/2022 | INSACOG-WB |
|  | EPI_ISL_14304039 | 7/8/2022 | 8/8/2022 | INSACOG-WB |
|  | EPI_ISL_14304047 | 7/9/2022 | 8/8/2022 | INSACOG-WB |
|  | EPI_ISL_14304049 | 7/9/2022 | 8/8/2022 | INSACOG-WB |
|  | EPI_ISL_14304062 | 7/1/2022 | 8/8/2022 | INSACOG-WB |
|  | EPI_ISL_14304074 | 7/6/2022 | 8/8/2022 | INSACOG-WB |
|  | EPI_ISL_14304076 | 7/7/2022 | 8/8/2022 | INSACOG-WB |
|  | EPI_ISL_14304077 | 7/7/2022 | 8/8/2022 | INSACOG-WB |
|  | EPI_ISL_14304087 | 7/5/2022 | 8/8/2022 | INSACOG-WB |
|  | EPI_ISL_14304093 | 7/6/2022 | 8/8/2022 | INSACOG-WB |
|  | EPI_ISL_14304096 | 7/7/2022 | 8/8/2022 | INSACOG-WB |
|  | EPI_ISL_14304103 | 7/7/2022 | 8/8/2022 | INSACOG-WB |
|  | EPI_ISL_14304105 | 7/7/2022 | 8/8/2022 | INSACOG-WB |
|  | EPI_ISL_14304106 | 7/7/2022 | 8/8/2022 | INSACOG-WB |
|  | EPI_ISL_14304132 | 7/4/2022 | 8/8/2022 | INSACOG-WB |
|  | EPI_ISL_14304147 | 7/4/2022 | 8/8/2022 | INSACOG-WB |
|  | EPI_ISL_14304155 | 7/4/2022 | 8/8/2022 | INSACOG-WB |
|  | EPI_ISL_14304171 | 7/4/2022 | 8/8/2022 | INSACOG-WB |
|  | EPI_ISL_14304174 | 7/4/2022 | 8/8/2022 | INSACOG-WB |
|  | EPI_ISL_14305796 | 7/27/2022 | 8/8/2022 | Pandemic Response Lab - NYC |
|  | EPI_ISL_14307810 | 8/2/2022 | 8/8/2022 | Genetica Molecular and Subdepartamento de Virologia ISP Chile |
